# Design and Synthesis of Structurally Modified Analogs of 24*Z*-Isomasticadienonic Acid with Enhanced Anti-Proliferative Activity

**DOI:** 10.3390/molecules30234572

**Published:** 2025-11-27

**Authors:** Panagiota Stamou, Leentje Persoons, Dominique Schols, Steven De Jonghe, Leandros A. Skaltsounis, Ioannis K. Kostakis

**Affiliations:** 1 Department of Pharmacy, Division of Pharmaceutical Chemistry, National and Kapodistrian University of Athens, Panepistimiopolis Zografou, 15771 Athens, Greece; pstamou@pharm.uoa.gr; 2 Molecular Genetics and Therapeutics in Virology and Oncology Research Group, Department of Microbiology, Immunology and Transplantation, Rega Institute for Medical Research, KU Leuven, Herestraat 49, Box 1048, 3000 Leuven, Belgium; leentje.persoons@kuleuven.be; 3 Molecular, Structural and Translational Virology Research Group, Department of Microbiology, Immunology and Transplantation, Rega Institute for Medical Research, KU Leuven, Herestraat 49, Box 1049, 3000 Leuven, Belgium; dominique.schols@kuleuven.be (D.S.); steven.dejonghe@kuleuven.be (S.D.J.); 4 Department of Pharmacy, Division of Pharmacognosy and Natural Products Chemistry, National and Kapodistrian University of Athens, Panepistimiopolis Zografou, 15771 Athens, Greece; skaltsounis@pharm.uoa.gr

**Keywords:** 24*Z*-isomasticadienonic acid, analogs, semi-synthesis, Chios Mastic Gum, triterpenic acids, anti-proliferative activity

## Abstract

Τriterpenic acids represent a prominent class of bioactive compounds, with a wide range of biological properties, including anti-inflammatory, antiviral, and anticancer effects. Among them, 24*Z*-isomasticadienonic acid (IMNA), a major constituent of Chios Mastic Gum, has attracted little attention compared with other well-studied triterpenes such as oleanolic or betulinic acid, largely because its isolation in sufficient purity and quantity was only recently achieved. In this study, a series of IMNA analogs was synthesized through targeted modifications at the A-ring. These included the introduction of heteroatoms at position 2, the incorporation of heterocyclic rings such as an oxazole and a thiazole, and rearrangements of the ring structure. The new compounds were evaluated for their antiproliferative activity against a diverse panel of cancer cell lines (Capan-1, HCT-116, LN-229, NCI-H460, DND-41, HL-60, K-562, Z-138). Among the synthesized analogs, compounds **3**, **7** and **9** demonstrated selective anticancer activity toward the Capan-1 cell line, whereas compounds **6** and **10** exhibited broad-spectrum cytotoxic effects across multiple cancer cell lines. Overall, these findings highlight IMNA as a promising scaffold for anticancer drug design and demonstrate the value of A-ring modifications in improving activity and selectivity.

## 1. Introduction

**Figure 1:**
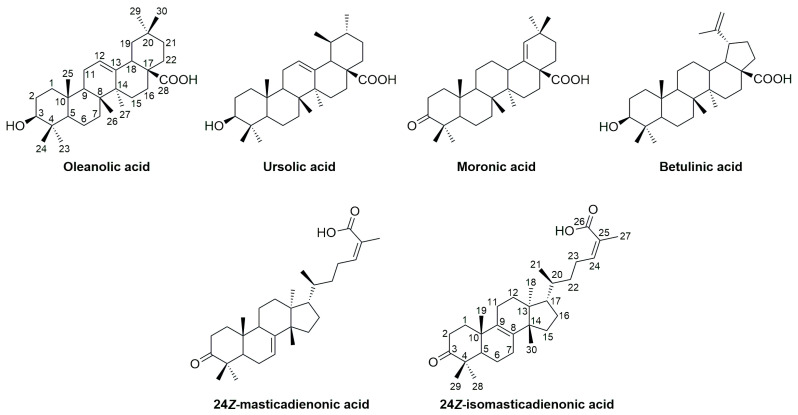
Chemical structures of triterpenic acids.

In recent years, natural products have played a significant role in medicine, serving not only as a source of potential chemotherapeutic agents but also as lead compounds for the semi-synthesis or total synthesis of new drugs [1,2,3]. Characteristic examples are the pentacyclic and tetracyclic triterpenic acids, a class of chemical compounds found in plants, marine organisms, bacteria and fungi, known for their diverse biological activities, including anti-inflammatory, antiviral and anticancer effects [4,5,6,7]. Among the most studied triterpenic acids are oleanolic, ursolic, moronic and betulinic acids, which have attracted significant scientific interest due to their broad spectrum of biological activities and their widespread occurrence in numerous plant species [8,9,10,11,12] (Figure 1).

Despite their notable bioactivity and ability to interact with biological systems through multiple mechanisms, triterpenes exhibit certain limitations [13,14,15]. One of the major challenges is their low aqueous solubility, which significantly reduces their absorption and bioavailability in vivo. Consequently, although triterpenes often demonstrate potent activity in vitro, they often show limited therapeutic efficacy when evaluated in vivo. Therefore, the development of more potent analogs based on the bioactive triterpene scaffold is required [16].

**Figure 2:**
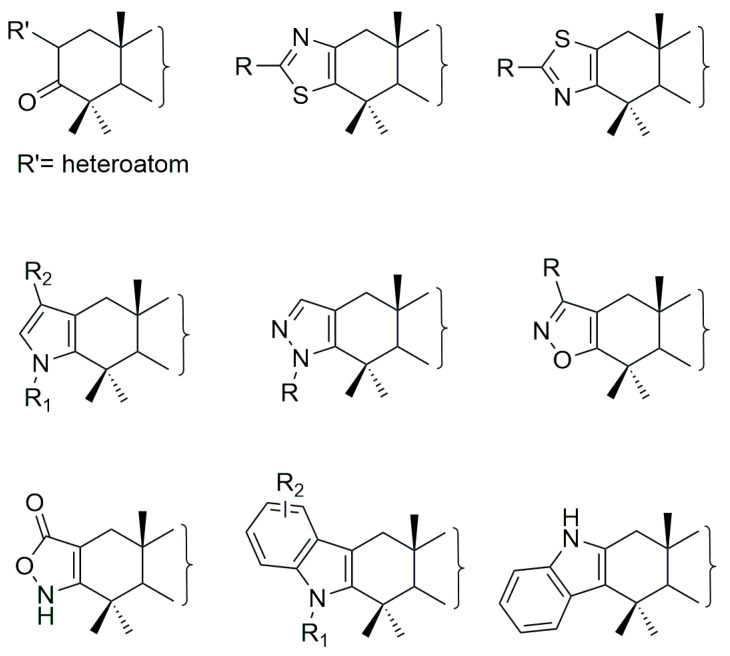
Analogs of triterpenic acids with anti-cancer activity.

In recent years, numerous triterpene analogs have been synthesized, many of which exhibit potent biological activities, amongst others, anticancer effects. Reflecting their therapeutic potential, an increasing number of patents has been filed to protect these newly developed triterpenoid compounds ([6,17,18] and references herein). A prominent group among these includes substituted derivatives with a heteroatom at position 2 of the A-ring, as well as those incorporating heterocyclic or polycyclic structures fused to the triterpenoid backbone [16,19,20] (Figure 2). Specifically, due to their chemical structure, heterocyclic compounds are often more stable and resistant to metabolic degradation. As a result, they remain active in the body for longer periods, enhancing therapeutic efficacy. Additionally, the presence of aromatic systems, heteroatoms and heterocyclic rings enables absorption in the ultraviolet (UV) region, facilitating compound detection and monitoring. Consequently, these structural modifications offer clear advantages in terms of both anti-proliferative potential and clinical applicability [21].

24*Z*-isomasticadienonic acid (IMNA, **1**) is a tetracyclic triterpene and one of the main constituents of Chios Mastic Gum (CMG), along with 24*Z*-masticadienonic acid (MNA) [22]. Although IMNA is naturally abundant in the resin, its isolation in high purity and sufficient quantities remained particularly challenging until recently. As a result, its biological properties remain underexplored and only a limited number of analogs have been synthesized to date [23]. In contrast, other triterpenic acids—such as oleanolic, betulinic, and ursolic acid—have been extensively studied, and numerous analogs of these compounds have been developed [9,24,25,26]. These observations underscore the relevance of investigating IMNA’s anti-proliferative potential and support the rationale for designing novel analogs based on its scaffold.

Therefore, the aim of the study is to develop IMNA analogs for the purpose of investigating their potential activity against various cancer cell lines. Drawing from the literature and the chemical structures of similar bioactive triterpenoid analogs, a series of IMNA derivatives was designed, featuring changes on the A-ring. Specifically, these modifications include the introduction of heterocyclic rings such as an oxazole and thiazole, along with substitution by a heteroatom at position 2. Furthermore, considering that the structure and size of the A-ring significantly influence the molecule’s conformation and, consequently, its biological activity, additional analogs possessing a rearrangement of the A-ring were also synthesized. These modifications aim to enhance pharmacological properties and provide insight into structure–activity relationships.

## 2. Results and Discussion

### 2.1. Chemistry

#### 2.1.1. Semi-Synthesis of 24*Z*-Isomasticadienonic Acid (**1**)

**Scheme 1:**
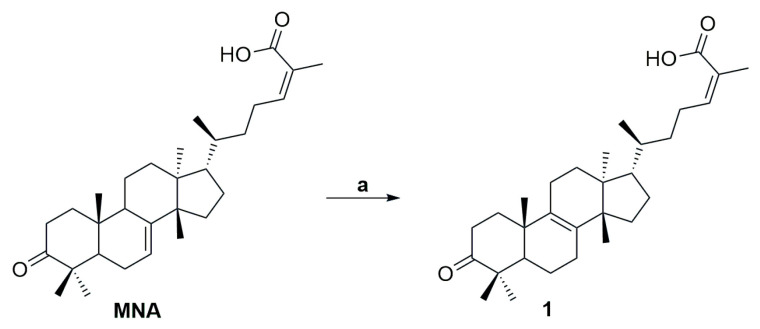
Isomerization of MNA: **a**. BBr_3_, DCM, 0 °C, 15 min. (yield 83%).

Compound **1** (IMNA) was synthesized following a procedure previously reported by our group. Briefly, 24*Z*-masticadienonic acid (MNA) was isolated from the resin of CMG by preparative high-performance liquid chromatography (preparative HPLC). In the presence of BBr_3_, isomerization of the internal double bond of MNA took place, affording compound **1** (Scheme 1) [23,27].

#### 2.1.2. Synthesis of 24*Z*-Isomasticadienonic Acid Analogs

**Scheme 2:**
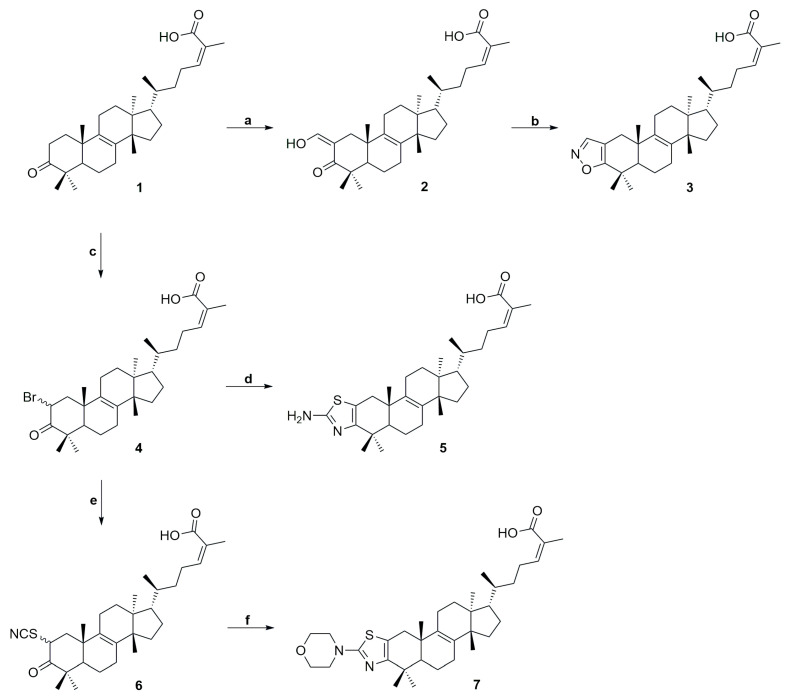
Reagents and conditions: (**a**) HCOOEt, NaOEt, rt, 17 h (yield 90%); (**b**) NH_2_OH.HCl, H_2_O, EtOH, 80 °C, 2 h (yield 76%); (**c**) PyHBr_3_, ACN, rt, 12 h (yield 66%); (**d**) thiourea, abs. EtOH, 5 d (yield 80%); (**e**) KSCN, DMSO, 90 °C, 4 h (yield 89%); (**f**). morpholinium acetate, CHCl_3_, r.t., 7 d (yield 40%).

The synthesis of compounds **2**–**7** is outlined in Scheme 2. Treatment of IMNA with ethyl formate in the presence of sodium ethoxide afforded 2-hydroxymethylen-3-oxotirucalla-8,24*Z*-dien-26-oic acid (**2**) via a Claisen condensation [28]. Subsequent reaction with hydroxylamine hydrochloride in absolute ethanol, afforded the corresponding oxime, which upon intramolecular cyclization yielded the isoxazole derivative **3** [29,30]. Consequently, treatment of **1** with pyridinium perbromide (PyHBr_3_) afforded **4** as a mixture of α- and β-epimers in a 6.5:3.5 ratio, complicating NMR analysis and complete structural assignment [31]. Nonetheless, the ^1^H NMR spectrum displayed a peak at 5.10 ppm, integrating for one proton, characteristic of the proton at position 2 (Figure S13, see Supplementary Material). Bromine incorporation was further confirmed by mass spectrometry, where under negative ionization mode, the characteristic isotopic pattern of bromine was observed together with a pseudo-molecular ion at *m*/*z* 531.2479, consistent with the desired product. Subsequent treatment of **4** with thiourea in absolute ethanol afforded the aminothiazole derivative **5** [32,33]. Its structural elucidation was supported by the HMBC spectrum (Figure S21, see Supplementary Material). The protons at C-1, resonating at 2.61 and 2.30 ppm, correlated with the quaternary carbons C-2 and C-3 of the aminothiazole ring, observed at 115.92 and 149.91 ppm, respectively. Notably, C-3 showed HMBC correlations with the protons of the methyl group at positions 28 and 29. In addition, treatment of **4** with potassium thiocyanate in DMSO yielded 2-thiocyanate-3-oxotirucalla-8,24*Z*-dien-26-oic acid (**6**). Compound **6**, similarly to **4**, was obtained as an inseparable mixture of epimers (α and β) in a ratio of 6.7:3.3. Its structure was confirmed by mass spectrometry, which in negative ionization mode showed a pseudo-molecular ion at *m*/*z* 510.3027, consistent with the expected product. Finally, the morpholino derivative **7** was obtained by treatment of compound **6** with morpholinium acetate [34].

**Scheme 3:**
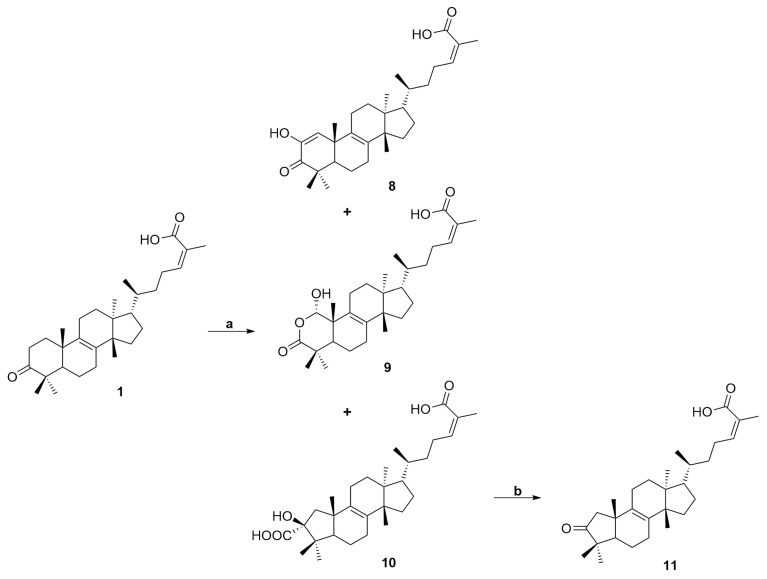
Reagents and conditions: (**a**) i. *t*-BuOK, *t*-BuOH, O_2_, 40 °C, 2 h, ii. KOH, 5% H_2_O, 80 °C, 24 h; (**b**) Pb(C_2_H_3_O_2_)_4_, AcOH, rt, 5 h (yield 62%).

The synthesis of compounds **8**–**11** is depicted in Scheme 3. Treatment of compound **1** in *t*-BuOH with potassium *tert*-butoxide under an oxygen atmosphere, followed by the addition of aqueous potassium hydroxide, afforded the oxidized compound 2-hydroxy-3-oxotirucalla-8,24*Z*-dien-26-oic acid (**8**), as well as 1α-hydroxy-3-oxa-nor-tirucalla-8,24*Z*-dien-26-oic acid (**9**) and 3β-hydroxy-1(2→3)-abeotirucalla-8,24*Z*-dien-26-oic acid (**10**), through benzilic acid rearrangement [35]. Compounds **8** and **10** have been previously reported, whereas **9** represents a novel analog of the naturally occurring 24*Z*-isomasticadienonic acid [23]. Structural elucidation of compound **9** was performed using mass spectrometry and one- and two-dimensional NMR spectroscopy. In the mass spectrum under negative ionization mode, a pseudo-molecular ion was detected at *m*/*z* 471.3152, corresponding to the molecular formula C_29_H_43_O_5_. This indicates the presence of two additional oxygen atoms and one carbon atom less, when compared with **1**. The ^13^C NMR spectrum confirmed these features, showing both the expected number of carbon signals and chemical shifts consistent with additional oxygenated carbons (Figure S39, see Supplementary Material). The proton at C-1 (δ_H_ 5.49 ppm), as well as the methyl protons at C-29 and C-30, exhibited HMBC correlations to the carbonyl carbon at C-3 (δ_C_ 181.4 ppm). The hydroxyl group at C-1 accounted for both the de-shielding of the proton at this position (5.49 ppm) and the chemical shift of C-1 at 102.1 ppm. Furthermore, NOESY data established the stereochemistry of the hydroxyl substituent at C-1, with the proton showing NOE correlations to the methyl group at C-19 (Figure S40, see Supplementary Material). Finally, treatment of the α-carboxylic acid **10** with palladium tetraacetate triggered decarboxylation, followed by oxidation of the hydroxyl group, furnishing the final compound **11** [36].

### 2.2. Antitumoral Evaluation

IMNA and the newly synthesized derivatives were evaluated for potential antitumoral activity using a panel of solid cancers, including pancreatic adenocarcinoma (Capan-1), colorectal carcinoma (HCT-116), glioblastoma (LN-229) and lung carcinoma (NCI–H460). In parallel, the cytotoxicity versus various hematological cancers, including acute lymphoblastic leukemia (DND-41), acute myeloid leukemia (HL-60), chronic myeloid leukemia (K-562) and non-Hodgkin’s lymphoma (Z-138) was also determined. Etoposide, used as positive control, displayed potent antiproliferative activity against all cancer cell lines (Table 1). Notably, the Capan-1 pancreatic cell line emerged as the most sensitive, with all compounds showing cytotoxicity in the 6–39 µM range. Compounds **3**, **7**, and **9** demonstrated selective cytotoxicity toward Capan-1, with minimal effects on the other tested cell lines. Compounds **6** and **10** showed broader antiproliferative activity, affecting most solid and hematological cancer lines. Compounds **4**, **5,** and IMNA exhibited intermediate activity across multiple cell lines, whereas compounds **8** and **11** displayed a more limited and restricted cytotoxic profile.

**Figure 3:**
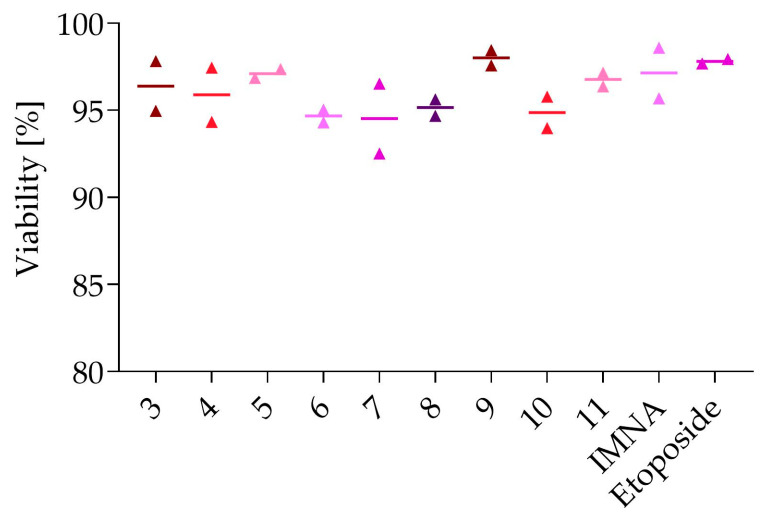
Cell viability of PBMCs following 72 h exposure to all compounds. Compounds **3**–**11** and IMNA were tested at 50 µM, and etoposide at 10 µM. Datapoints shown are duplicate values and mean.

To ensure a selective antitumoral effect, the effect of the compounds on the viability of normal (i.e., non-cancerous) peripheral blood mononuclear cells (PBMCs) was studied (Figure 3). Etoposide (as reference compound) at a concentration of 10 µM did not display toxicity to PMBC. At a concentration of 50 µM, none of the analogues (compounds **3**–**11** and IMNA) decreased the viability of PBMCs, suggesting a selective effect on cancer cells.

## 3. Materials and Methods

Ethyl acetate (EtOAc), *n*-Hexane, methanol (MeOH), dichloromethane (DCM) and cyclohexane (c-hexane) used for extraction and purification were of analytical grade (Fisher Scientific, Loughborough, Belgium), while water (H_2_O) was distilled. For the pH adjustment sodium hydroxide pellets (NaOH-penta CHEMICALS UNITED) and hydrochloric acid (HCl-analytical grade; Fisher Scientific, Loughborough, Belgium) were used. Acetonitrile (ACN, Avantor Performance Materials, Gliwice, Poland), and H_2_O (Fisher Scientific, Loughborough, Belgium) used for preparative High-Performance Liquid Chromatography (prep-HPLC) analysis were of HPLC grade. Reaction progresses were monitored by thin-layer chromatography on pre-coated silica gel 60 F254 plates from Merck (0.25 mm thickness) and were visualized on 254 and 366 nm (UV lamp). Flash chromatography was performed using Merck silica gel 60A (0.040–0.060 mm) (Merck, Kenilworth, NJ, USA) with the specified solvent system, typically applying gradients of increasing polarity. All commercially available reagents were purchased from Alfa Aesar and used without any further purification. The isolation of MNA was performed using preparative RP-HPLC (Buchi, Flawil, Switzerland, Pure C-850 FlashPrep) hyphenated to a Photo Diode Array (PDA) detector. All solvent evaporations were performed using a rotary evaporator (Buchi) with a water bath at 40 °C. Optical rotations were measured with a Jasco P-2000 Polarimeter (Jasco, Tokyo, Japan). Measurements were performed at a wavelength of 589 nm (Na D-line) and a path length of 50 mm. ^1^H NMR, ^13^C NMR and 2D spectra were recorded on a Bruker Avance III 600 MHz spectrometer (Bruker Biospin AG, Faellanden, Switzerland) and on a Bruker Avance NEO 400 MHz spectrometer (Bruker, Faellanden, Switzerland) in deuterated solvents (see Supplementary Materials). Chemical shifts are expressed as δ values in parts per million (ppm), and the coupling constants (*J*) are given in Hertz (Hz). The signals of ^1^H and ^13^C NMR spectra were unambiguously assigned by using 2D NMR techniques: ^1^H-^1^H COSY, HSQC-DEPT 135, NOESY and HMBC experiments. The signals are reported as follows: (s = singlet, d = doublet, t = triplet, m = multiplet, br = broad). HRMS spectra were obtained on LTQ-Orbitrap Discovery Mass Spectrometer (Thermo Scientific, Brehmen, Germany) and on a Triple TOF 5600+ ABSciex (Sciex, Framingham, MA, USA).

### 3.1. Semi-Synthesis of 24Z-Isomasticadienonic Acid

#### 3.1.1. Isolation of 24*Z*-Masticadienonic Acid (MNA)

MNA was obtained from the Total Mastic Extract Without Polymer (TMEWP) after removal of cis-1,4-poly-β-myrcene. From 10.0 g of TMEWP, 5.1 g of the acidic fraction of triterpenes (AF) were recovered through liquid–liquid extraction with gradual pH adjustment. In brief, TMEWP was partitioned in a separatory funnel between a polar phase (20% NaOH in H_2_O/MeOH, 1:1, pH = 11) and a non-polar phase (*n*-hexane/EtOAc, 8:2). The aqueous phase was then acidified with 1 N HCl to pH 3 and extracted with EtOAc, affording the AF. Finally, a RP-HPLC-PDA method was employed using a gradient elution system consisting of ACN and H_2_O, through which 25.0 mg of pure MNA were isolated from 250.0 mg of AF per injection. All separations were carried out on a Buchi Pure C-850 FlashPrep system coupled to a PDA detector, using a reversed-phase column (Agilent 5 Prep-C18, Agilent Technologies, Santa Clara, CA, USA, 50 × 50 mm). NMR data were consistent with the literature [22,23].

#### 3.1.2. Synthesis of 24Z-Isomasticadienonic Acid (**1**)

MNA (60.0 mg, 0.13 mmol) was dissolved in anhydrous DCM (23.0 mL), and BBr_3_ (1.0 eq) was added at 0 °C under an argon atmosphere. The reaction mixture was stirred for 15 min at 0 °C, after which water was added and the product extracted with EtOAc. The organic layer was dried over anhydrous Na_2_SO_4_ and concentrated under reduced pressure to afford 50.0 mg of IMNA as a white solid. (yield = 83%). NMR data were consistent with the literature [23].

### 3.2. Synthesis of 24Z-Isomasticadienonic Acid Analogs

#### 3.2.1. Synthesis of 2-Hydroxymethylen-3-oxotirucalla-8,24*Z*-dien-26-oic Acid (**2**)

To a solution of **1** (124.4 mg, 0.274 mmol) in HCOOEt (5.0 mL) under argon, was added NaOEt (25.0 eq) and the mixture was stirred at room temperature for 17 h. Upon completion of the reaction, H_2_O was added, followed by acidification with 6 N HCl and extraction with EtOAc. After drying the organic phase over anhydrous Na_2_SO_4_ and concentrating it under reduced pressure, 120.0 mg of **2** were obtained as a white solid (yield = 90%). ${\left[a\right]}_{D}^{25}$ +15 (c 0.243, CHCl_3_); ^1^H NMR (600 MHz, CDCl_3_) δ (ppm) 8.68 (1H, s, H-31), 6.09 (1H, t, H-24), 2.61–2.52 (1H, m, H-23 a), 2.50–2.42 (1H, m, H-23b), 2.41 (1H, d, *J* = 14.92 Hz, H-1a), 2.10 (1H, m, H-11a), 2.09 (2H, m, H-16), 2.09 (1H, d, *J* = 14.27 Hz, H-1b), 1.95 (1H, m, H-11b), 1.92 (3H, s, CH_3_-27), 1.75 (2H, m, H-12), 1.63 (1H, m, H-6a), 1.55 (2H, m, H-7), 1.53 (1H, m, H-15a), 1.52 (1H, m, H-17), 1.45 (1H, m, H-5), 1.44 (1H, m, H-20), 1.38 (1H, m, H-6b), 1.33 (1H, m, H-15b), 1.20 (3H, s, CH_3_-29), 1.12 (3H, s, CH_3_-28), 0.939 (3H, s, CH_3_-21), 0.931 (3H, s, CH_3_-19), 0.90 (3H, s, CH_3_-30), 0.77 (3H, s, CH_3_-18); ^13^C NMR (151 MHz, CDCl_3_) δ (ppm) 190.80 (C, C-3), 189.340 (C, CH-31), 172.77 (C, C-26), 147.51 (CH, C-24), 135.79 (C, C-8), 131.63 (C, C-9), 125.90 (C, C-25), 106.31 (C, C-2), 50.38 (C, C-13), 50.28 (CH, C-17), 49.21 (CH, C-5), 44.38 (C, C-14), 40.55 (C, C-4), 37.03 (C, C-10), 36.66 (CH, C-20), 36.27 (CH_2_, C-1), 36.07 (CH_2_, C-22), 30.90 (CH_2_, C-12), 29.95 (CH_2_, C-7), 28.72 (CH_3_, C-29), 28.24 (CH_2_, C-15), 27.65 (CH_2_,C-11), 27.09 (CH_2_, C-23), 24.52 (CH_3_, C-30), 21.49 (CH_2_, C-16), 20.76 (CH_3_, C-27), 20.67 (CH_3_, C-28), 20.24 (CH_2_, C-6), 18.90 (CH_3_, C-19), 18.80 (CH_3_, C-21), 15.78 (CH_3_, C-18). LC-ESI-HRMS (−) calcd. for C_31_H_45_O_4_^−^: *m*/*z* 481.3323, found 481.3299.

#### 3.2.2. Synthesis of (S,Z)-2-Methyl-6-((1S,3aS,5aR,10aS,12aS)-3a,6,6,10a,12a-pentamethyl-2,3,3a,4,5,5a,6,10,10a,11,12,12a-dodecahydro-1H-cyclopenta[7,8]phenanthro[3,2-d]isoxazol-1-yl)hept-2-enoic Acid (**3**)

To a solution of **2** (248.7 mg, 0.515 mmol) in abs EtOH (12.4 mL) and H_2_O (1.8 mL) NH_2_OH·HCl was added (10.0 eq) and the mixture was stirred at 80 °C for 2 h. Subsequently, the EtOH was evaporated under reduced pressure, and the residue was extracted with EtOAc and H_2_O. The organic phase was then dried over anhydrous Na_2_SO_4_ and concentrated. A solid residue of 210.0 mg was obtained, which was further purified by column chromatography (silica gel) using a c-hexane/EtOAc 95.5:4.5 *v*/*v*, as the eluent. Finally, 190.0 mg of **3** were obtained as a pale yellow solid (yield = 76%). ${\left[a\right]}_{D}^{25}$ +13 (c 0.560, CHCl_3_); ^1^H NMR (600 MHz, CDCl_3_) δ (ppm) 8.01 (1H, t, H-31), 6.09 (1H, t, H-24), 2.56 (1H, m, H-23a), 2.53 (1H, d, *J* = 14.87 Hz, H-1a), 2.45 (1H, m, H-23b), 2.19 (1H, d, *J* = 15.10 Hz, H-1b), 2.15 (2H, m, H-27), 2.12–2.06 (2H, m, H-16), 1.96 (2H, m, H-11), 1.92 (3H, s, CH_3_-27), 1.83–1.72 (2H, m, H-12), 1.64 (1H, dd, *J* = 1.5/11.63 Hz, H-5), 1.55 (1H, m, H-15a), 1.53 (2H, m, H-22), 1.53 (1H, m, H-17), 1.45 (2H, m, H-6), 1.44 (1H, m, H-20), 1.30 (3H, s, CH_3_-29), 1.25 (1H, m, H-15b), 1.22 (3H, s, CH_3_-28), 0.94 (3H, d, *J* = 6.37 Hz, CH_3_-21), 0.91 (3H, s, CH_3_-19), 0.90 (3H, s, CH_3_-30), 0.77 (3H, s, CH_3_-18); ^13^C NMR (151 MHz, CDCl_3_) δ (ppm) 173.68 (C, C-3), 172.79 (C, C-26), 150.50 (C, C-31), 147.50 (CH, C-24), 135.83 (C, C-8), 131.64 (C, C-9), 125.92 (C, C-25), 109.74 (C, C-2), 50.83 (CH, C-5), 50.39 (C, C-13), 50.25 (CH, C-17), 44.32 (C, C-14), 39.26 (C, C-10), 36.65 (CH, C-20), 36.06 (CH_2_, C-22), 35.13 (C, C-4), 32.33 (CH_2_, C-1), 30.90 (CH_2_, C-12), 30.00 (CH_2_, C-15), 29.12 (CH_3_, C-29), 28.24 (CH_2_, C-11), 27.88 (CH_2_,C-27), 27.09 (CH_2_, C-23), 24.47 (CH_3_, C-30), 21.54 (CH_2_, C-16), 21.18 (CH_3_, C-28), 20.79 (CH_3_, C-26), 19.78 (CH_3_, C-19), 19.72 (CH_2_, C-6), 18.80 (CH_3_, C-21), 15.79 (CH_3_, C-18). LC-ESI-HRMS (−) calcd. for C_31_H_44_ΝO_3_^−^: *m*/*z* 478.3327, found 478.3319.

#### 3.2.3. Synthesis of 2-Bromo-3-oxotirucalla-8,24Z-dien-26-oic Acid (**4**)

To a solution of **1** (210.0 mg, 0.462 mmol) in ACN (15.0 mL) under argon, PyHBr_3_ (1.5 eq) is added, and the mixture is stirred at room temperature for 12 h. Upon completion of the reaction, the solvent is removed under reduced pressure, and the resulting oily residue is dissolved in EtOAc, washed with H_2_O, dried over anhydrous Na_2_SO_4_, filtered, and concentrated under reduced pressure. A solid residue of 250.0 mg is obtained, which is purified by column chromatography (silica gel) using c-hexane/DCM: 5:95→100:0% *v*/*v*, as the eluent. Finally, a mixture of the 2-bromo-substituted IMNA isomers is obtained (164.0 mg) as a yellow solid (yield = 66%). ${\left[a\right]}_{D}^{25}$ −12 (c 0.559, CHCl_3_); LC-ESI-HRMS (−) calcd for C_30_H_44_BrO_3_^−^: *m*/*z* 531.2479, found 531.2479.

#### 3.2.4. Synthesis of (S,Z)-6-((1S,3aS,5aR,10aS,12aS)-8-Amino-3a,6,6,10a,12a-pentamethyl-2,3,3a,4,5,5a,6,10,10a,11,12,12a-dodecahydro-1H-cyclopenta[7,8]phenanthro[2,3-d]thiazol-1-yl)-2-methylhept-2-enoic Acid (**5**)

To a solution of **4** (52.0 mg, 0.097 mmol) in absolute EtOH, 10.0 eq. of thiourea were added. The resulting mixture was stirred at room temperature for 5 days under inert atmosphere. After completion of the reaction and the evaporation of the EtOH, the residue was dissolved in EtOAc, washed with H_2_O, dried over anhydrous Na_2_SO_4_ and concentrated under reduced pressure. The crude product was purified by flash chromatography (silica gel), using DCM/MeOH, 100:0→98:2 *v/v* as the eluent, to afford 40.0 mg of **5** (yield: 80%). ${\left[a\right]}_{D}^{25}$ +32 (c 0.357, CH_3_OH); ^1^H NMR (600 MHz, CD_3_OD) δ (ppm) 5.93 (1H, dt, *J* = 1.29/7.72 Hz, H-24), 2.62 (1H, d, *J* = 15.19, H-1a), 2.50 (1H, m, H-23a), 2.39 (1H, m, H-23b), 2.31 (1H, d, *J* = 15.48 Hz, H-1b), 2.19 (1H, dd, *J* = 5.10/17.45 H-7a), 2.10 (2H, m, H-11), 1.99 (1H, m, H-7b), 1.87 (3H, s, CH_3_-27), 1.81 (1H, m, H-12a), 1.76 (1H, m, H-12b), 1.75 (1H, m, H-6a), 1.62 (1H, m, H-16a), 1.59 (1H, m, H-6b), 1.58 (1H, m, H-17), 1.54 (1H, m, H-22a), 1.46 (1H, m, H-5), 1.46 (1H, m, H-20), 1.37 (2H, m, H-15), 1.28 (1H, m, H-16b), 1.21 (3H, s, CH_3_-29), 1.17 (1H, m, H-22b), 1.14 (3H, s, CH_3_-28), 1.01 (3H, s, CH_3_-19), 0.96 (3H, d, *J* = 6.50 Hz, CH_3_-21), 0.94 (3H, s, CH_3_-30), 0.82 (3H, s, CH_3_-18); ^13^C NMR (151 MHz, CD_3_OD) δ (ppm) 171.94 (C, C-26), 169.52 (C, C-31), 149.91 (C, C-3), 143.83 (CH, C-24), 137.17 (C, C-8), 132.89 (C, C-9), 128.69 (C, C-25), 115.92 (C, C-2), 51.37 (CH, C-17), 51.35 (CH, C-5), 51.16 (C, C-13), 45.31 (C, C-14), 39.90 (C, C-10), 37.72 (CH, C-20), 37.65 (C, C-4), 37.00 (CH_2_, C-22), 36. 17 (CH_2_, C-1), 31.97 (CH_2_, C-12), 30.38 (CH_3_, C-29), 29.02 (CH_2_, C-15), 28.84 (CH_2_, C-7), 27.49 (CH_2_, C-23), 24.65 (CH_3_, C-30), 22.21 (CH_2_, C-11), 21.74 (CH_3_, C-28), 21.18 (CH_2_, C-6), 21.04 (CH_3_, C-27), 19.92 (CH_3_, C-19), 19.12 (CH_3_, C-21), 19.06 (CH_3_, C-18). LC-ESI-HRMS (−) calcd. for C_31_H_45_N_2_O_2_S^−^: *m*/*z* 509.3207, found 509.3193.

#### 3.2.5. Synthesis of 2-Thiocyanate-3-oxotirucalla-8,24Z-dien-26-oic Acid (**6**)

To a solution of **4** (122.8 mg, 0.230 mmol) in 2.0 mL DMSO, KSCN (156 mg, 1.61 mmol, 7.0 eq) were added. The resulting mixture was stirred at 90 °C for 4 h under inert atmosphere. After completion of the reaction the residue was dissolved in EtOAc, washed with H_2_O, dried over anhydrous Na_2_SO_4_, and concentrated under reduced pressure. The crude product was purified by flash chromatography (silica gel), using c-hexane/DCM: 5:95→100:0% *v*/*v*, as the eluent, to afford 105.0 mg of **6** (yield: 89%). ${\left[a\right]}_{D}^{25}$ −80 (c 0.320, CHCl_3_); LC-ESI-HRMS (−) calcd. for C_31_H_44_NO_3_S^−^: *m*/*z* 510.3047, found 510.3027.

#### 3.2.6. Synthesis of (S,Z)-2-Methyl-6-((1S,3aS,5aR,10aS,12aS)-3a,6,6,10a,12a-pentamethyl-8-morpholino-2,3,3a,4,5,5a,6,10,10a,11,12,12a-dodecahydro-1H-cyclopenta[7,8]phenanthro[2,3-d]thiazol-1-yl)hept-2-enoic Acid (**7**)

To a solution of **6** (83.0 mg, 0.162 mmol) in CHCl_3_, 5.0 eq. of freshly prepared Morpholinium acetate were added. The resulting mixture was stirred at room temperature for 7 days. After completion of the reaction the residue was dissolved in DCM, washed with H_2_O, dried over anhydrous Na_2_SO_4_ and concentrated under reduced pressure. The crude product was purified by flash chromatography (silica gel), using c-hexane/DCM: 20:80→100:0% *v/v* as the eluent, to afford 40.0 mg of **3** (yield: 40%). ${\left[a\right]}_{D}^{25}$ +13 (c 0.701, CHCl_3_); ^1^H NMR (600 MHz, CDCl_3_) δ (ppm) 6.08 (1H, dt, *J* = 1.08/7.55 Hz, H-24), 3.79 (4H, m, H-33, H-34), 3.40 (4H, m, H-32, H-35), 2.64 (1H, d, *J* = 15.25 Hz, H-2a), 2.57 (1H, m, H-23a), 2.47 (1H, m, H-23b), 2.37 (1H, d, *J* = 15.06 Hz, H-2b), 2.14 (1H, m, H-7a), 2.07 (2H, m, H-16), 1.98 (1H, m, H-7b), 1.92 (3H, s, CH_3_-27), 1.76 (2H, m, H-12), 1.74 (1H, m, H-6a), 1.58 (2H, m, H-11), 1.58 (1H, m, H-5), 1.54 (1H, m, H-22a), 1.51 (1H, m, H-17), 1.44 (1H, m, H-6b), 1.44 (1H, m, H-20), 1.35 (2H, m, H-15), 1.22 (3H, s, CH_3_-29), 1.15 (4H, m, H-22b), 1.14 (3H, s, CH_3_-28), 0.95 (3H, s, CH_3_-19), 0.94 (3H, d, *J* = 6.28 Hz, CH_3_-21), 0.90 (3H, s, CH_3_-30), 0.77 (3H, s, CH_3_-18); ^13^C NMR (151 MHz, CDCl_3_) δ (ppm) 172.15 (C, C-26), 168.76 (C, C-31), 153.93 (C, C-3), 147.40 (CH, C-24), 135.8 (C, C-8), 132.01 (C, C-9), 125.83 (C, C-25), 115.72 (C, C-2), 66.53 (CH_2_, C-33 and C-34), 50.43 (CH, C-17), 50.32 (CH, C-13), 49.94 (CH, C-5), 48.84 (CH_2_, C-32 and C-33), 44.40 (C, C-14), 38.92 (C, C-10), 37.57 (C, C-4), 36.69 (CH, C-20), 36.09 (CH_2_, C-22), 35.70 (CH_2_, C-1), 31.00 (CH_2_, C-12), 30.71 (CH_3_-29), 30.01 (CH_2_-11), 28.28 (CH_2_, C-15), 28.00 (CH_2_, C-7), 27.11 (CH_2_-23), 24.46 (CH_3_,C-30), 21.96 (CH_3_, C-28), 21.39 (CH_2_, C-16), 20.79 (CH_3_, C-27), 20.50 (CH2, C-6), 19.80 (CH_3_, C-19), 18.82 (CH_3_, C-21), 15.82 (CH_3_, C-18). LC-ESI-HRMS (−) calcd for C_35_H_51_N_2_O_3_S^−^: *m*/*z* 579.3626, found 579.3600.

#### 3.2.7. Synthesis of 2-Hydroxy-3-oxotirucalla-8,24Z-dien-26-oic acid (**8**), 1α-hydroxy-3-oxa-nor- tirucalla-8,24Z-dien-27-oic acid (**9**), 3β-hydroxy-1(2→3)-abeotirucalla-8,24Z-dien-26-oic Acid (**10**)

To a solution of **1** (200.0 mg, 0.439 mmol) in *t*-BuOH, *t*-BuOK (359 mg, 3.2 mmol, 7.3 eq) was added under continuous oxygen flow and the resulting mixture was stirred at 40 °C for 2 h. Then, KOH (123.0 mg, 2.2 mmol, 5.0 eq) in 1.4 mL of H_2_O were added, and the mixture was stirred at 75 °C for 24 h. After completion of the reaction, H_2_O and 6N HCl were added until the pH reached 4. The mixture was extracted with EtOAc, the organic phase was washed with H_2_O (×5), dried over anhydrous Na_2_SO_4_, and concentrated to dryness. This afforded 190.0 mg of an oily residue, which was purified by column chromatography (silica gel) using a DCM/MeOH. Analog **8** eluted with 1% MeOH (20.0 mg, yield = 10%), **9** with 3% MeOH (40 mg, yield = 21%), **10** with 5–10% MeOH (60 mg, yield = 30%). %). NMR data of compounds **8** and **10** were consistent with the literature [23].

#### 3.2.8. Spectroscopic Data of 1α-Hydroxy-3-oxa-nor-oxotirucalla-8,24Z-dien-27-oic acid (**9**)

${\left[a\right]}_{D}^{25}$ +2 (c 0.327, CH_3_OH); ^1^H NMR (600 MHz, CD_3_OD) δ (ppm) 5.95 (1H, t, *J* = 7.8/15.1 Hz H-24), 5.49 (1H, s, H-1), 2.51 (1H, m, H-23a), 2.38 (1H, m, H-5), 2.38 (1H, m, H-23b), 2.16 (2H, m, H-7), 2.12 (1H, m, H-11a), 2.09 (1H, m, H-11b), 1.99 (1H, m, H-16a), 1.87 (3H, s, CH_3_-27), 1.80 (1H, m, H-12a), 1.74 (1H, m, H-12b), 1.67 (1H, m, H-6a), 1.61 (2H, m, H-15), 1.61 (1H, m, H-6b), 1.55 (1H, m, H-17), 1.53 (1H, m, H-22a), 1.45 (1H, m, H-20), 1.37 (1H, m, H-16b), 1.26 (3H, s, CH_3_-29), 1.21 (3H, s, CH_3_-30), 1.16 (1H, m, H-22b), 1.06 (3H, s, CH_3_-19), 0.94 (3H, d, *J* = 6.3 Hz, CH_3_-21), 0.92 (3H, s, CH_3_-28), 0.82 (3H, s, CH_3_-18); ^13^C NMR (151 MHz, CD_3_OD) δ (ppm) 181.4 (C, C-3), 171.6 (C, C-26), 144.1 (CH, C-24), 138.3 (C, C-9), 129.8 (C, C-8), 128.4 (C, C-25), 102.2 (CH, C-1), 51.3 (CH, C-17), 51.2 (C, C-13), 45.5 (C, C-14), 41.7 (C, C-10), 41.5 (C, C-4), 40.4 (CH, C-5), 37.6 (CH, C-20), 37.0 (CH_2_, C-22), 31.7 (CH_2_, C-12), 30.7 (CH_2_, C-15), 29.5 (CH_3_, C-29), 28.9 (CH_2_, C-16), 28.0 (CH_2_, C-7), 27.4 (CH_2_, C-23), 24.6 (CH_3_, C-28), 23.6 (CH_3_, C-30), 21.8 (CH_2_, C-11), 21.0 (CH_3_, C-27), 20.0 (CH_2_, C-6), 19.1 (CH_3_, C-21), 18.7 (CH_3_, C-19), 15.7 (CH_3_, C-18). LC-ESI-HRMS (−) calcd. for C_29_H_43_O_5_^−^: *m*/*z* 471.3116, found 471.3152.

#### 3.2.9. Synthesis of 3-One-1(2→3)-abeotirucalla-8,24*Z*-dien-26-oic Acid (**11**)

To a solution of **10** (168.8 mg, 0.347 mmol) in AcOH (6.0 mL) Pb(C_2_H_3_O_2_)_4_ (309.6 mg, 0.694 mmol, 2.0 eq) was added, and the resulting mixture was stirred at room temperature for 5 h. After completion of the reaction, AcOH was evaporated under reduced pressure, the residue was redissolved in EtOAc and washed with H_2_O (x5). After drying the organic phase over anhydrous Na_2_SO_4_ and concentrating under reduced pressure, 191.6 mg of solid residue was obtained. This was purified by column chromatography (silica gel) using c-hexane/EtOAc: 96:4 → 92:8 *v*/*v*, as the eluent. Finally, 95.0 mg of **11** was obtained as a pale yellow solid (yield = 62%). ${\left[a\right]}_{D}^{25}$ +30 (c 0.322, CHCl_3_); ^1^H NMR (600 MHz, CDCl_3_) δ (ppm) 6.08 (1H, dt, *J* = 1.40/7.49 Hz, H-24), 2.57 (1H, m, H-22a), 2.46 (2H, m, H-22b), 2.21 (2H, m, H-1), 2.17 (1H, m, H-15a), 2.11 (1H, m, H-15b), 2.06 (1H, m, H-6a), 1.97 (1H, m, H-6b), 1.95 (1H, m, H-10a), 1.94 (1H, dd, *J* = 3.70/3.53 Hz, H-4), 1.91 (3H, s, CH_3_-25), 1.72 (2H, m, H-11), 1.67 (2H, m, H-5), 1.56 (1H, m, H-14a), 1.50 (1H, m, H-16), 1.50 (1H, m, H-16), 1.43 (1H, m, H-19), 1.31 (2H, m, H-10b), 1.23 (1H, m, H-14b), 1.14 (1H, m, H-21b), 1.03 (3H, s, CH_3_-28), 1.00 (3H, s, CH_3_-27), 0.939 (3H, d, *J* = 6.28 Hz, CH_3_-21), 0.933 (3H, s, CH_3_-18), 0.89 (3H, s, CH_3_-29), 0.78 (3H, s, CH_3_-17); ^13^C NMR (151 MHz, CDCl_3_) δ (ppm) 224.59 (C, C-2), 173.24 (C, C-26), 147.49 (CH, C-23), 134.58 (C, C-7), 132.93 (C, C-8), 125.99 (C, C-24), 54.05 (CH, C-4), 50.57 (CH2, C-1), 50.28 (CH, C-16), 49.81 (C, C-12), 46.06 (C, C-33), 44.87 (C, C-13), 40.76 (C, C-9), 36.63 (CH, C-19), 36.08 (CH_2_, C-21), 30.60 (CH_2_, C-11), 29.96 (CH_2_, C-14), 28.22 (CH_2_, C-10), 28.04 (CH_3_-28), 27.06 (CH_2_-22), 26.13 (CH_2_, C-15), 24.65 (CH_3_, C-29), 23.29 (CH_2_-6), 22.01 (CH_3_,C-18), 21.16 (CH_3_, C-27), 20.75 (CH_3_, C-25), 18.83 (CH_3_, C-20), 18.35 (CH_2_, C-5), 15.82 (CH_3_, C-17). LC-ESI-HRMS (−) calcd. for C_29_H_43_O_3_^−^: *m*/*z* 439.3218, found 439.3212.

### 3.3. Antitumoral Assays

Human cancer cell lines used in this study included Capan-1, HCT-116, NCI-H460, LN-229, HL-60, K-562, and Z-138, all obtained from the American Type Culture Collection (ATCC). The DND-41 cell line was sourced from the Deutsche Sammlung von Mikroorganismen und Zellkulturen (DSMZ). All cell lines were cultured in media obtained from Gibco Life Technologies, supplemented with 10% fetal bovine serum (HyClone, Logan, UT, USA). Adherent cell lines were seeded in 384-well tissue culture plates at the following densities: Capan-1 at 500 cells/well, and HCT-116, NCI-H460, and LN-229 at 1500 cells/well. After overnight incubation to allow cell attachment, cells were treated with a seven-point serial dilution of test compounds, ranging from 100 µM to 0.006 µM. Suspension cell lines were seeded as follows: HL-60, K-562, and Z-138 at 2500 cells/well, and DND-41 at 5500 cells/well, using the same compound concentration range. All treatments were performed in 384-well plates under identical conditions. Following 72 h of compound exposure, cell viability was assessed using the CellTiter 96^®^ AQueous One Solution Cell Proliferation Assay (MTS, Promega, Leiden, The Netherlands), following the manufacturer’s protocol. The final assay mixture contained 333 µg/mL MTS and 25 µM phenazine methosulfate (PMS). Absorbance was measured at 490 nm using a SpectraMax Plus 384 microplate reader (Molecular Devices, San Jose, CA, USA). Optical density values were used to calculate the half-maximal inhibitory concentration (IC_50_) for each compound. All compounds were tested in a minimum of two independent biological replicates to ensure reproducibility.

### 3.4. Cell Viability of PBMCs

A buffy coat preparation sourced from a healthy donor was acquired from the Blood Transfusion Center in Leuven, Belgium. Peripheral blood mononuclear cells (PBMCs) were separated using density gradient centrifugation over Lymphoprep (density: 1.077 g/mL) from Nycomed. These cells were then cultured in cell culture medium (RPMI, Gibco, Waltham, MA, USA) supplemented with 10% FBS. The PBMCs were seeded at a density of 28,000 cells per well in 384-well tissue culture plates containing the test compounds at concentrations ranging from 50 µM to 3.2 nM (for compounds **3**–**11** and IMNA) and from 10 µM to 0.6 nM (for etoposide). Following a 72 h incubation period, cell viability was assessed using the MTS cell viability assay. Each compound was evaluated in duplicate.

## 4. Conclusions

In this study, a focused library of 11 IMNA analogs was synthesized through targeted A-ring modifications, including the introduction of heteroatoms, heterocyclic substitutions, and structural rearrangements. These modifications were designed to explore the impact of A-ring structural changes on anticancer activity, while preserving key pharmacophoric elements of the IMNA scaffold. The newly synthesized compounds were evaluated for their antiproliferative effects across a panel of solid and hematological cancer cell lines. Among the solid tumors, the pancreatic adenocarcinoma cell line (Capan-1) emerged as the most sensitive, with all compounds showing cytotoxicity in the 6–39 µM range. Compounds **3**, **7**, and **9** displayed selective activity toward Capan-1, whereas compounds **6** and **10** exhibited broad-spectrum antiproliferative effects across all tested cell lines. IMNA itself, along with several other derivatives, showed intermediate activity, while compounds **8** and **11** had a more restricted cytotoxic profile. These findings underscore IMNA as a promising scaffold and illustrate that rational A-ring modifications can meaningfully enhance both activity and selectivity in triterpenoid-based anticancer agents. These results are based on initial in vitro studies, and they provide a solid foundation for further research, which will focus on optimizing pharmacological properties, expanding structural modifications beyond the A-ring, and integrating computational and mechanistic approaches to elucidate the mode of action and to guide the design of more potent and selective analogs.

## Figures and Tables

**Table 1:** Cytotoxicity (expressed as IC_50_ values) of IMNA and its derivatives against a panel of human cancer cell lines. Results (in µM) are presented as the mean ± standard deviation of two independent experiments.

Compound	Capan-1	HCT-116	LN229	NCl-H460	DND-41	HL-60	K562	Z138
**3**	6.7 ± 0.4	>50	>50	>50	>50	>50	>50	>50
**4**	9.4 ± 1.5	22.0 ± 0.8	28.5 ± 5.3	25.1 ± 1.9	20.5 ± 3.9	14.7 ± 3.3	>50	>50
**5**	23.5 ± 2.4	28.2 ± 1.9	27.9 ± 2.9	24.5 ± 7.2	19.0 ± 2.0	19.5 ± 3.2	>50	25.0 ± 1.4
**6**	7.4 ± 1.0	24.3 ± 1.4	23.3 ± 2.9	21.3 ± 2.3	18.5 ± 0.7	15.0 ± 1.2	35.0 ± 3.9	17.9 ± 0.4
**7**	26.0 ± 0.4	>50	>50	>50	>50	>50	>50	>50
**8**	22.1 ± 2.6	>50	>50	>50	21.6 ± 1.6	>50	>50	>50
**9**	39.4 ± 1.5	>50	>50	>50	>50	>50	>50	>50
**10**	9.9 ± 3.6	24.6 ± 1.2	26.8 ± 2.3	22.1 ± 0.9	19.5 ± 1.9	14.2 ± 1.4	23.7 ± 2.5	28.8 ± 0.6
**11**	24.6 ± 2.1	>50	>50	>50	49.3 ± 0.8	29.6 ± 8.9	>50	>50
IMNA	26.5 ± 1.6	42.3 ± 2.3	>50	>50	34.2 ± 2.5	21.7 ± 3.1	>50	32.7 ± 3.2
Etoposide	0.24 ± 0.01	1.09 ± 0.19	4.80 ± 0.87	2.36 ± 0.70	0.08 ± 0.03	0.30 ± 0.12	4.47 ± 0.11	0.08 ± 0.007

## Data Availability

Data are contained within the article and Supplementary Materials.

## Supplementary Materials

The following supporting information can be downloaded at: https://www.mdpi.com/article/10.3390/molecules30234572/s1, Figures S1–S47: 1H and 13C-NMR and 2D NMR spectra are available online.
