# Supplementary material for: Design and Synthesis of Structurally Modified Analogs of 24Z-Isomasticadienonic Acid with Enhanced Anti-Proliferative Activity
Source: Molecules. 2025 Nov 27;30(23):4572. doi: 10.3390/molecules30234572 (PMC12692795; doi:10.3390/molecules30234572)
Supplement: Supplementary file 1 [file molecules-30-04572-s001.zip › molecules-3959980-supplementary.pdf]

Supplementary materials

Design and Synthesis of Structurally Modified Analogs of 24*Z*-Isomasticadienonic Acid with Enhanced Anti-Proliferative Activity

Panagiota Stamou [1], Leentje Persoons [2], Dominique Schols [3], Steven De Jonghe [3], Leandros A. Skaltsounis [4] and Ioannis K. Kostakis [1,*]

[1] Department of Pharmacy, Division of Pharmaceutical Chemistry, National and Kapodistrian University of Athens, Panepistimiopolis Zografou, 15771 Athens, Greece; pstamou@pharm.uoa.gr

[2] Molecular Genetics and Therapeutics in Virology and Oncology Research Group, Department of Microbiology, Immunology and Transplantation, Rega Institute for Medical Research, KU Leuven, Herestraat 49, Box 1048, 3000 Leuven, Belgium; leentje.persoons@kuleuven.be

[3] Molecular, Structural and Translational Virology Research Group, Department of Microbiology, Immunology and Transplantation, Rega Institute for Medical Research, KU Leuven, Herestraat 49, Box 1049, 3000 Leuven, Belgium; dominique.schols@kuleuven.be (D.S.); steven.dejonghe@kuleuven.be (S.D.J.)

[4] Department of Pharmacy, Division of Pharmacognosy and Natural Products Chemistry, National and Kapodistrian University of Athens, Panepistimiopolis Zografou, 15771 Athens, Greece; skaltsounis@pharm.uoa.gr

*Correspondence: ikkostakis@pharm.uoa.gr; Tel.: +210-727212

Contents

Figure S1: ^{1}H (400 MHz, $CDCl_3$) spectrum of **1**....4

Figure S2: ^{13}C (100 MHz, $CDCl_3$) spectrum of **1**....4

Figure S3: (A) ^{1}H (600 MHz, $CDCl_3$) spectrum of **2**. (B) Zoom region from 0.7 to 2.68 ppm....5

Figure S4: COSY (600 MHz, $CDCl_3$) spectrum of **2**....6

Figure S5: HSQC (600 MHz, $CDCl_3$) spectrum of **2**. (B) Zoom region from 0.7 to 2.68 ppm....7

Figure S6: HMBC (600 MHz, $CDCl_3$) spectrum of **2**....8

Figure S7: (A) ^{13}C (100 MHz, $CDCl_3$) spectrum of **2**. (B) Zoom region from 10.00 to 43.00 ppm. 9

Figure S8: (A) ^{1}H (600 MHz, $CDCl_3$) spectrum of **3**. (B) Zoom region from 0.70 to 2.68 ppm.. 10

Figure S9: COSY (600 MHz, $CDCl_3$) spectrum of **3**.... 11

Figure S10: HSQC (600 MHz, $CDCl_3$) spectrum of **3**. (B) Zoom region from 0.70 to 2.68 ppm.12

Figure S11: HMBC (600 MHz, $CDCl_3$) spectrum of **3**.... 13

Figure S12: (A) ^{13}C (150 MHz, $CDCl_3$) spectrum of **3**. (B) Zoom region from 10.00 to 52.00 ppm. 14

Figure S13: ^{1}H (400 MHz, $CDCl_3$) spectrum of **4**.... 15

Figure S14: COSY (400 MHz, $CDCl_3$) spectrum of **4**.... 16

Figure S15: HSQC (400 MHz, $CDCl_3$) spectrum of **4**.... 17

Figure S16: HMBC (400 MHz, $CDCl_3$) spectrum of **4**.... 18

Figure S17: ^{13}C (100 MHz, $CDCl_3$) spectrum of **4**.... 19

Figure S18: ^{1}H (600 MHz, MeOD) spectrum of **5**. (B) Zoom region from 0.70 to 2.65 ppm..... 20

Figure S19: COSY (600 MHz, MeOD) spectrum of **5**.... 21

Figure S20: HSQC (600 MHz, MeOD) spectrum of **5**. (B) Zoom region from 0.70 to 2.80 ppm. 22

Figure S21: HMBC (600 MHz, MeOD) spectrum of **5**.... 23

Figure S22: ^{13}C (100 MHz, MeOD) spectrum of **5**. (B) Zoom region from 16.00 to 46.00 ppm. 24

Figure S23: ^{1}H (400 MHz, $CDCl_3$) spectrum of **6**. (B) Zoom region from 0.70 to 2.88 ppm..... 25

Figure S24: COSY (400 MHz, $CDCl_3$) spectrum of **6**.... 26

Figure S25: HSQC (400 MHz, $CDCl_3$) spectrum of **6**.... 27

Figure S26: HMBC (400 MHz, $CDCl_3$) spectrum of **6**.... 28

Figure S27: ^{13}C (100 MHz, $CDCl_3$) spectrum of **6**.... 29

Figure S28: ^{1}H (400 MHz, $CDCl_3$) spectrum of **7**. (B) Zoom region from 0.60 to 7.50 ppm..... 30

Figure S29: COSY (400 MHz, $CDCl_3$) spectrum of **7**.... 31

Figure S30: HSQC (400 MHz, $CDCl_3$) spectrum of **7**. (B) Zoom region from 0.70 to 2.80 ppm.32

Figure S31: HMBC (400 MHz, $CDCl_3$) spectrum of **7**.... 33

Figure S32: ^{13}C (100 MHz, $CDCl_3$) spectrum of **7**. (B) Zoom region from 15.00 to 69.00 ppm.. 34

Figure S33: ^{1}H (600 MHz, $CDCl_3$) spectrum of **8**.... 35

Figure S34: ^{13}C (150 MHz, $CDCl_3$) spectrum of **8**.. 35
Figure S35: ^{1}H (600 MHz, MeOD) spectrum of **9**. (B) Zoom region from 0.80 to 2.60 ppm. 36
Figure S36: COSY (600 MHz, MeOD) spectrum of **9**.. 37
Figure S37: HSQC (600 MHz, MeOD) spectrum of **9**. (B) Zoom region from 0.70 to 2.65 ppm. 38
Figure S38: HMBC (600 MHz, MeOD) spectrum of **9**. 39
Figure S39: ^{13}C (150 MHz, MeOD) spectrum of **9**. (B) Zoom region from 14.00 to 47.00 ppm. 40
Figure S40: NOESY (600 MHz, MeOD) spectrum of **9**.. 41
Figure S41: ^{1}H (600 MHz, MeOD) spectrum of **10**... 42
Figure S42: ^{13}C (150 MHz, MeOD) spectrum of **10**... 42
Figure S43: (A) ^{1}H (400 MHz, $CDCl_3$) spectrum of **11**. (B) Zoom region from 0.70 to 2.70 ppm. 43
Figure S44: COSY (400 MHz, $CDCl_3$) spectrum of **11**.. 44
Figure S45: (A) HSQC (400 MHz, $CDCl_3$) spectrum of **11**. (B) Zoom region from 0.60 to 2.80 ppm. 45
Figure S46: HMBC (400 MHz, $CDCl_3$) spectrum of **11**.. 46
Figure S47: (A) ^{13}C (100 MHz, $CDCl_3$) spectrum of **11**. (B) Zoom region from 14.00 to 56.00 ppm. 47

24*Z*-isomasticadienonic acid (1)

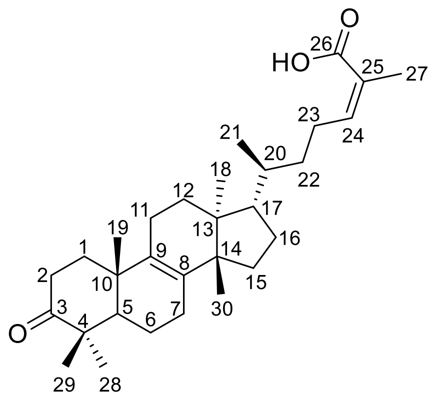

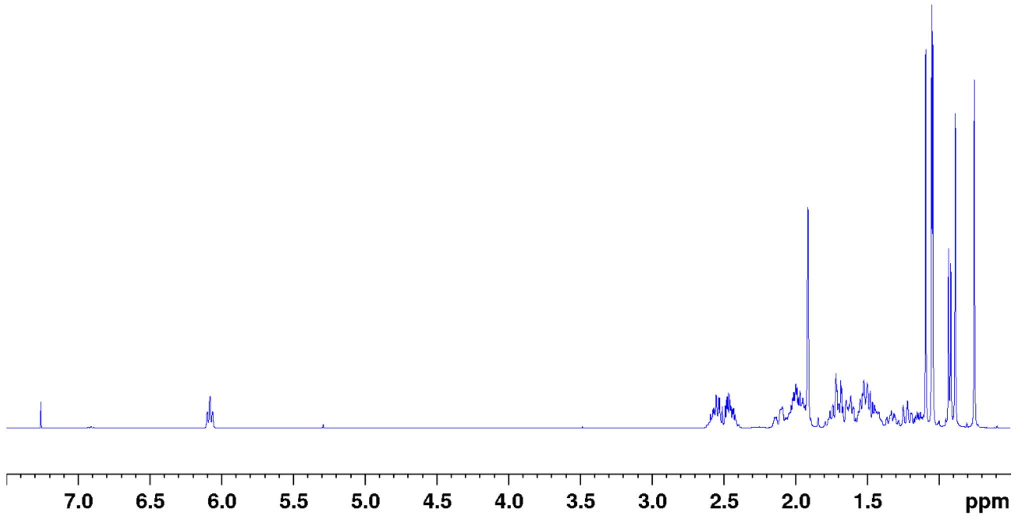

Figure S1: ¹H (400 MHz, $CDCl_3$) spectrum of **1**.

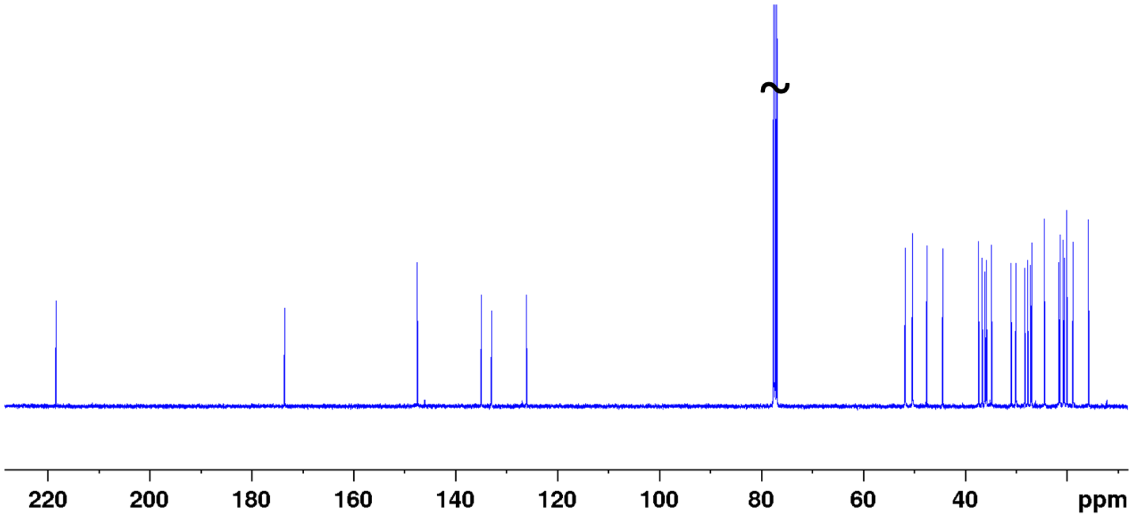

Figure S2: ¹³C (100 MHz, $CDCl_3$) spectrum of **1**.

2-hydroxymethylen-3-oxotirucalla-8,24*Z*-dien-26-oic acid (2)

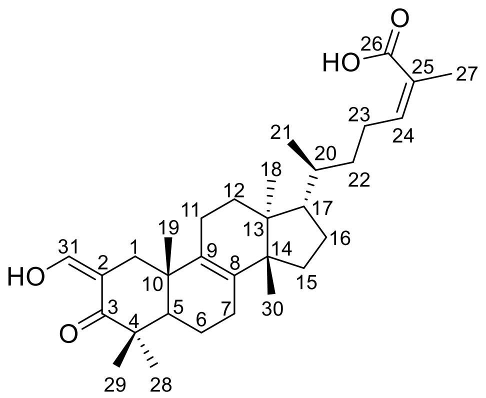

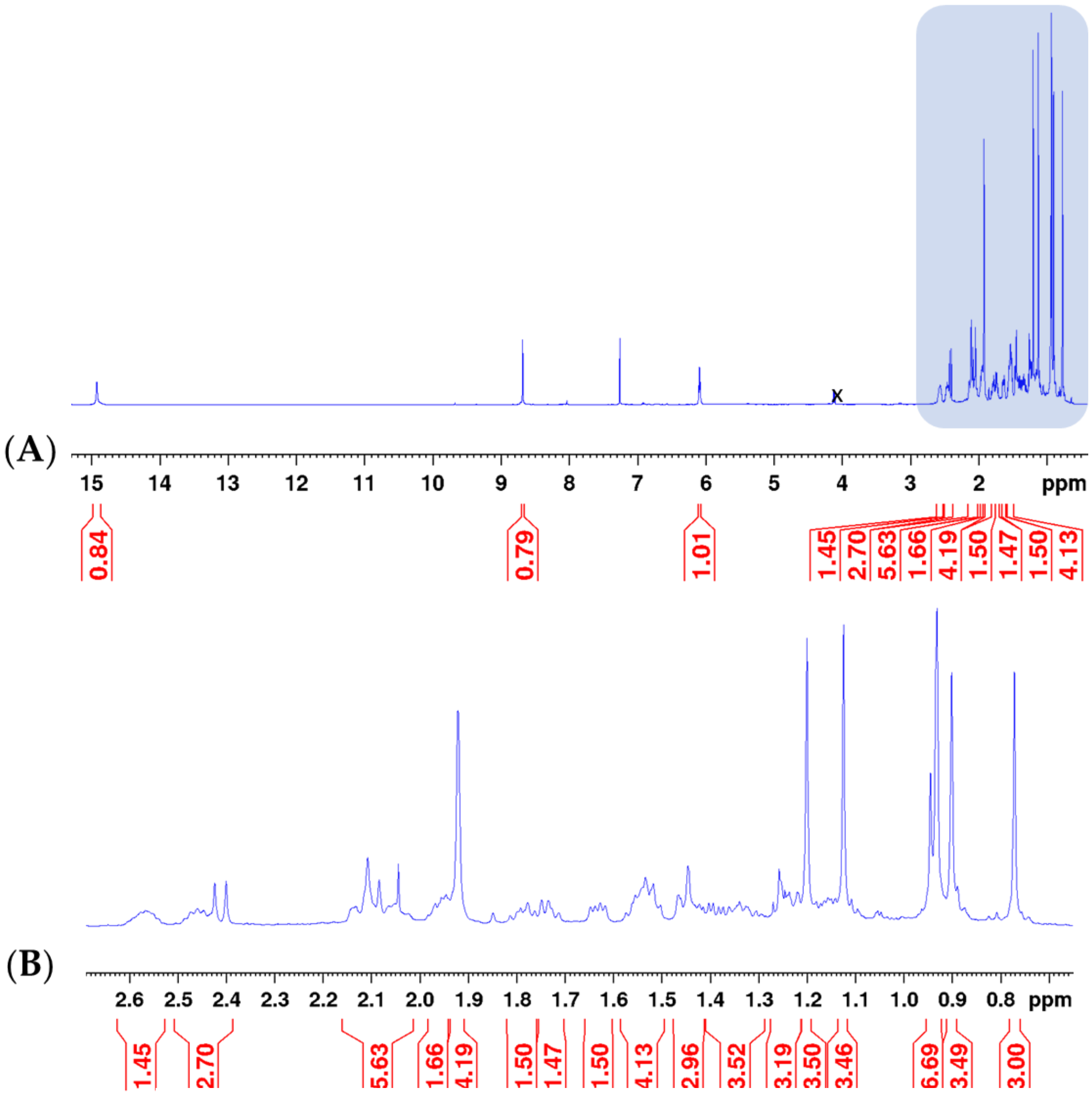

Figure S3: (A) ^{1}H (600 MHz, $CDCl_3$) spectrum of **2**. (B) Zoom region from 0.7 to 2.68 ppm.

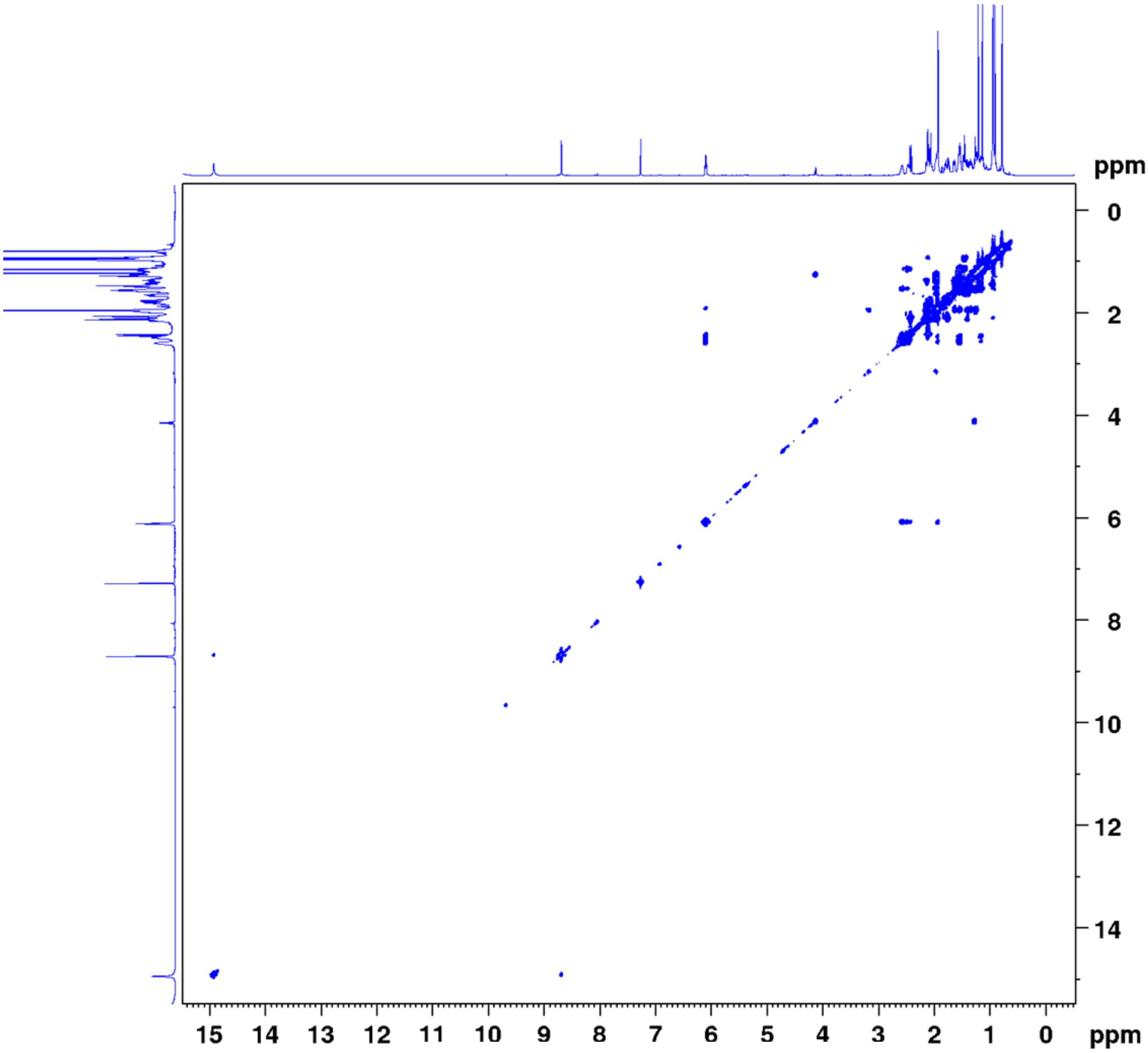

Figure S4: COSY (600 MHz, $CDCl_3$) spectrum of **2**.

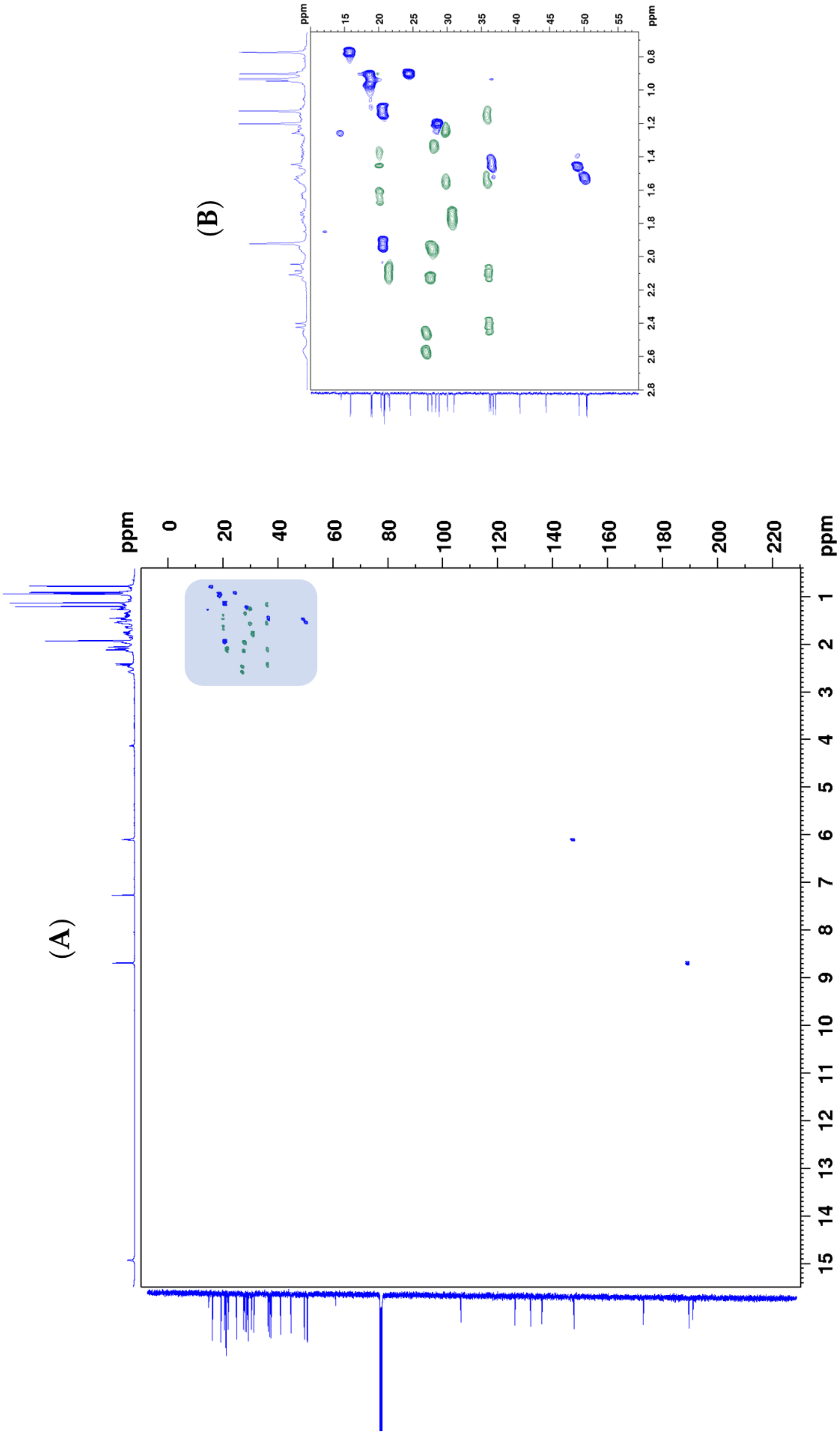

Figure S5: (A) HSQC (600 MHz, $CDCl_3$) spectrum of **2**. (B) Zoom region from 0.7 to 2.68 ppm.

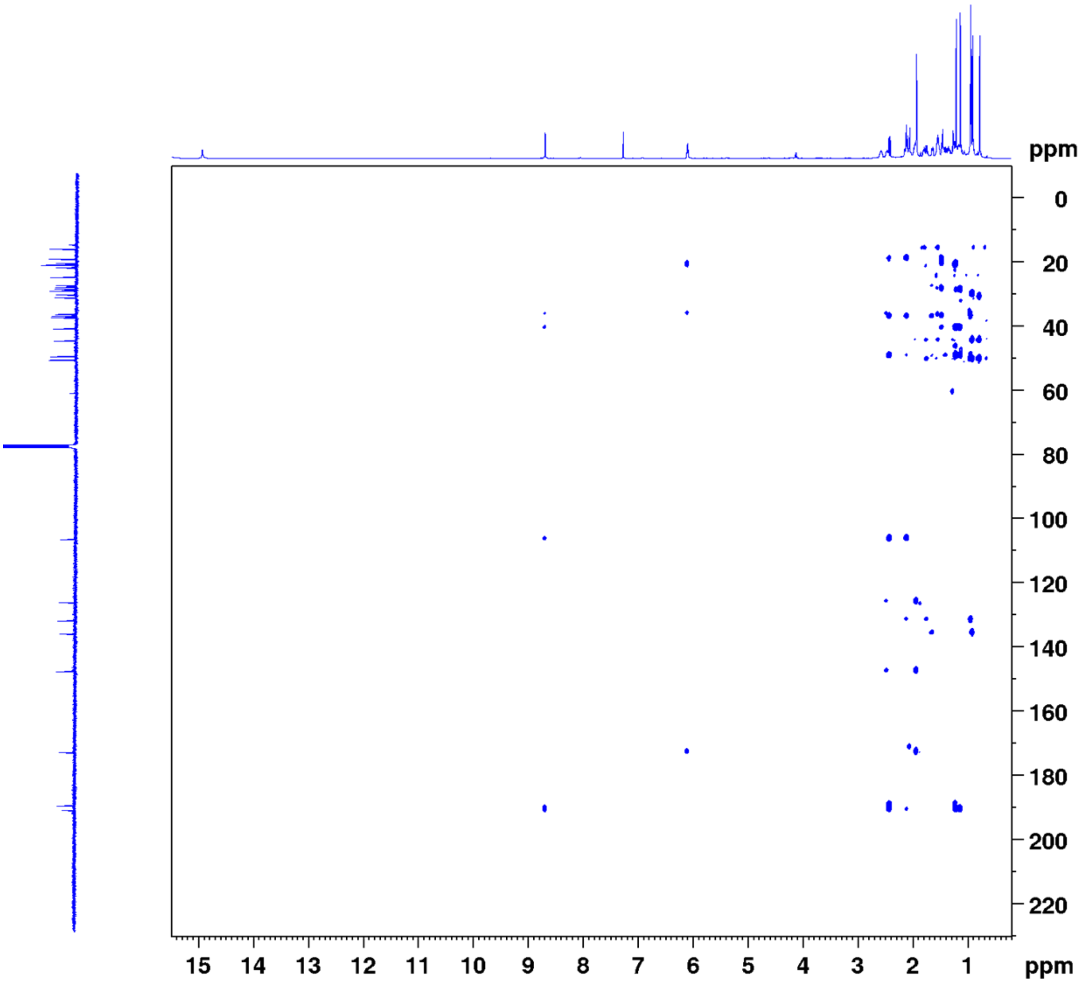

Figure S6: HMBC (600 MHz, $CDCl_3$) spectrum of **2**.

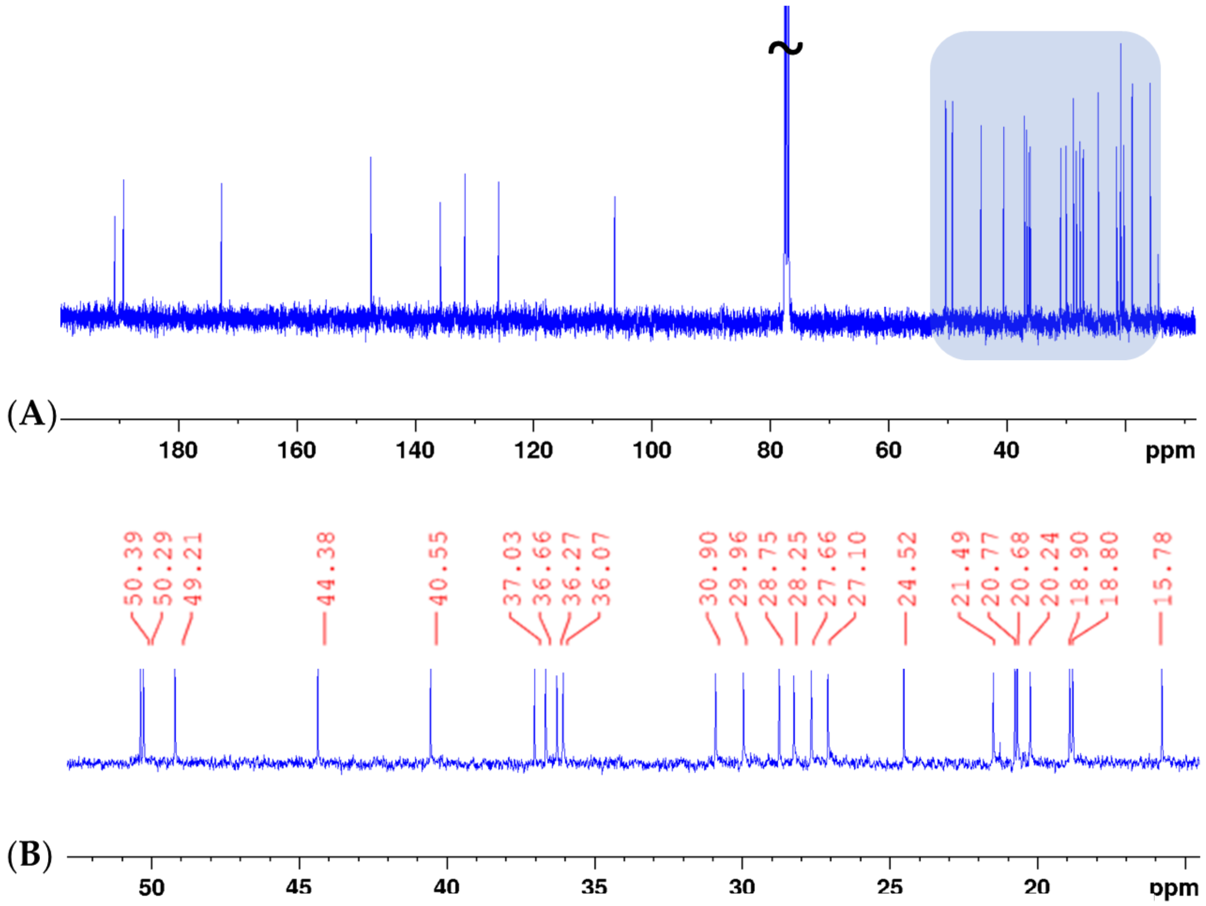

Figure S7: (A) ^{13}C (100 MHz, $CDCl_3$) spectrum of **2**. (B) Zoom region from 10.00 to 43.00 ppm.

(S,Z)-2-methyl-6-((1S,3aS,5aR,10aS,12aS)-3a,6,6,10a,12a-pentamethyl 2,3,3a,4,5,5a,6,10,10a,11,12,12a-dodecahydro-1H-cyclopenta[7,8]phenanthro[3,2-d]isoxazol-1-yl)hept-2-enoic acid (3)

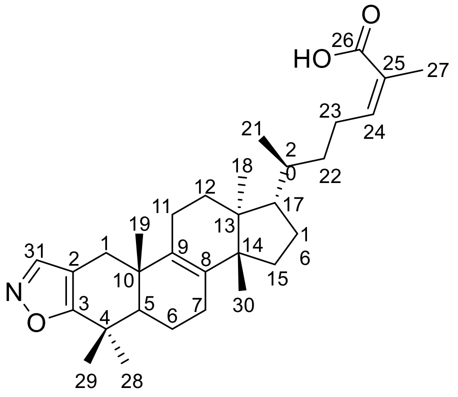

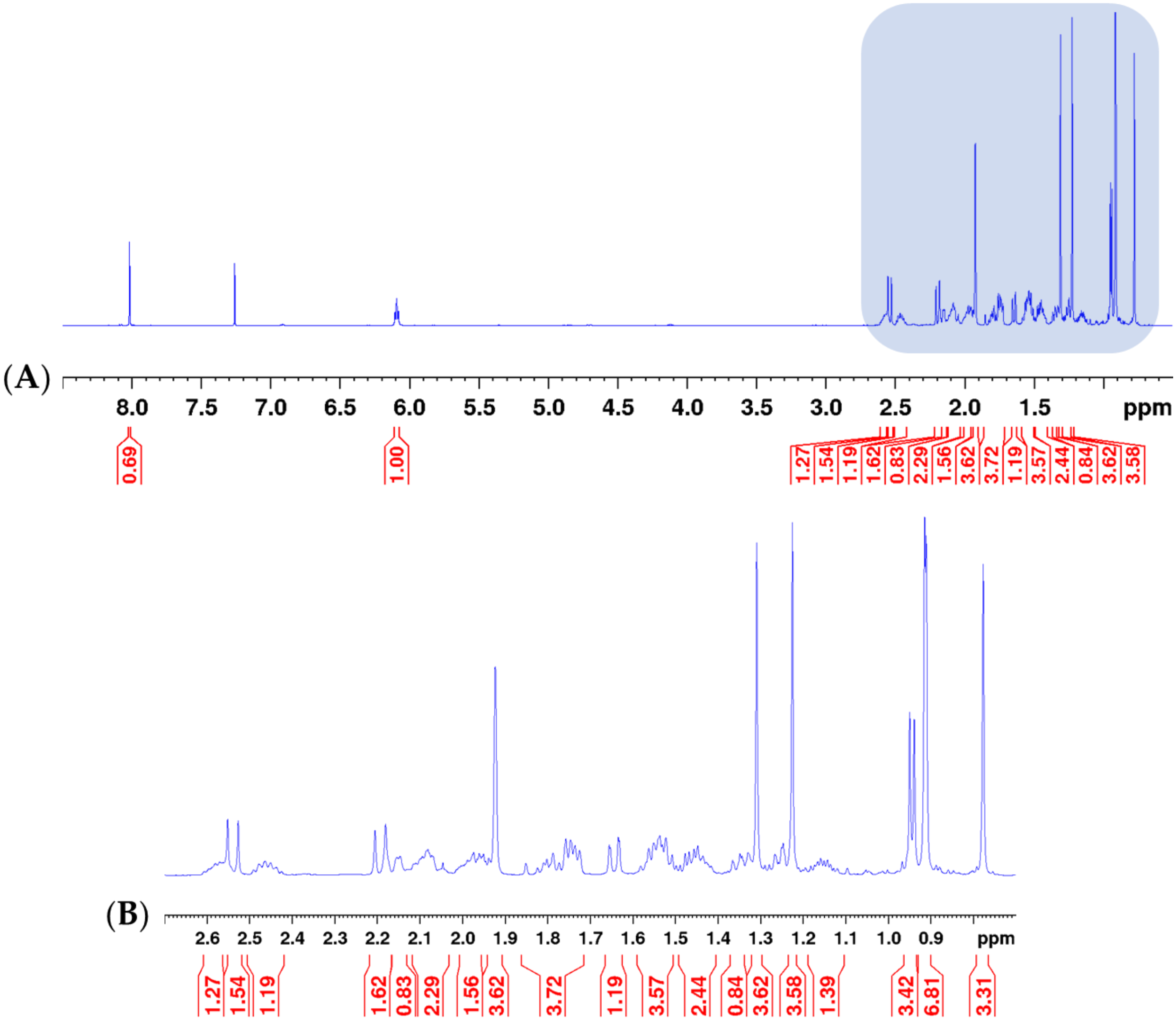

Figure S8: (A) ^{1}H (600 MHz, $CDCl_3$) spectrum of **3**. (B) Zoom region from 0.70 to 2.68 ppm.

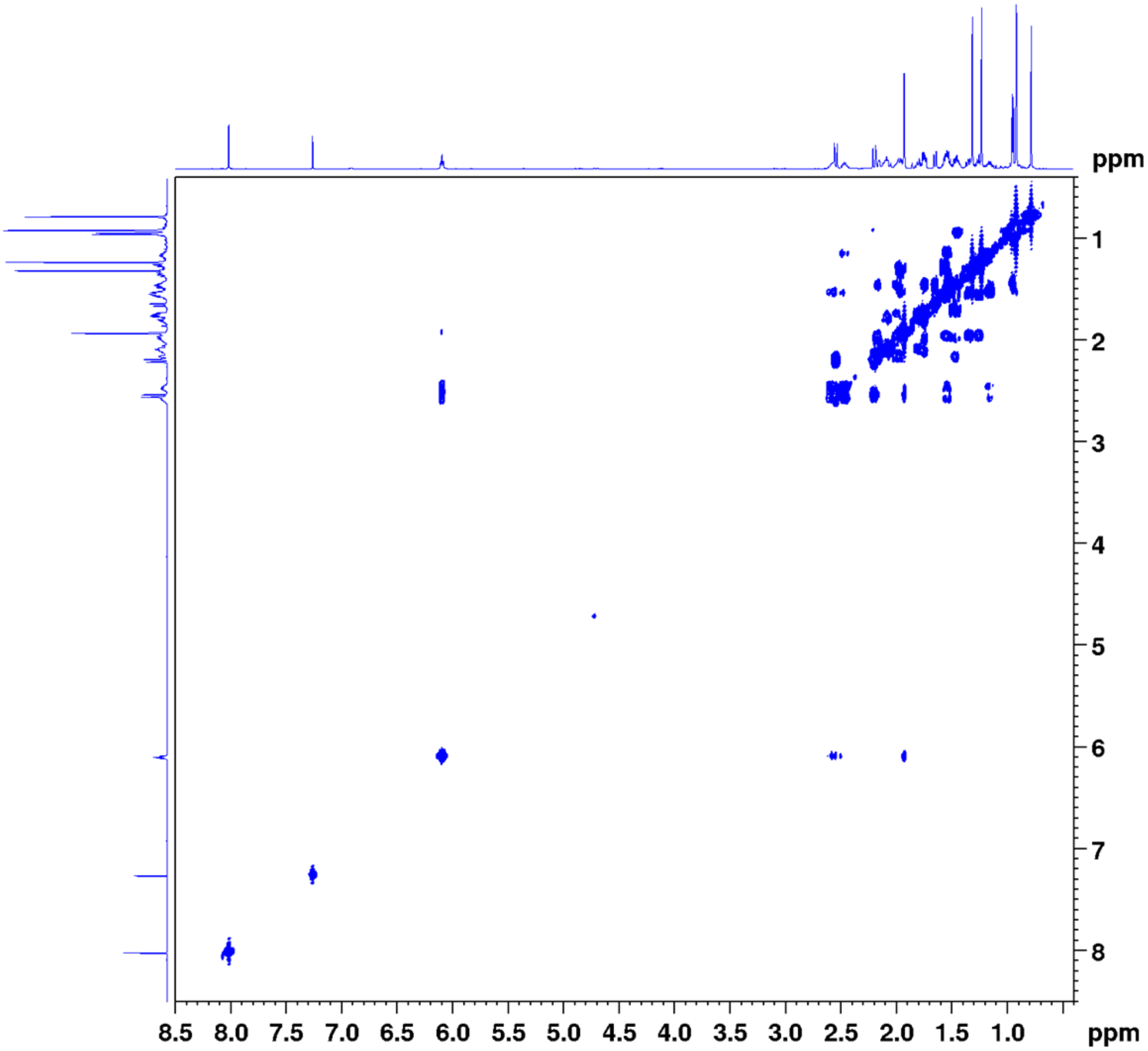

Figure S9: COSY (600 MHz, $CDCl_3$) spectrum of **3**.

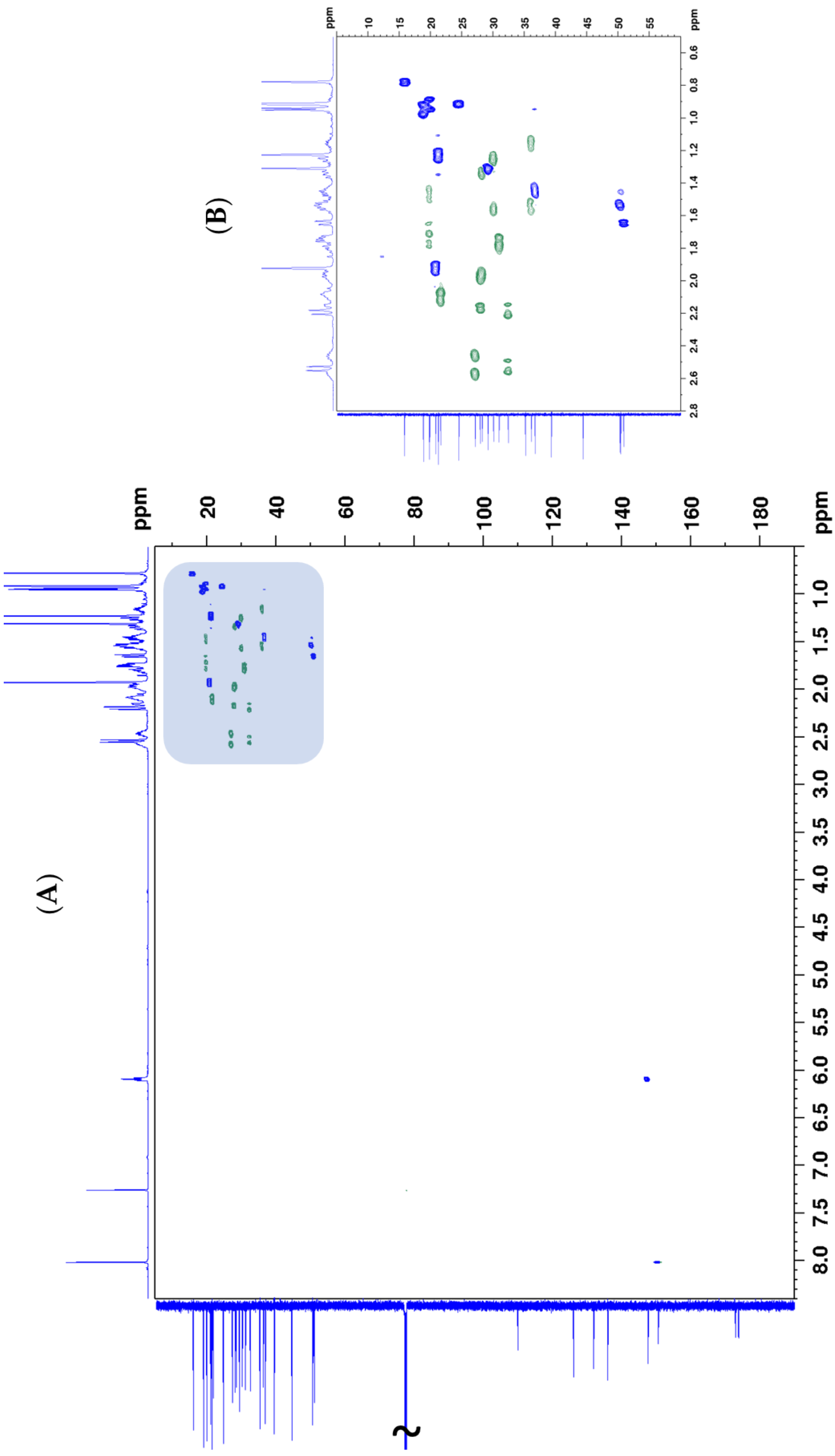

Figure S10: (A) HSQC (600 MHz, $CDCl_3$) spectrum of **3**. (B) Zoom region from 0.70 to 2.68 ppm.

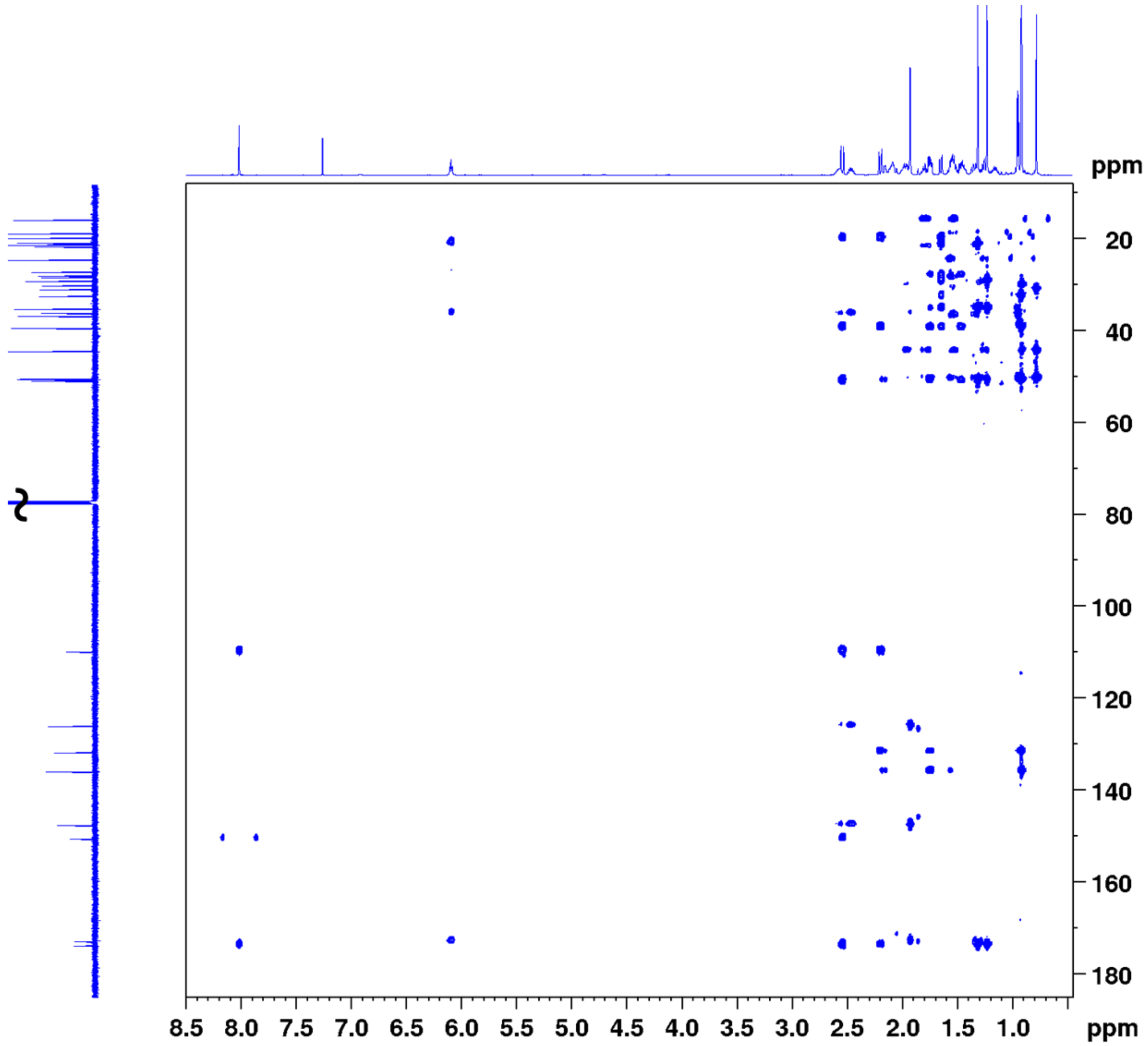

Figure S11: HMBC (600 MHz, $CDCl_3$) spectrum of **3**.

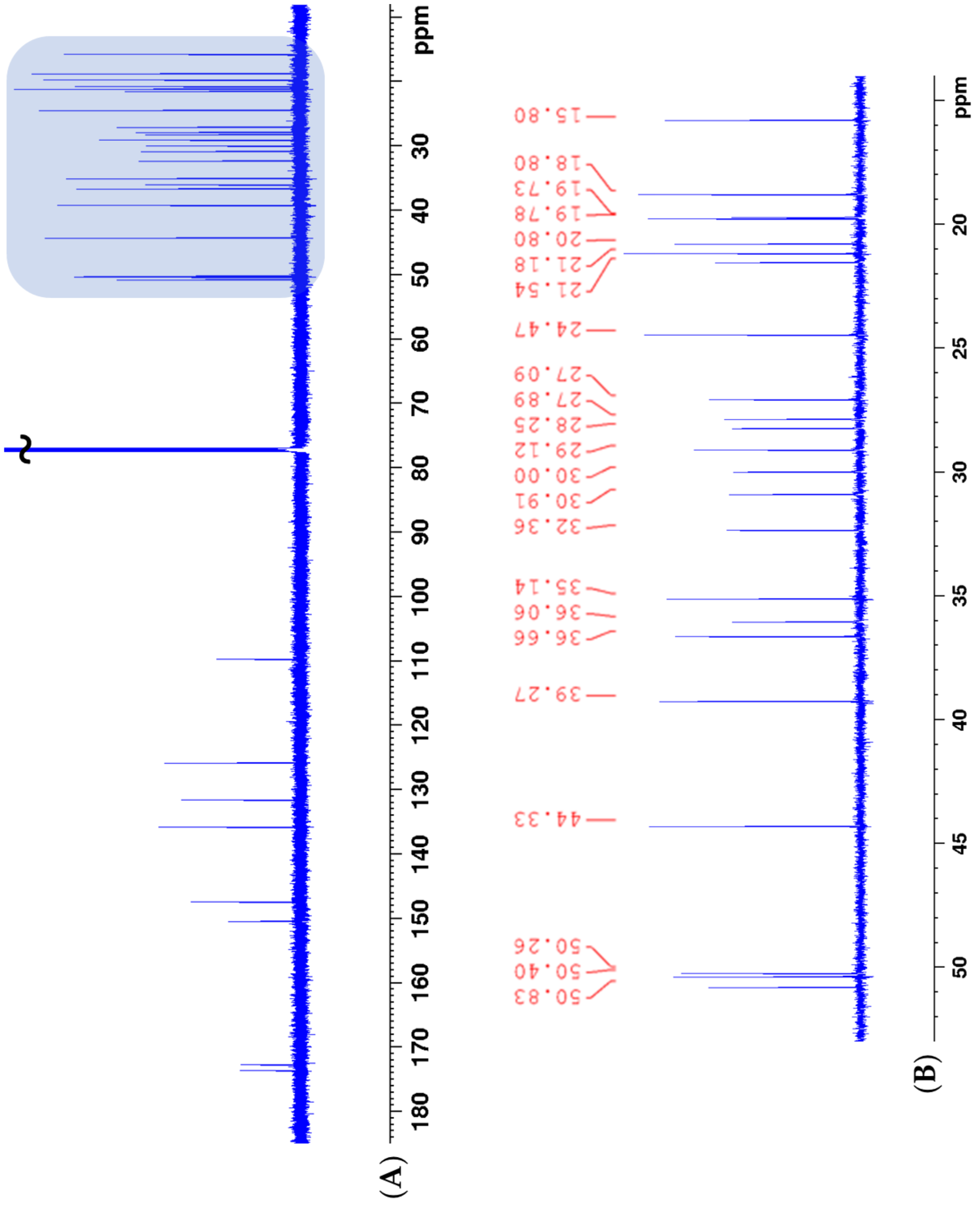

Figure S12: (A) ^{13}C (150 MHz, $CDCl_3$) spectrum of **3**. (B) Zoom region from 10.00 to 52.00 ppm.

2-bromo-3-oxotirucalla-8,24*Z*-dien-26-oic acid (4)

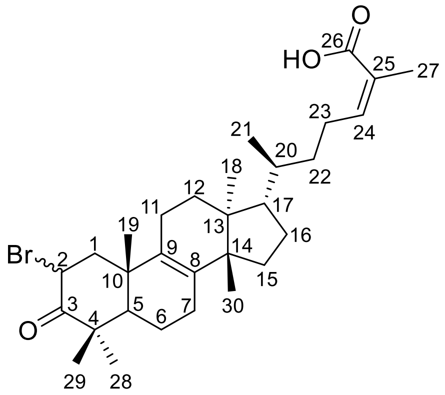

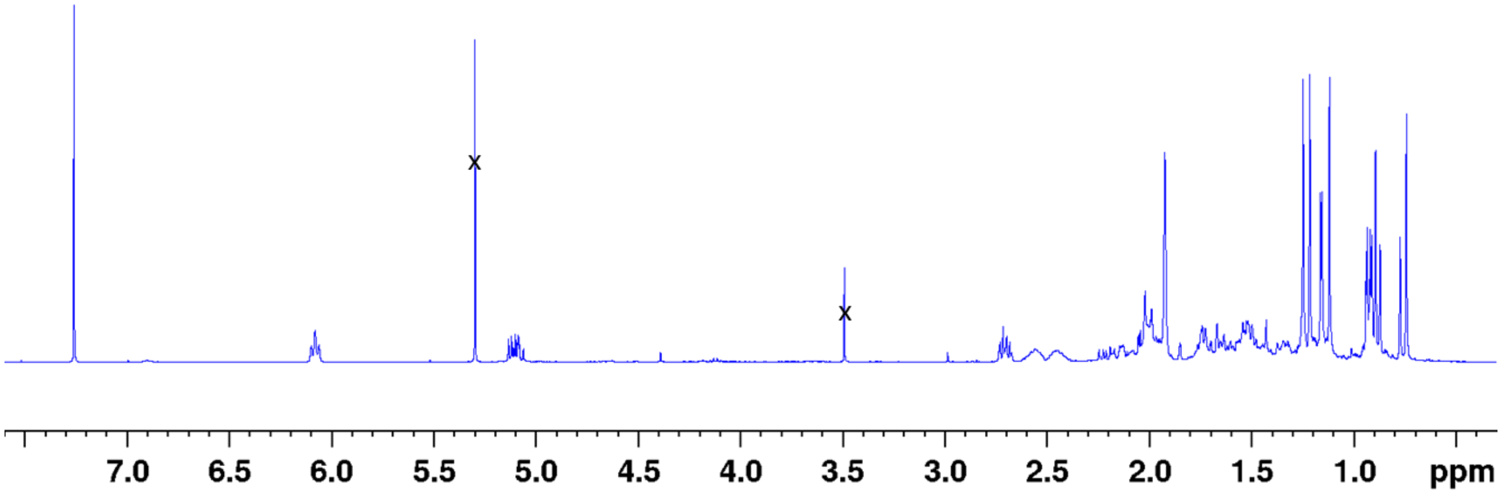

Figure S13: ^{1}H (400 MHz, $CDCl_3$) spectrum of **4**.

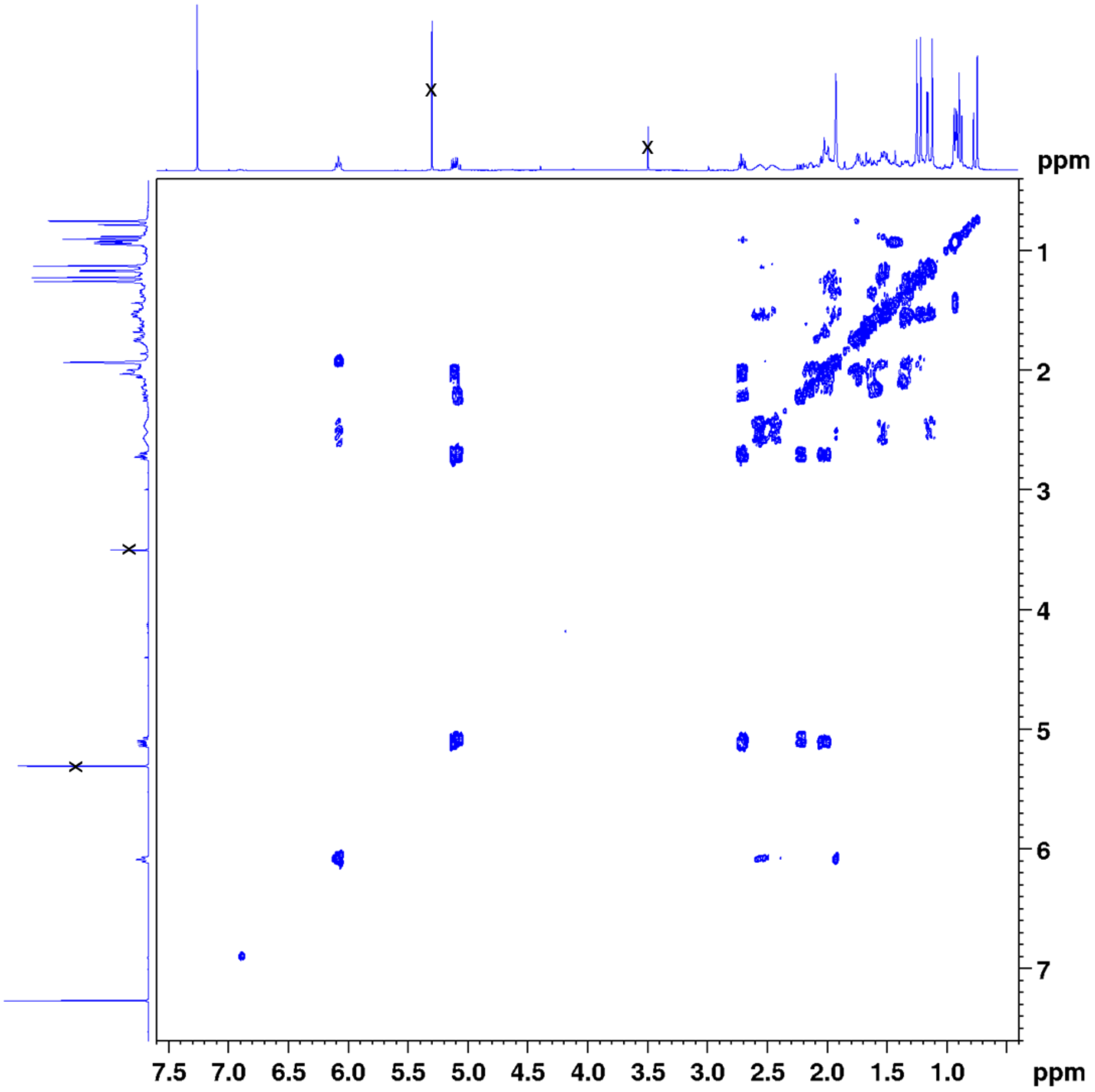

Figure S14: COSY (400 MHz, $CDCl_3$) spectrum of **4**.

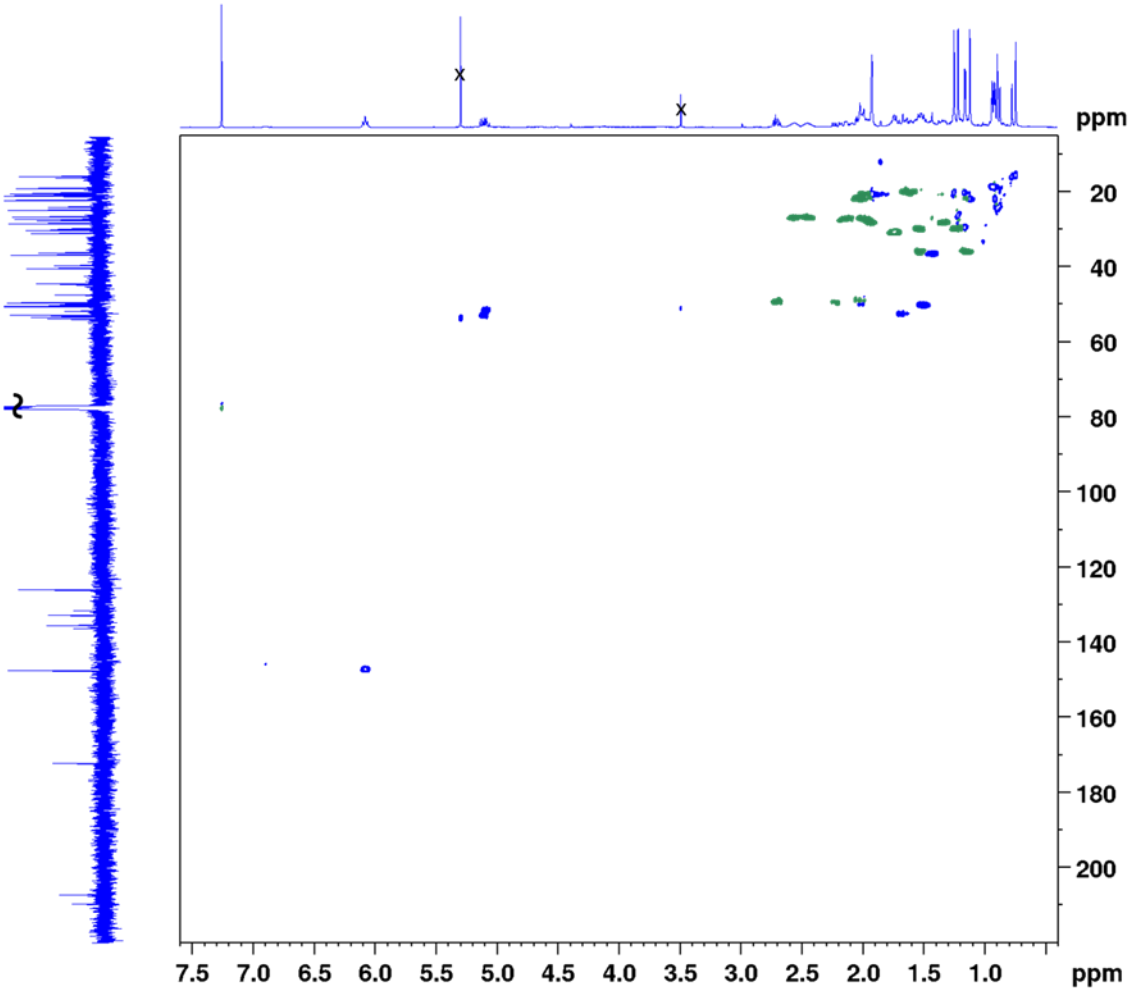

Figure S15: HSQC (400 MHz, $CDCl_3$) spectrum of **4**.

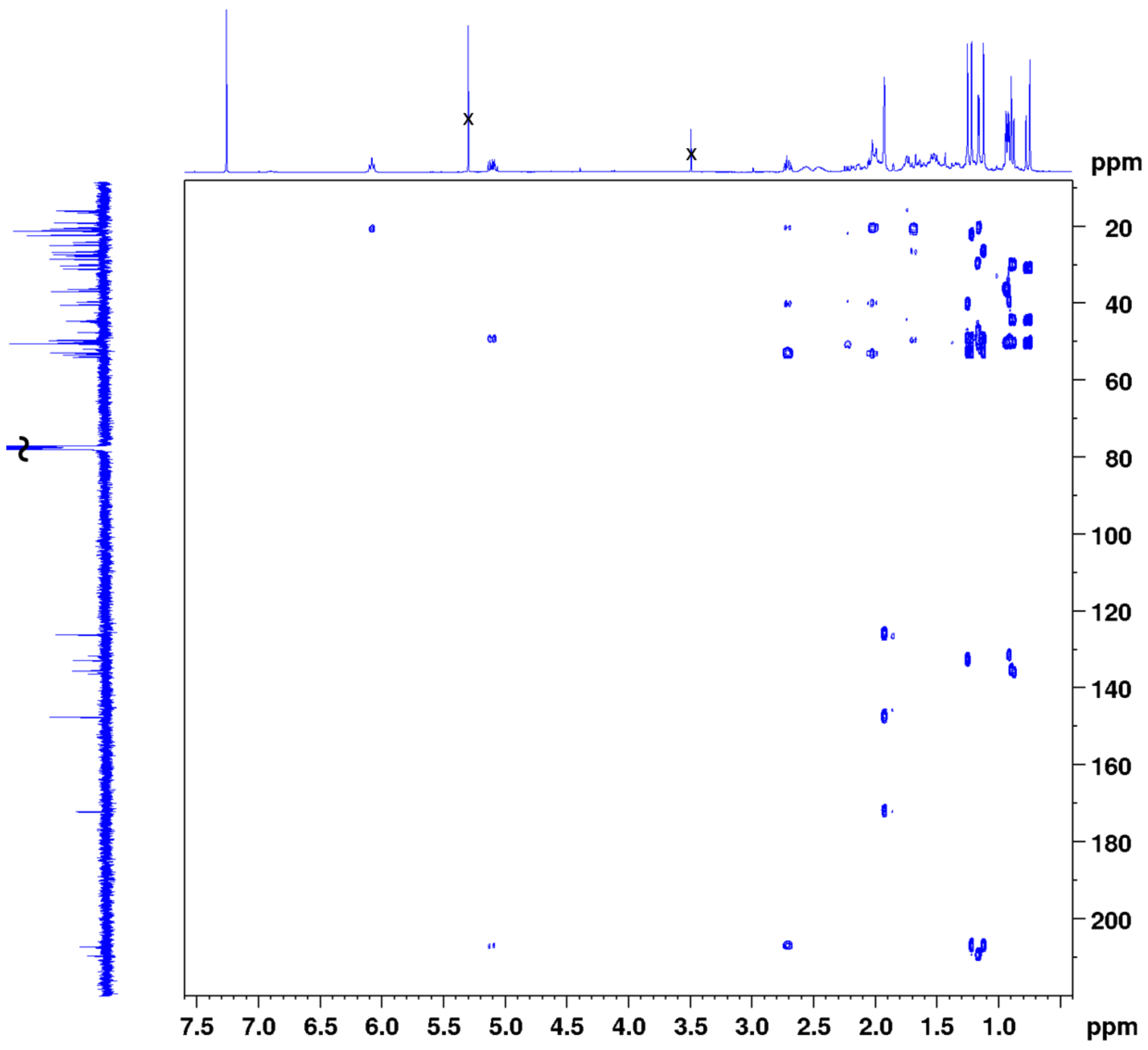

Figure S16: HMBC (400 MHz, $CDCl_3$) spectrum of **4**.

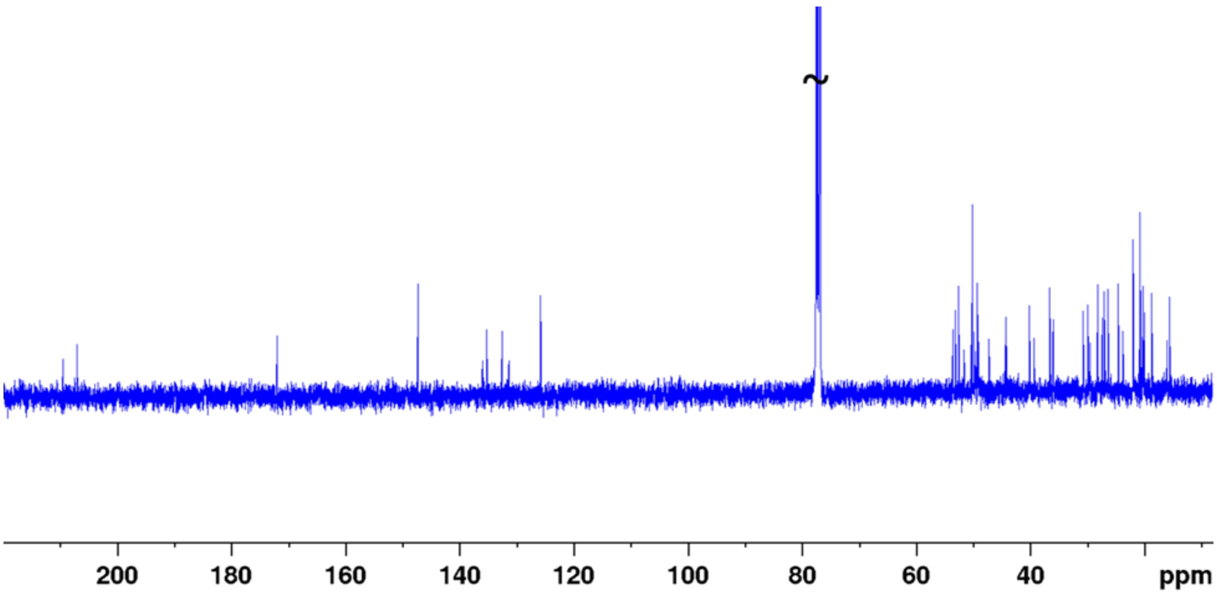

Figure S17: ^{13}C (100 MHz, $CDCl_3$) spectrum of **4**.

(S,Z)-6-((1S,3aS,5aR,10aS,12aS)-8-amino-3a,6,6,10a,12a-pentamethyl-2,3,3a,4,5,5a,6,10,10a,11,12,12a-dodecahydro-1H-cyclopenta[7,8]phenanthro[2,3-d]thiazol-1-yl)-2-methylhept-2-enoic acid (5)

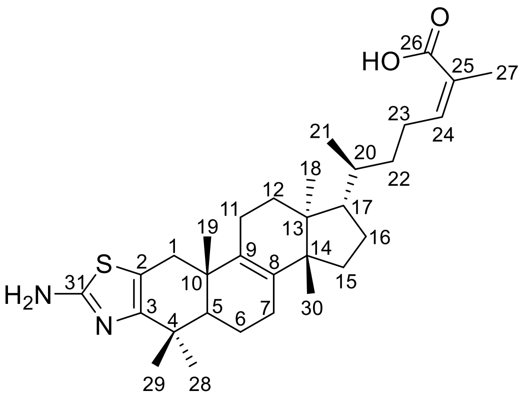

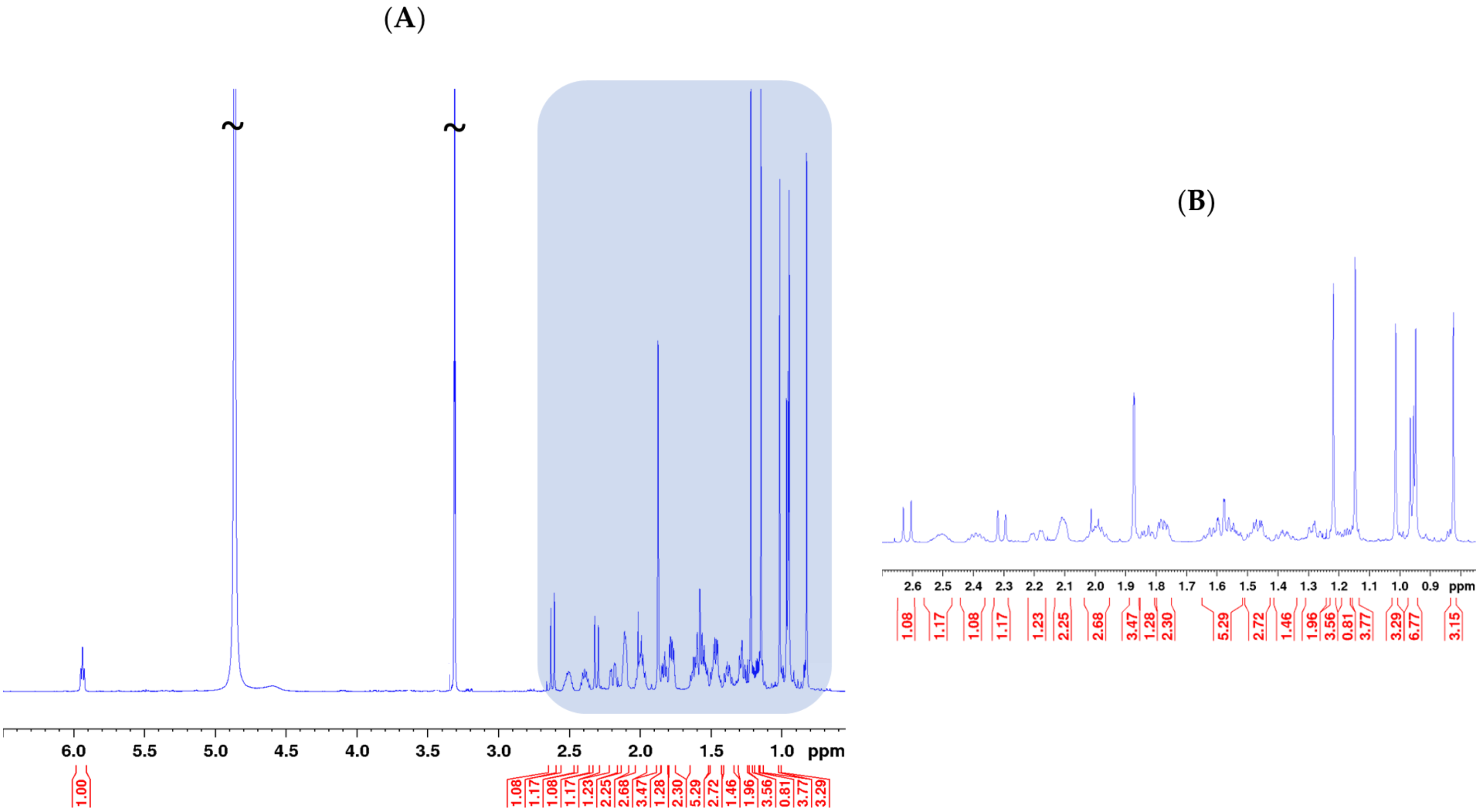

Figure S18: (A) ^{1}H (600 MHz, MeOD) spectrum of **5**. (B) Zoom region from 0.70 to 2.65 ppm.

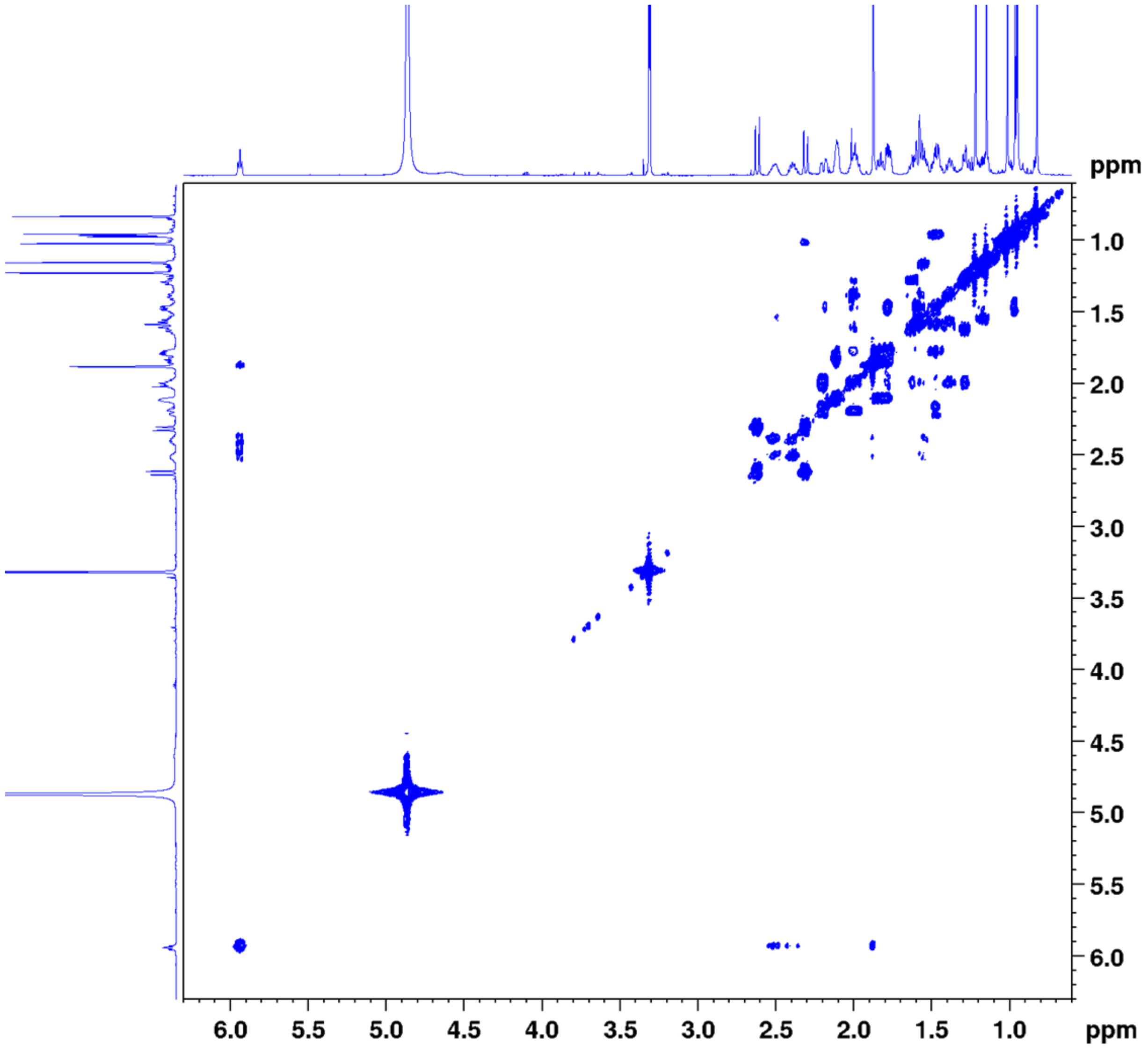

Figure S19: COSY (600 MHz, MeOD) spectrum of **5**.

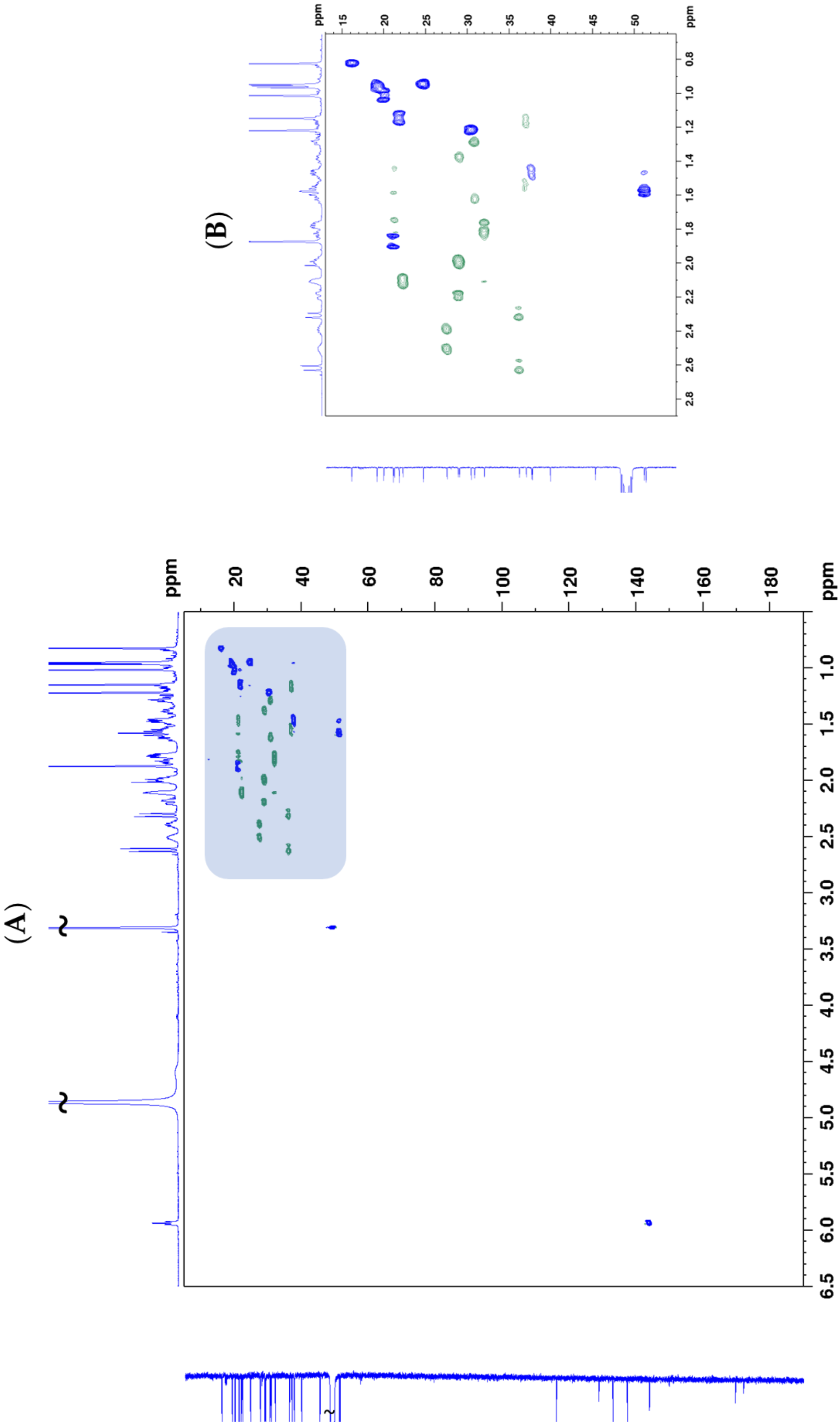

Figure S20: (A) HSQC (600 MHz, MeOD) spectrum of **5**. (B) Zoom region from 0.70 to 2.80 ppm.

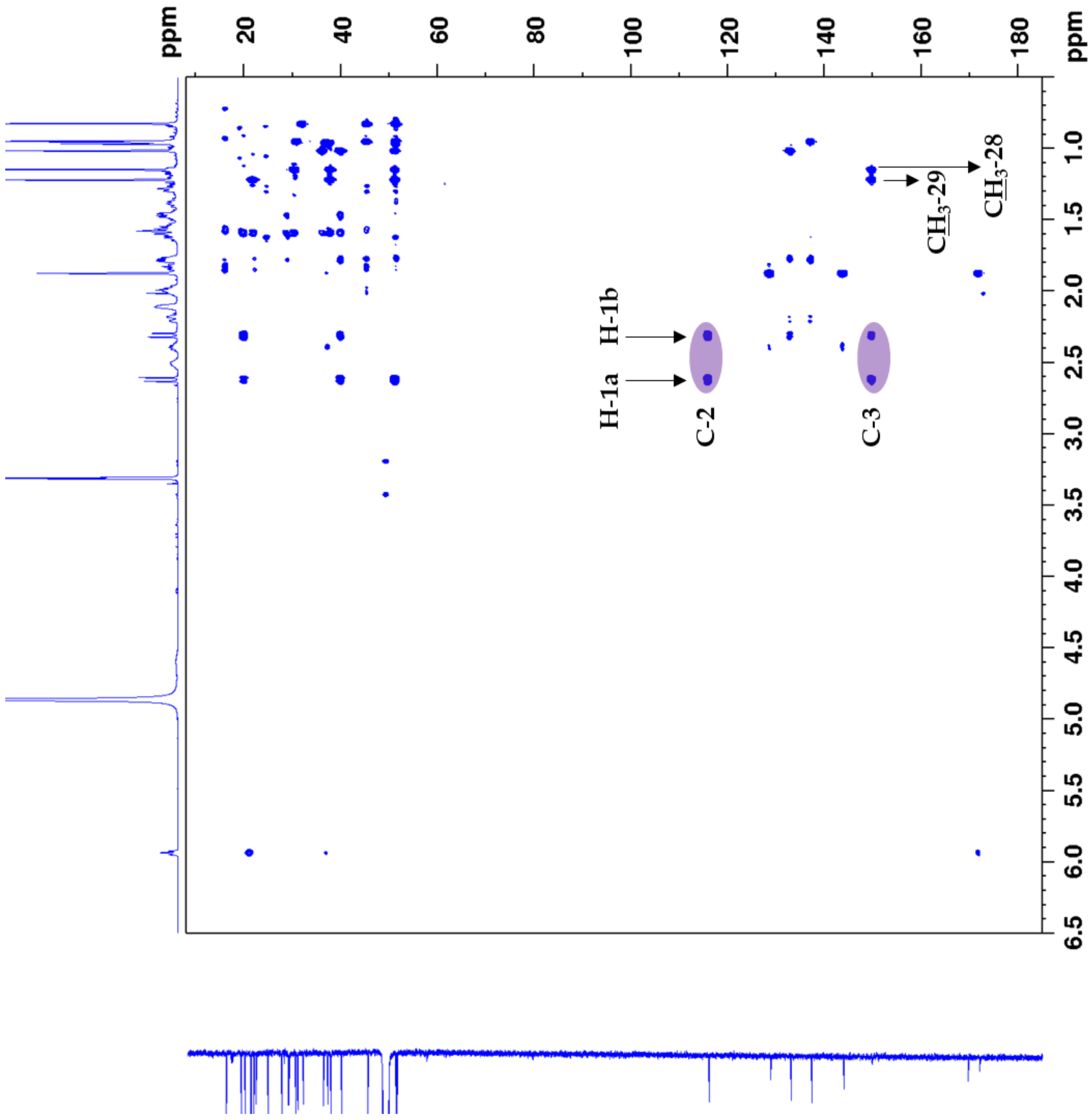

Figure S21: HMBC (600 MHz, MeOD) spectrum of **5**.

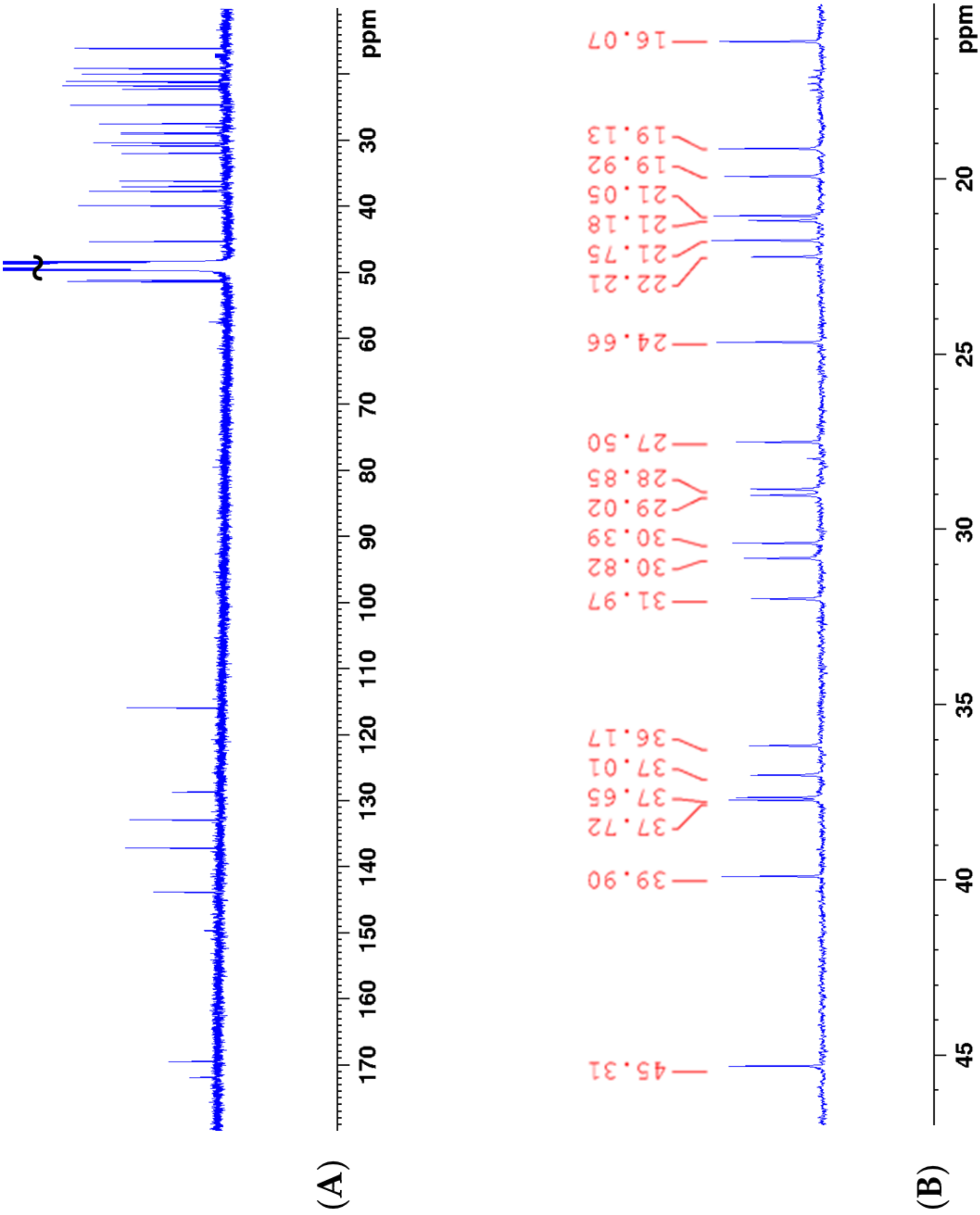

Figure S22: (A) ^{13}C (100 MHz, MeOD) spectrum of **5**. (B) Zoom region from 16.00 to 46.00 ppm.

2-thiocyanate-3-oxotirucalla-8,24*Z*-dien-26-oic acid (6)

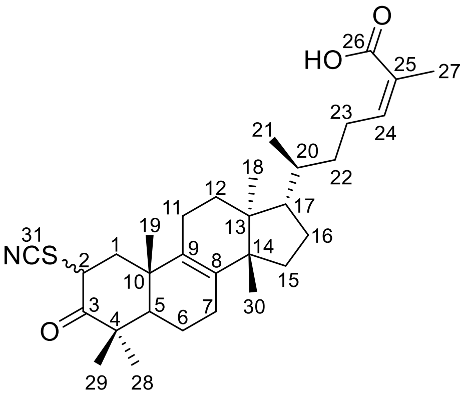

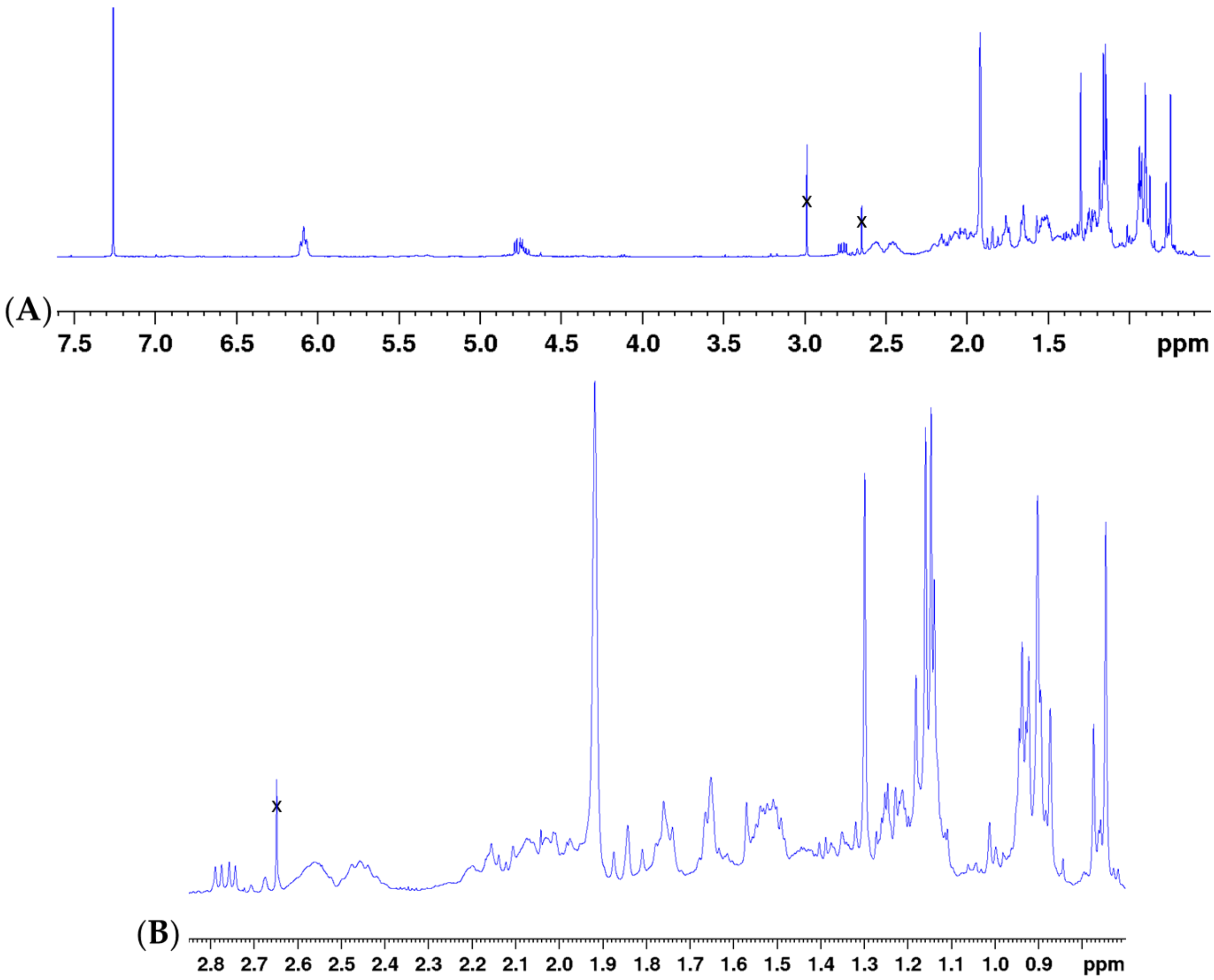

Figure S23: (A) ^{1}H (400 MHz, $CDCl_3$) spectrum of **6**. (B) Zoom region from 0.70 to 2.88 ppm.

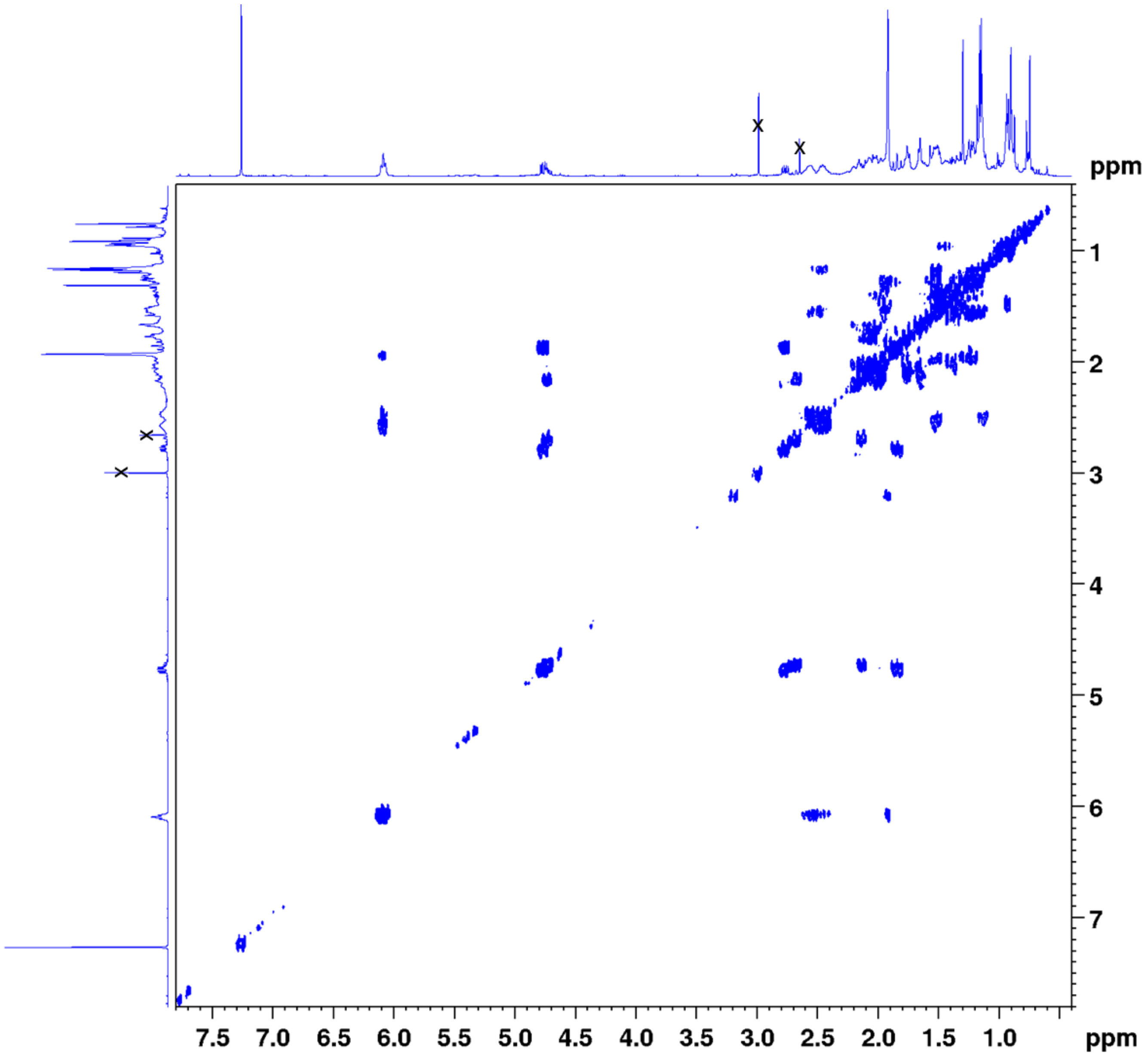

Figure S24: COSY (400 MHz, $CDCl_3$) spectrum of **6**.

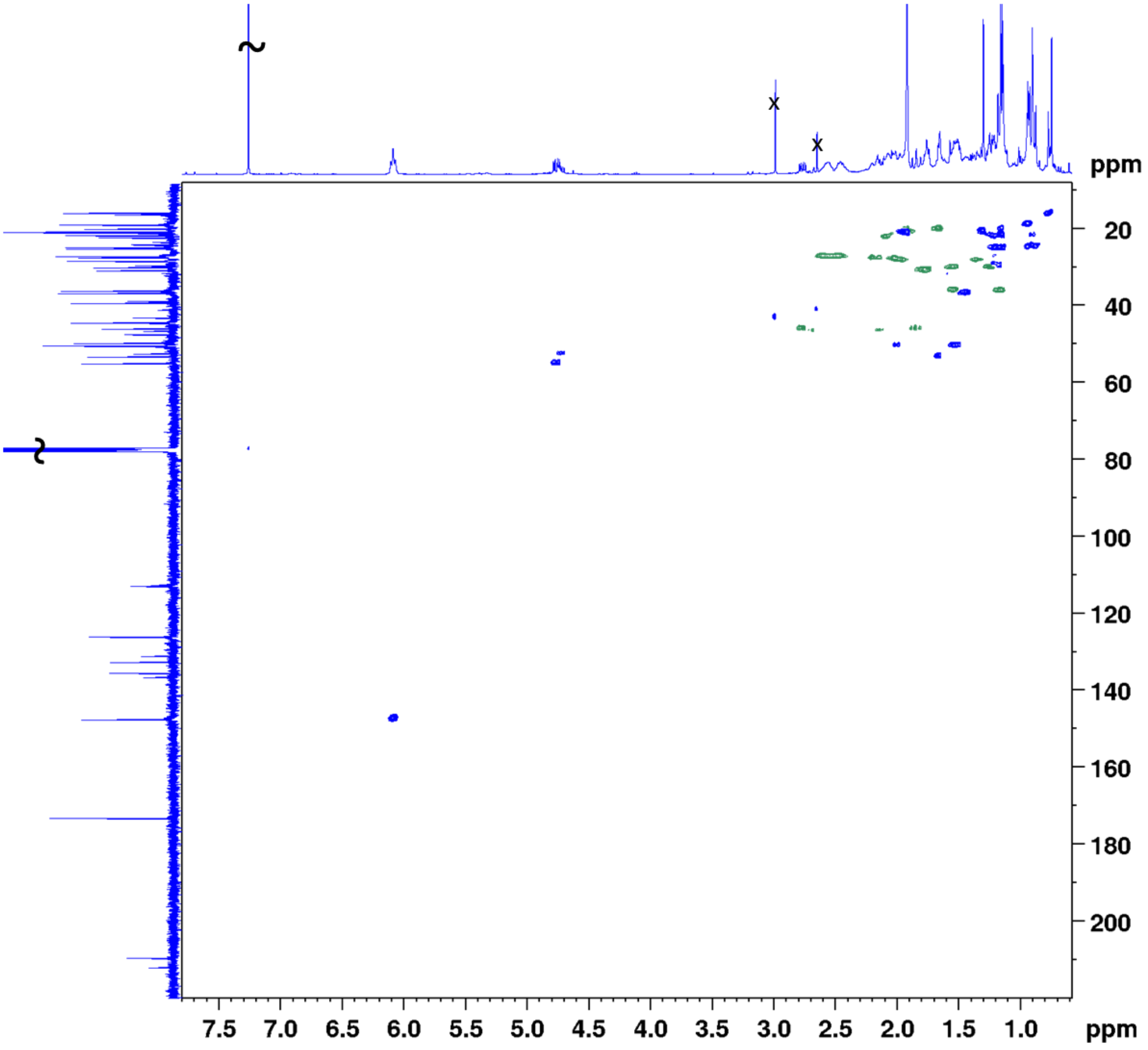

Figure S25: HSQC (400 MHz, $CDCl_3$) spectrum of **6**.

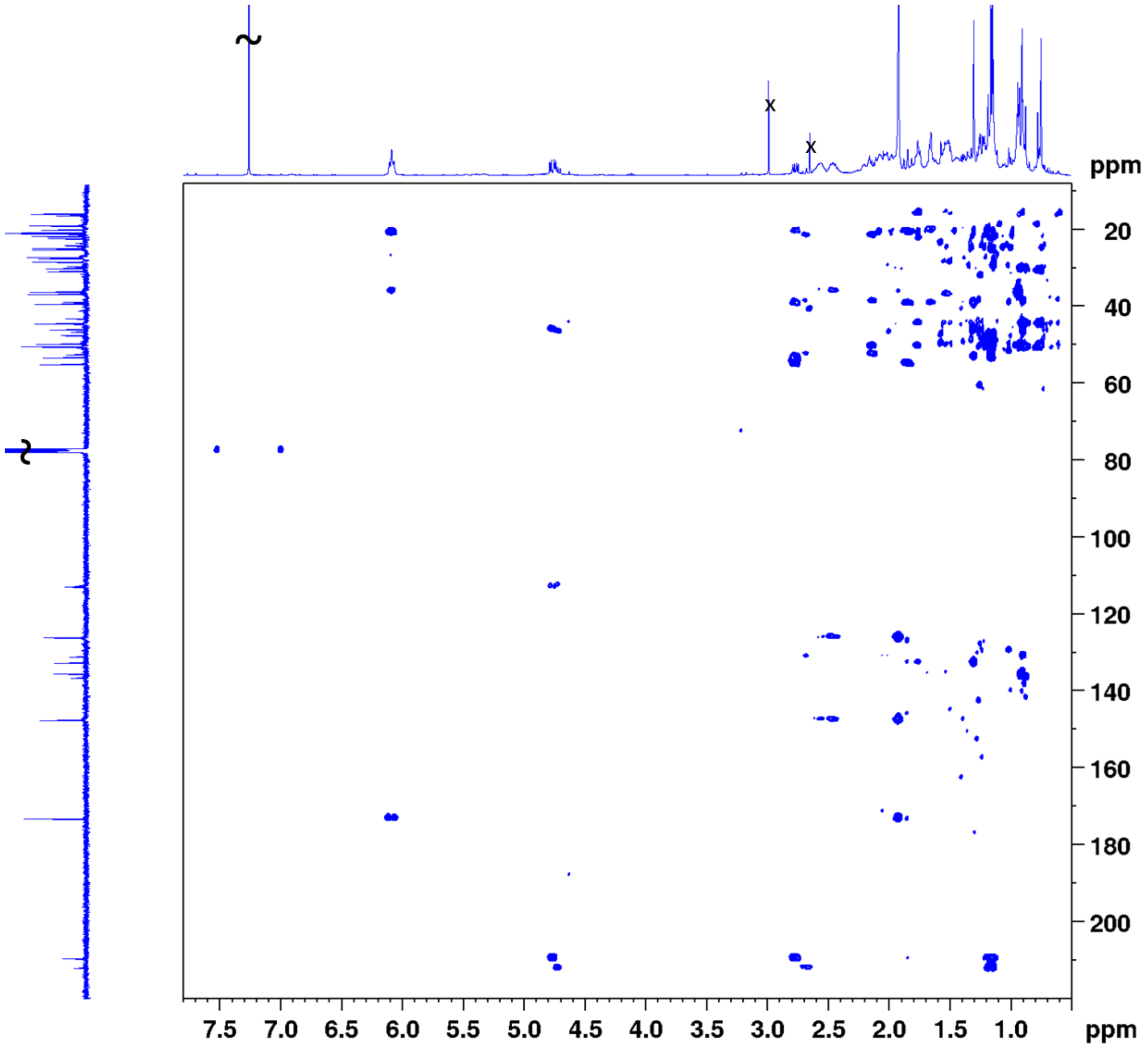

Figure S26: HMBC (400 MHz, $CDCl_3$) spectrum of **6**.

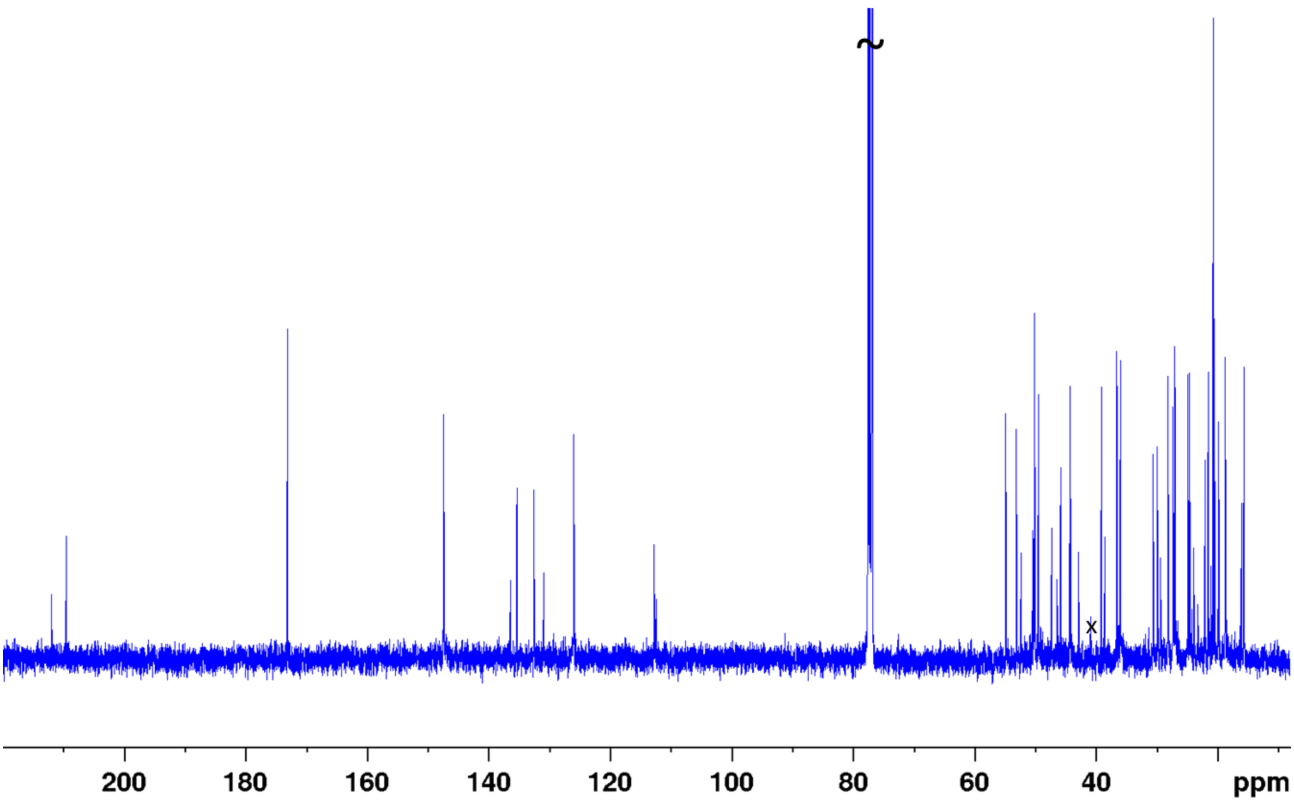

Figure S27: ^{13}C (100 MHz, $CDCl_3$) spectrum of **6**.

(S,Z)-2-methyl-6-((1S,3aS,5aR,10aS,12aS)-3a,6,6,10a,12a-pentamethyl-8-morpholino-2,3,3a,4,5,5a,6,10,10a,11,12,12a-dodecahydro-1H-cyclopenta[7,8]phenanthro[2,3-d]thiazol-1-yl)hept-2-enoic acid (7)

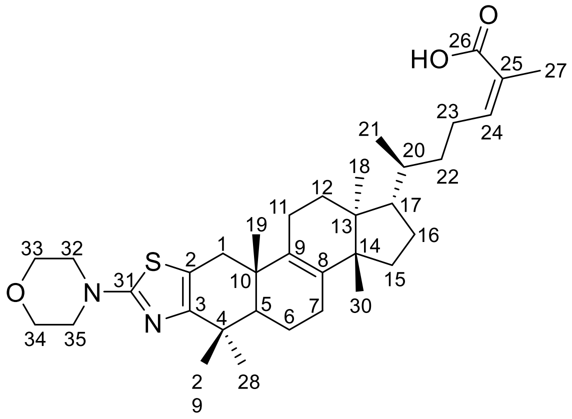

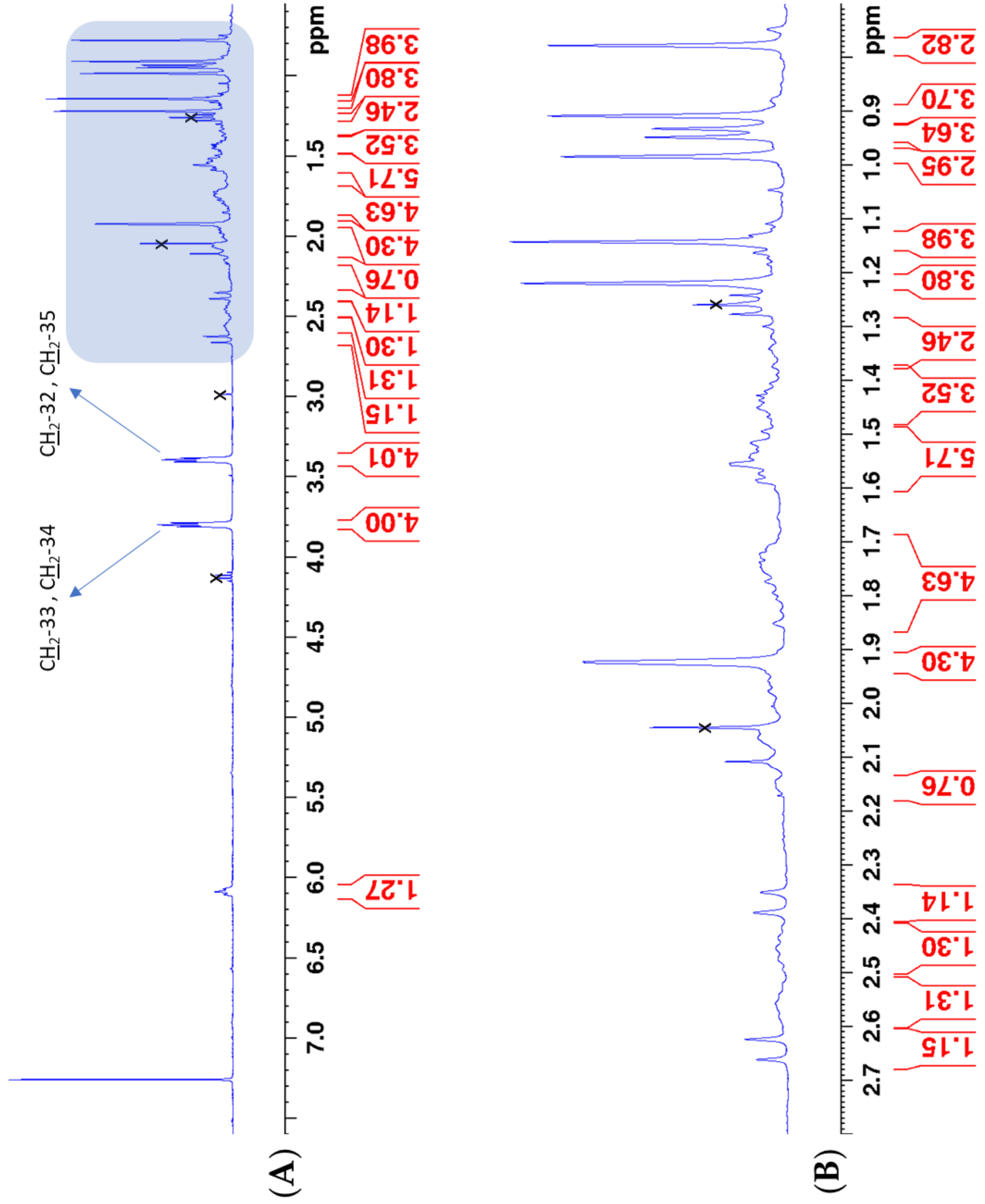

Figure S28: (A) ^{1}H (400 MHz, $CDCl_3$) spectrum of **7**. (B) Zoom region from 0.60 to 7.50 ppm.

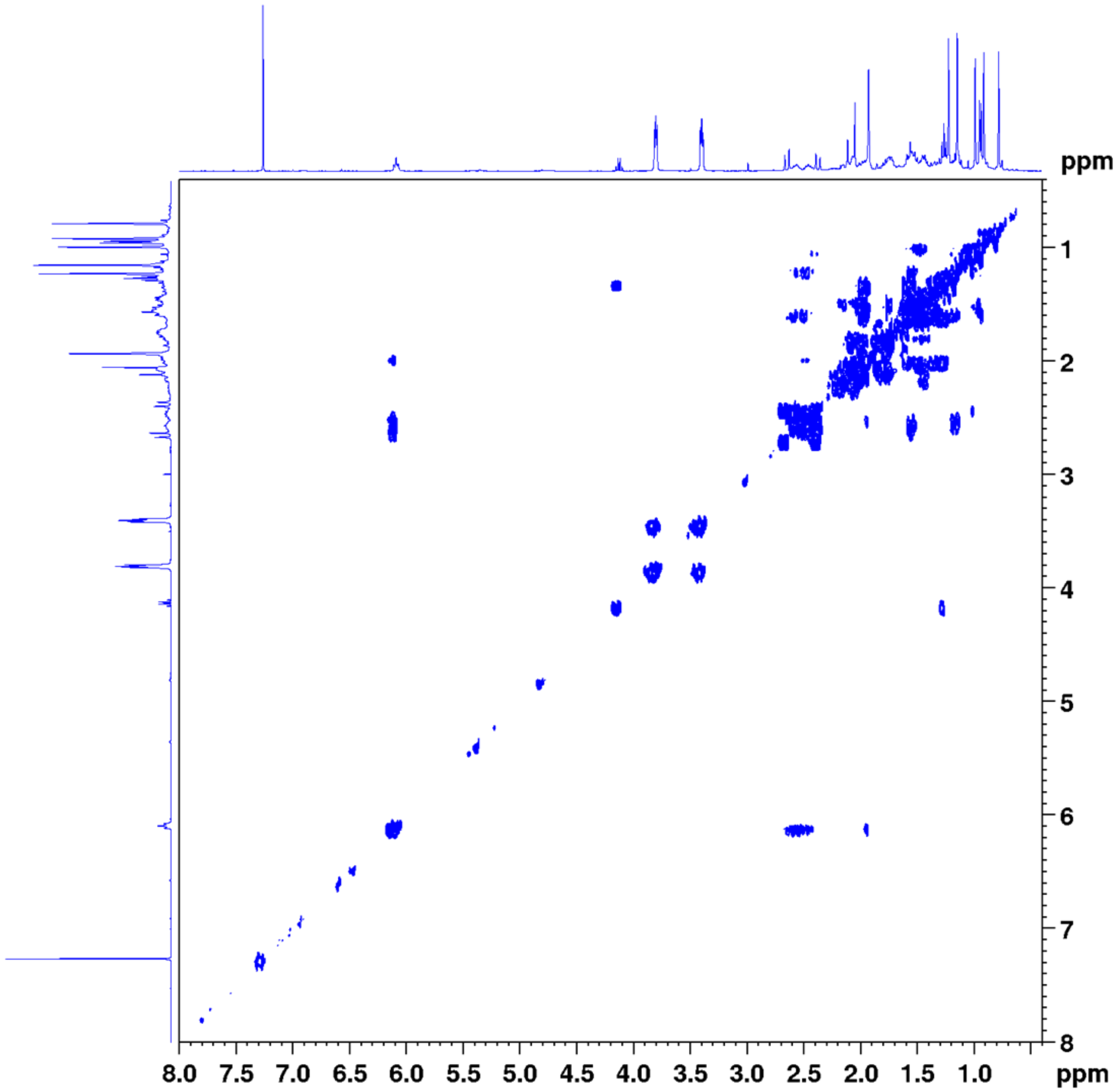

Figure S29: COSY (400 MHz, $CDCl_3$) spectrum of **7**.

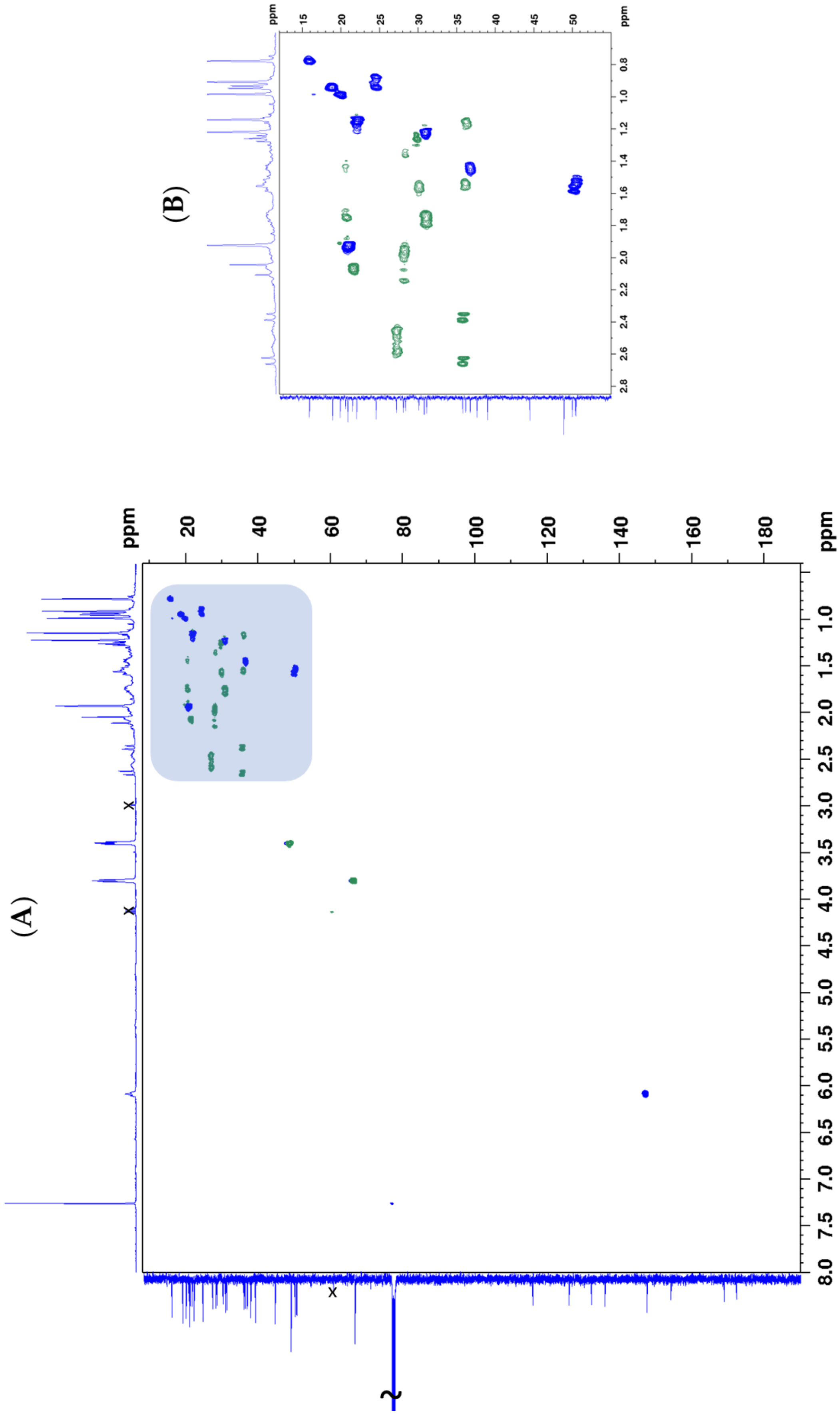

Figure S30: (A) HSQC (400 MHz, $CDCl_3$) spectrum of **7**. (B) Zoom region from 0.70 to 2.80 ppm.

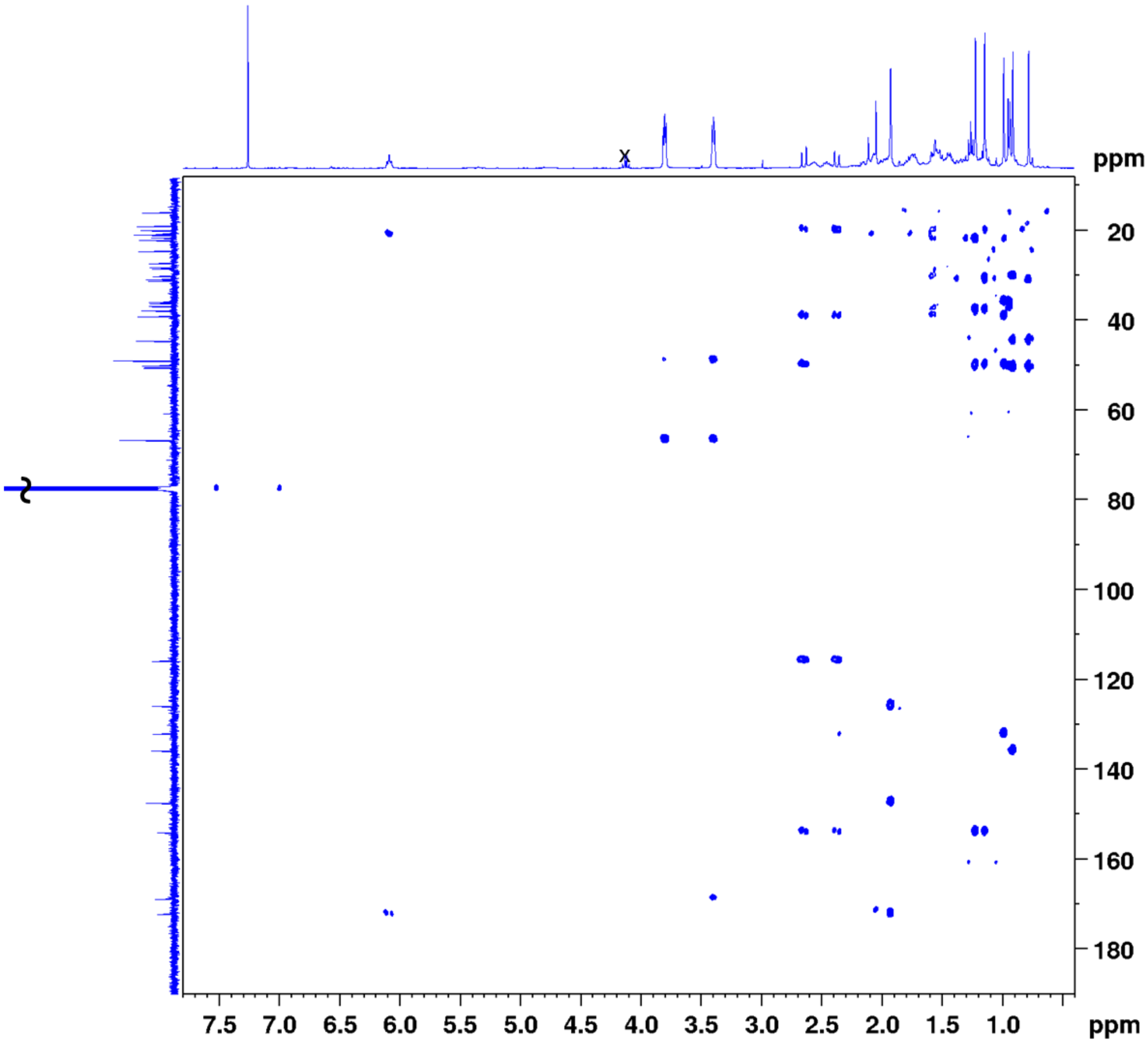

Figure S31: HMBC (400 MHz, $CDCl_3$) spectrum of **7**.

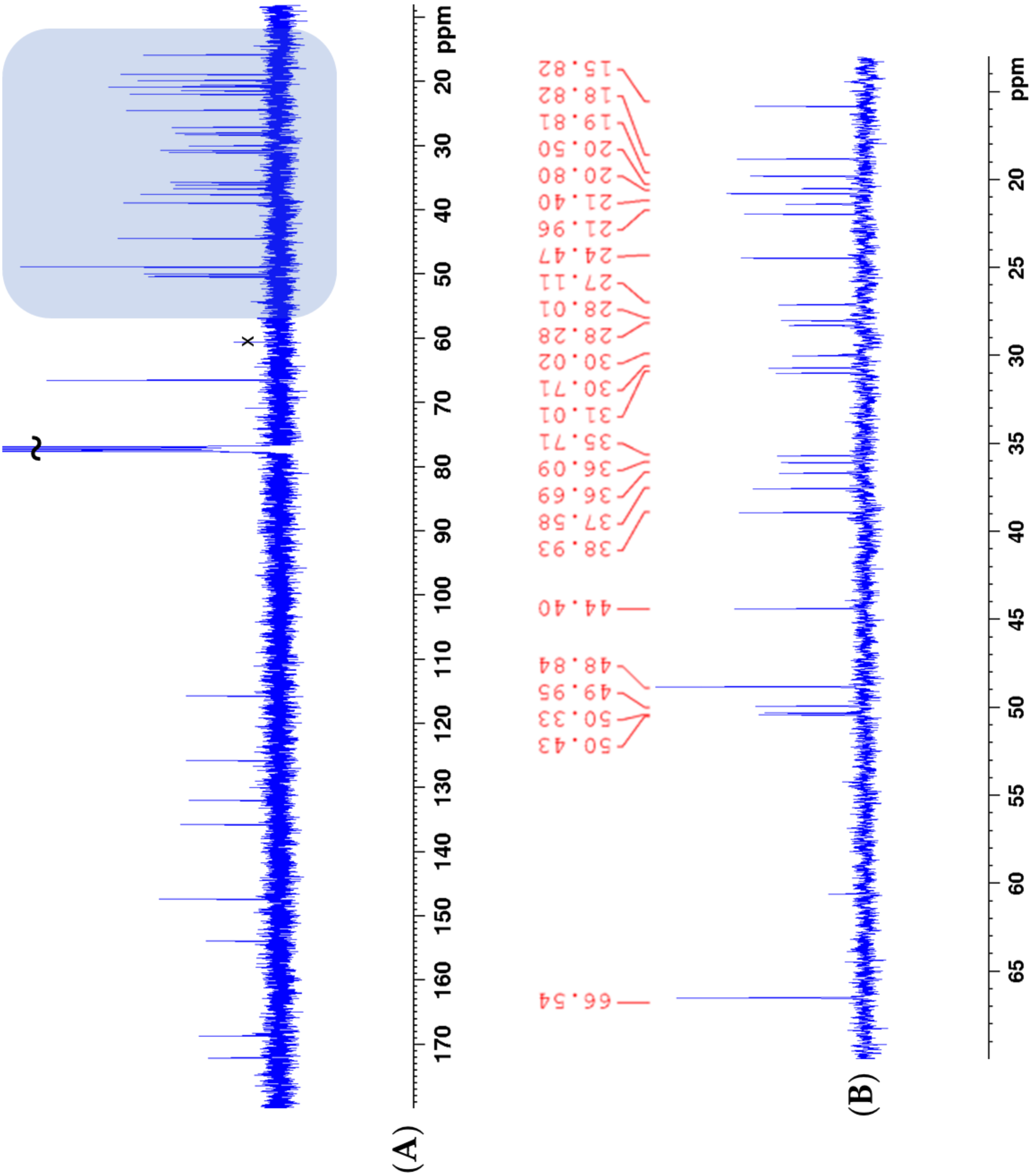

Figure S32: (A) ^{13}C (100 MHz, $CDCl_3$) spectrum of **7**. (B) Zoom region from 15.00 to 69.00 ppm.

2-hydroxy-3-oxotirucalla-8,24*Z*-dien-26-oic acid (8)

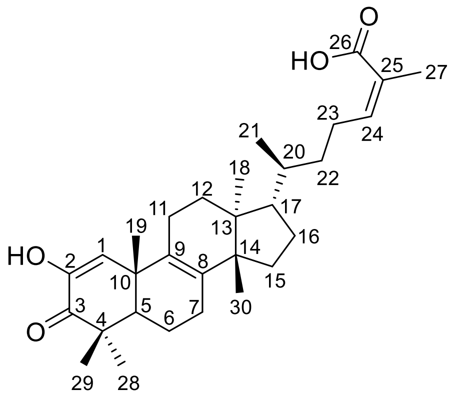

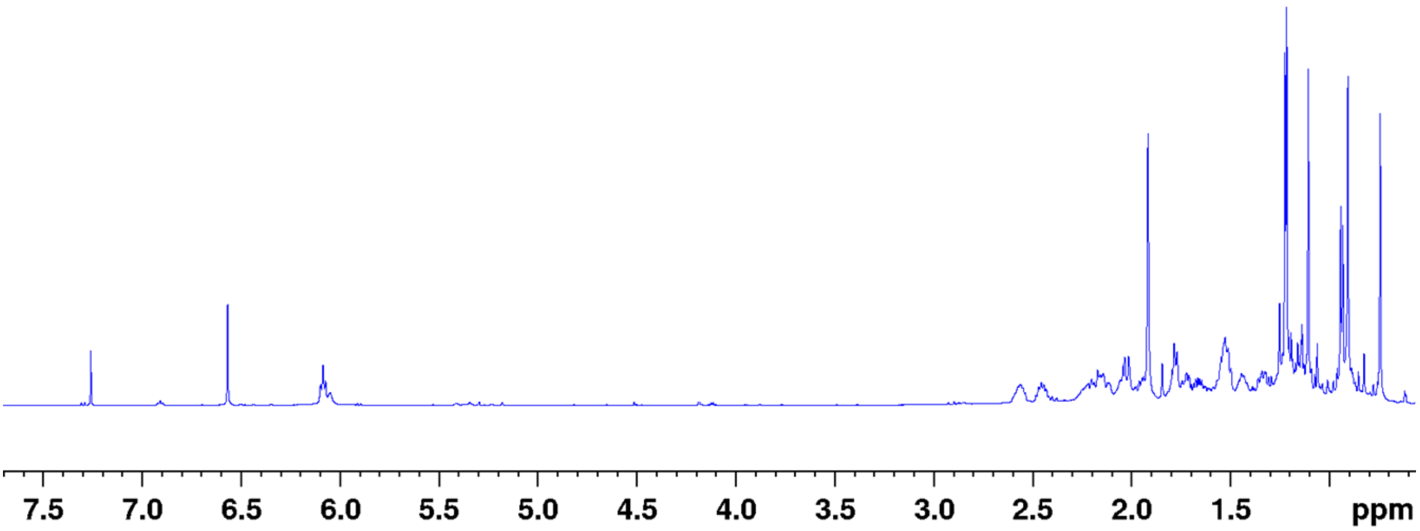

Figure S33: ^{1}H (600 MHz, $CDCl_3$) spectrum of **8**.

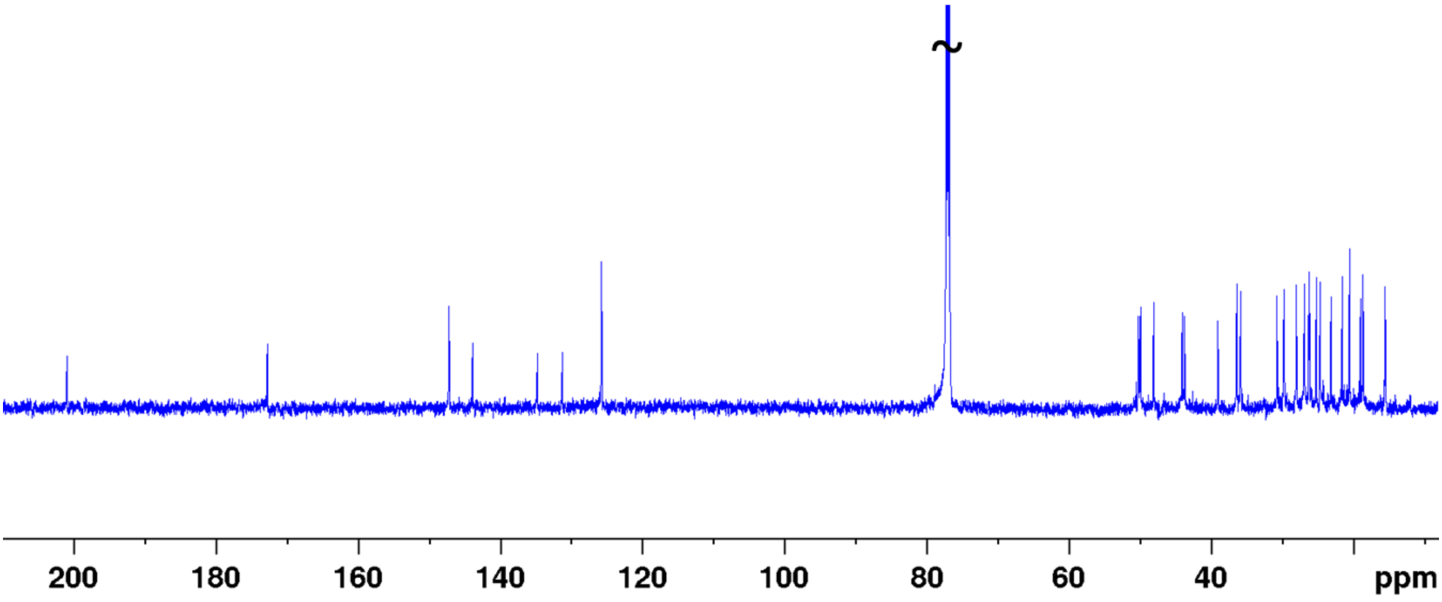

Figure S34: ^{13}C (150 MHz, $CDCl_3$) spectrum of **8**.

1α-hydroxy-3-oxa-nor- tirucalla-8,24Z-dien-27-oic acid (9)

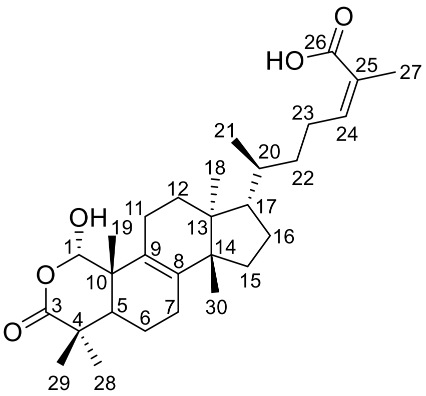

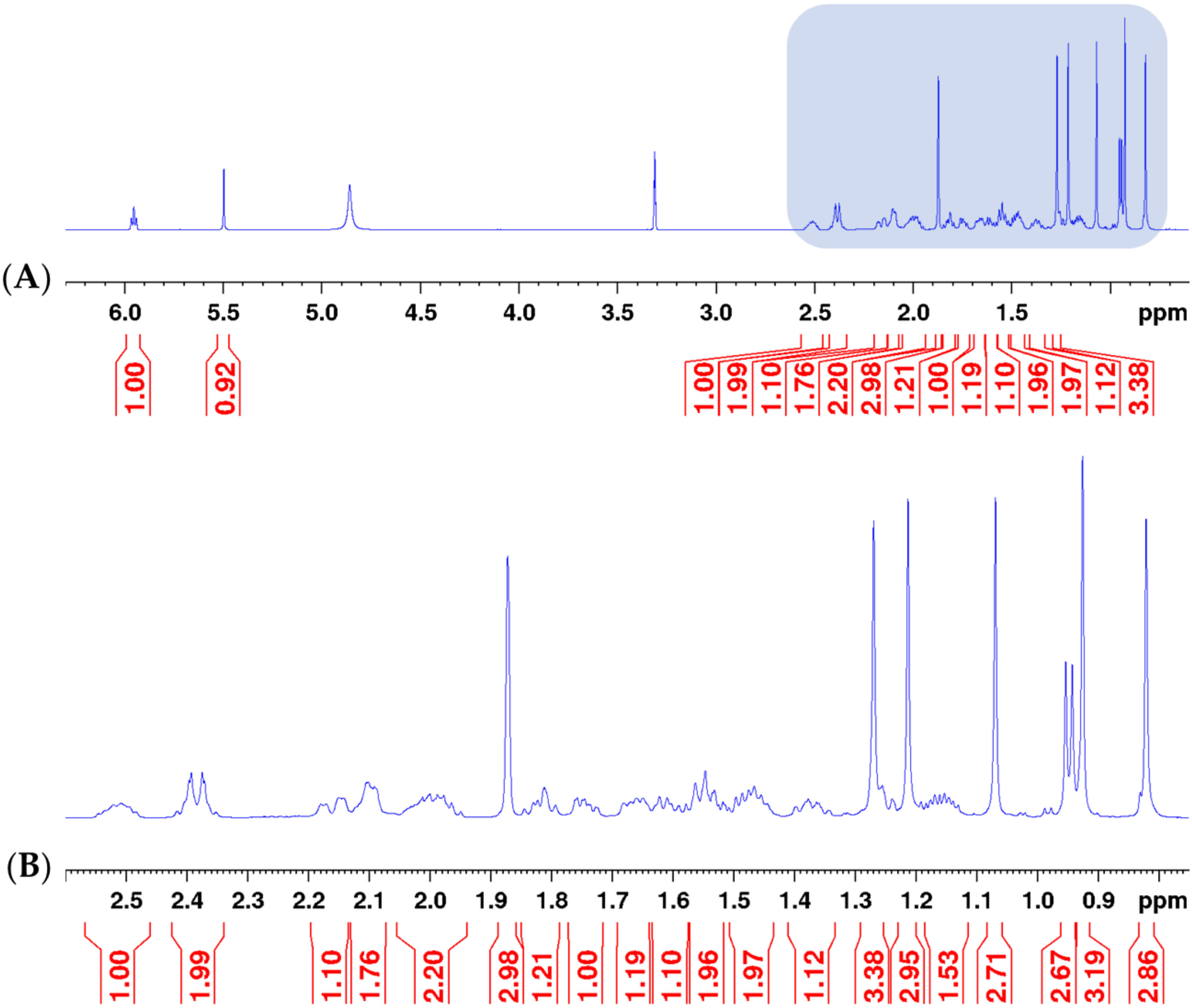

Figure S35: (A) ^{1}H (600 MHz, MeOD) spectrum of **9**. (B) Zoom region from 0.80 to 2.60 ppm.

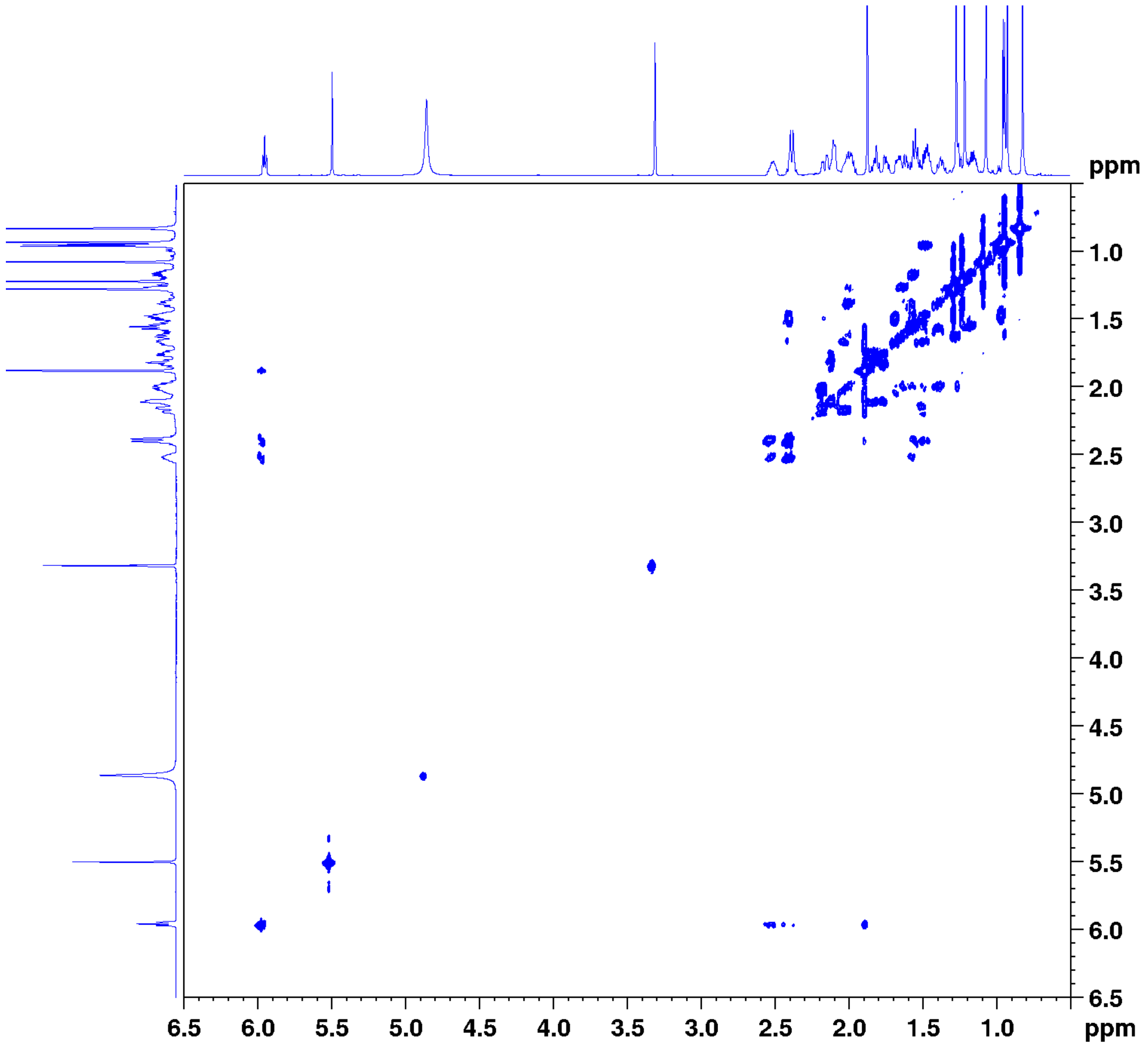

Figure S36: COSY (600 MHz, MeOD) spectrum of **9**.

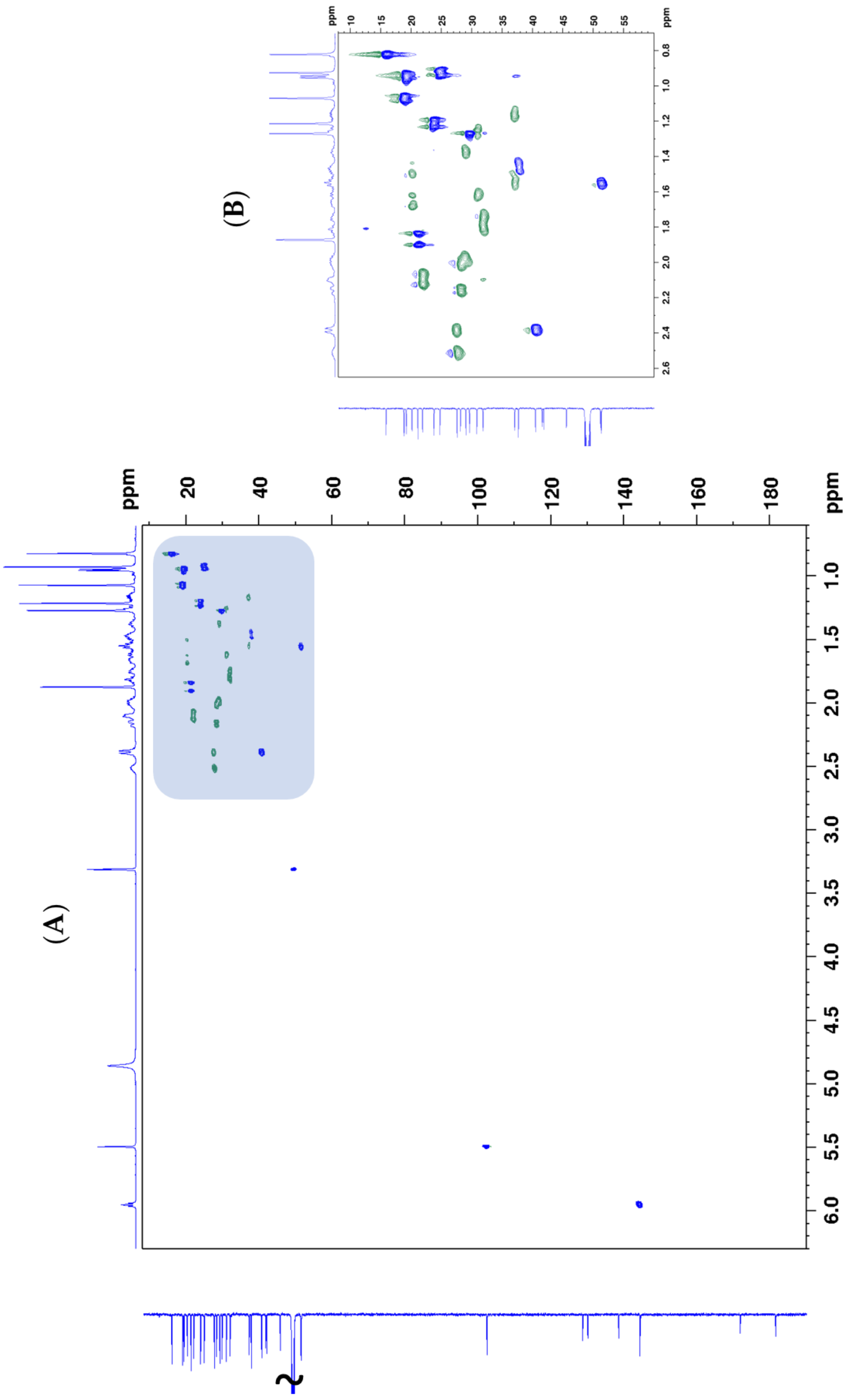

Figure S37: (A) HSQC (600 MHz, MeOD) spectrum of **9**. (B) Zoom region from 0.70 to 2.65 ppm.

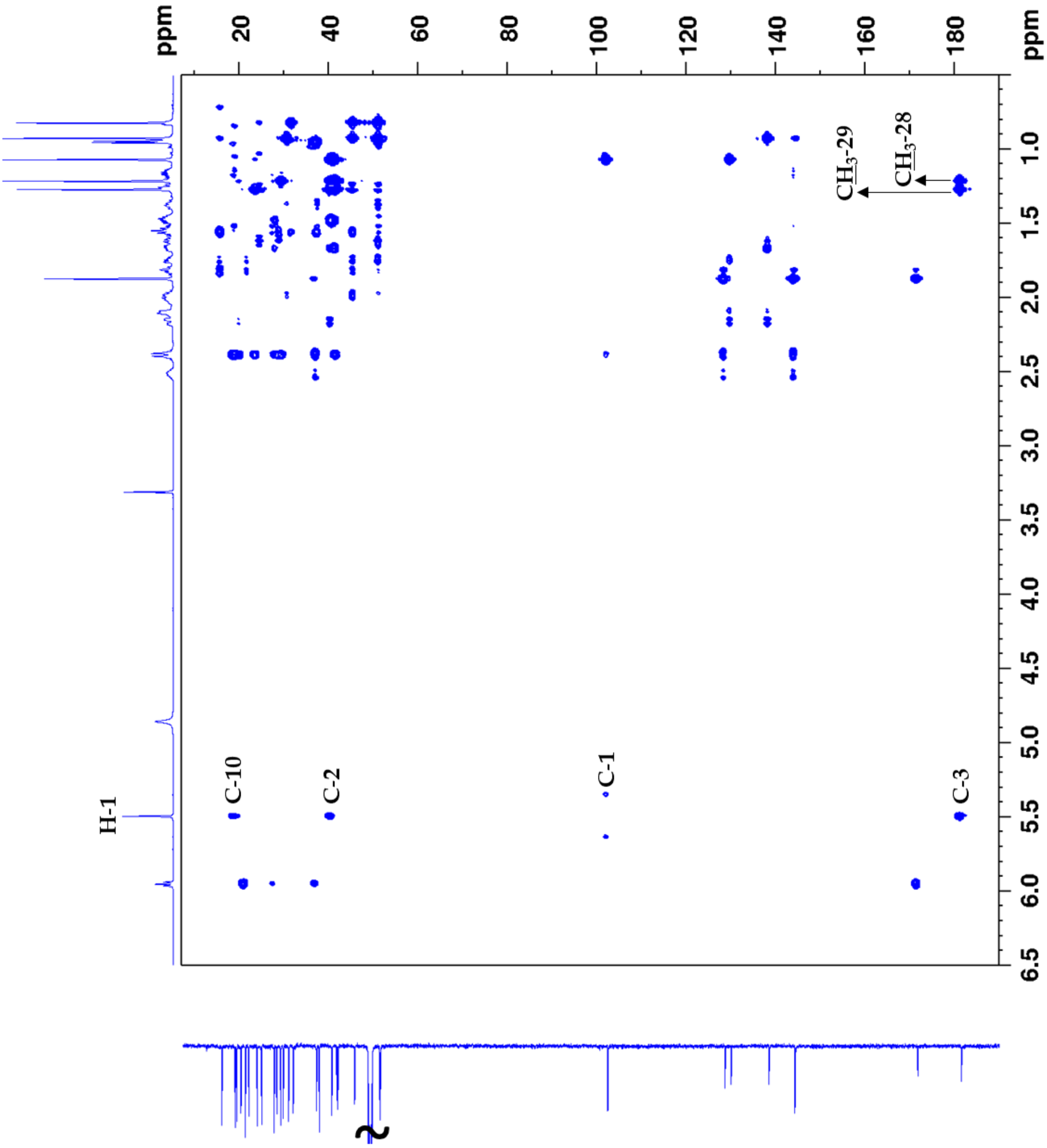

Figure S38: HMBC (600 MHz, MeOD) spectrum of **9**.

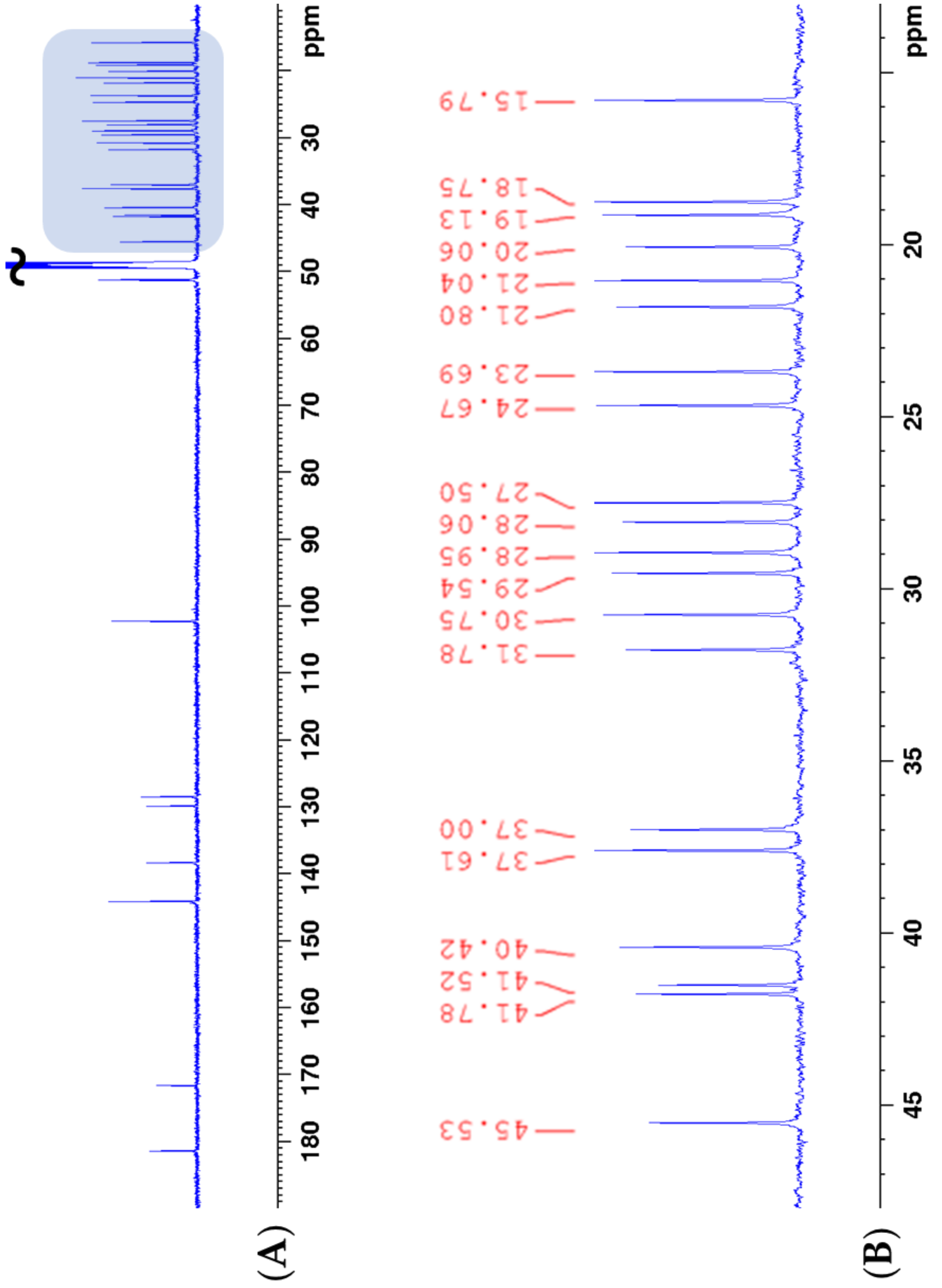

Figure S39: (A) ^{13}C (150 MHz, MeOD) spectrum of **9**. (B) Zoom region from 14.00 to 47.00 ppm.

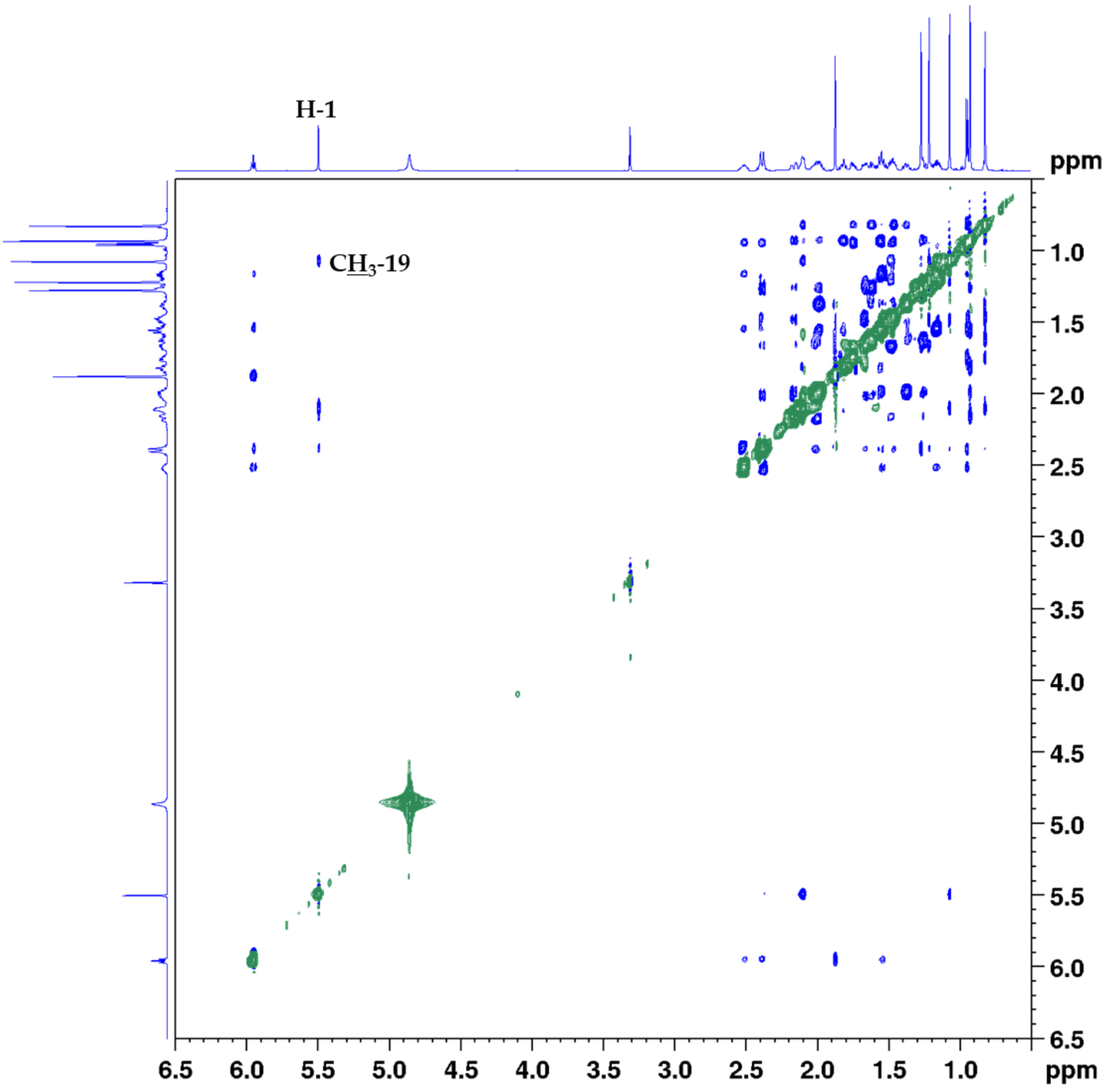

Figure S40: NOESY (600 MHz, MeOD) spectrum of **9**.

3β-hydroxy-1(2→3)-abeotirucalla-8,24Z-dien-26-oic acid (10)

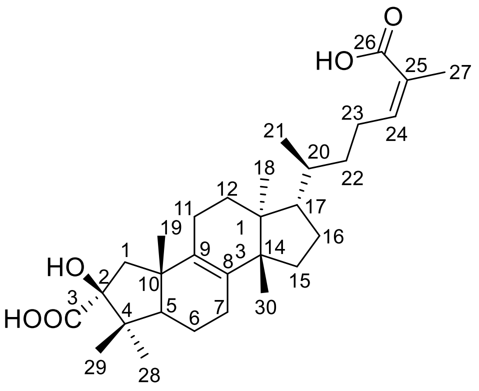

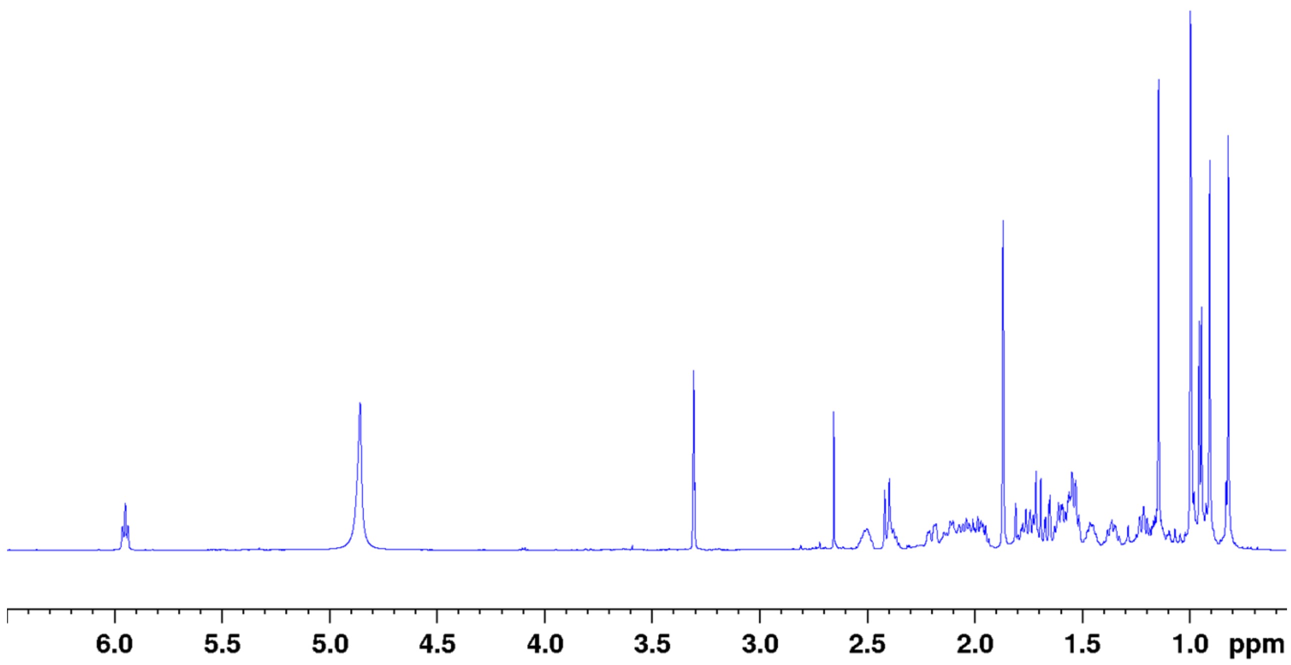

Figure S41: ^{1}H (600 MHz, MeOD) spectrum of **10**.

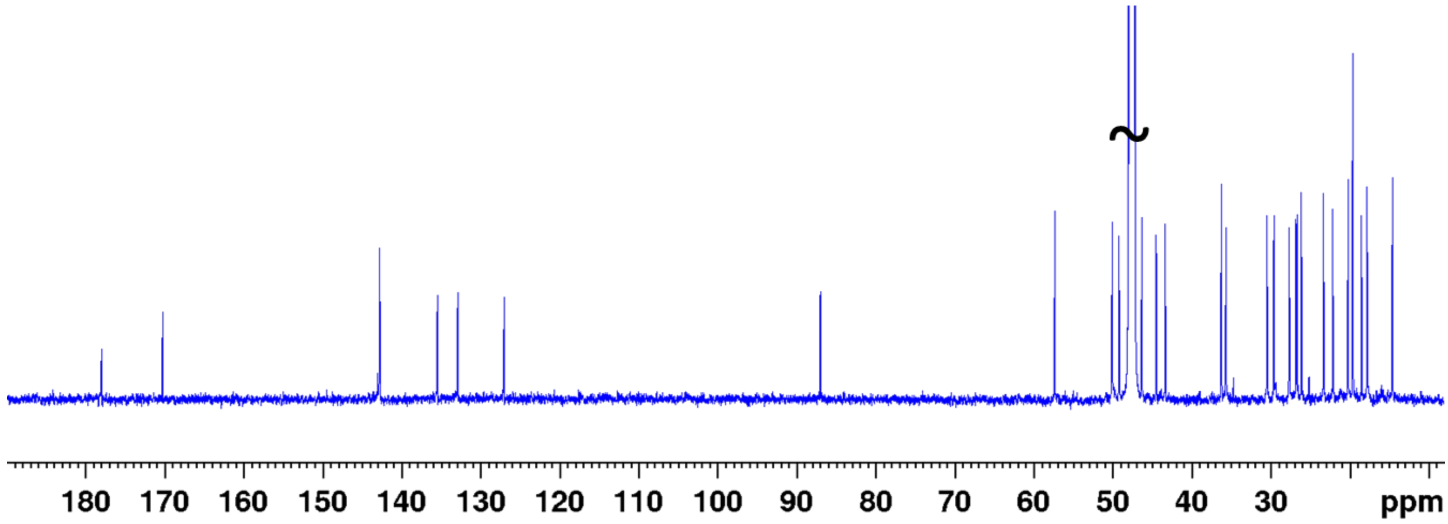

Figure S42: ^{13}C (150 MHz, MeOD) spectrum of **10**.

3-one-1(2→3)-abeotirucalla-8,24Z-dien-26-oic acid (11)

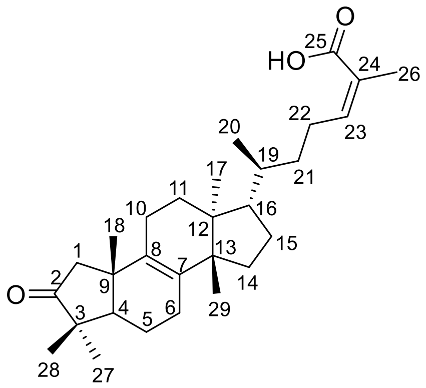

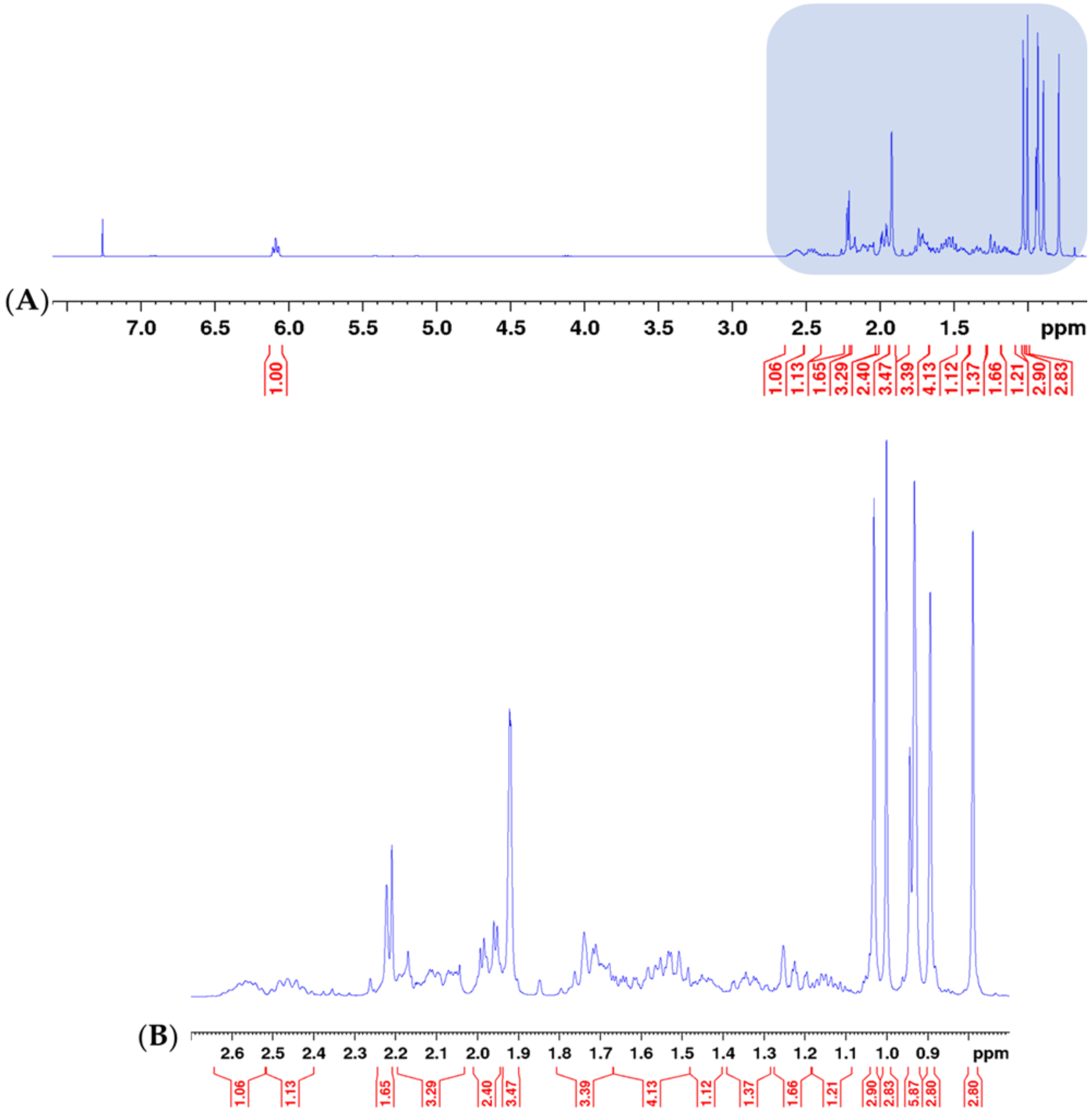

Figure S43: (A) ^{1}H (400 MHz, $CDCl_3$) spectrum of **11**. (B) Zoom region from 0.70 to 2.70 ppm.

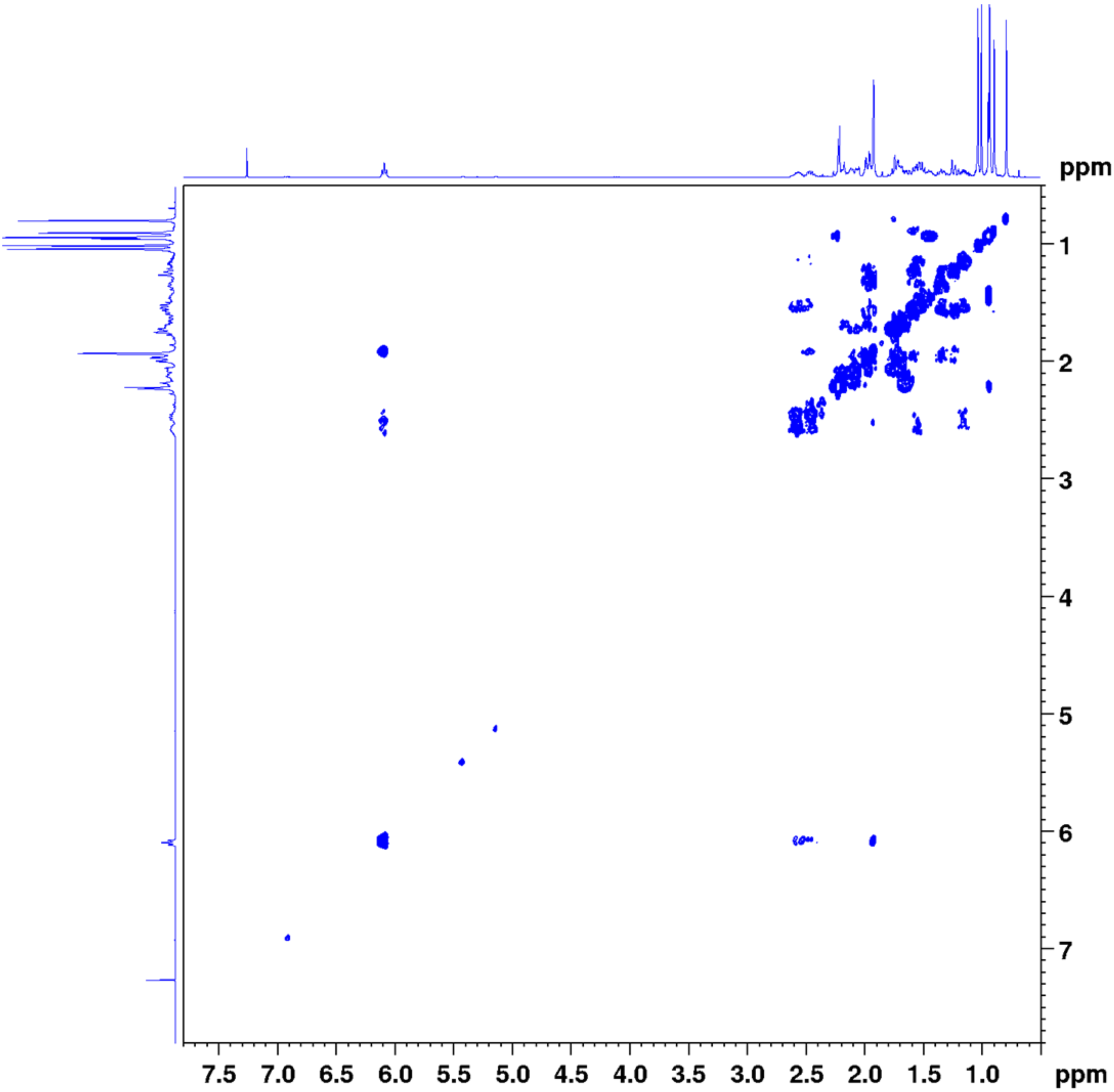

Figure S44: COSY (400 MHz, $CDCl_3$) spectrum of **11**.

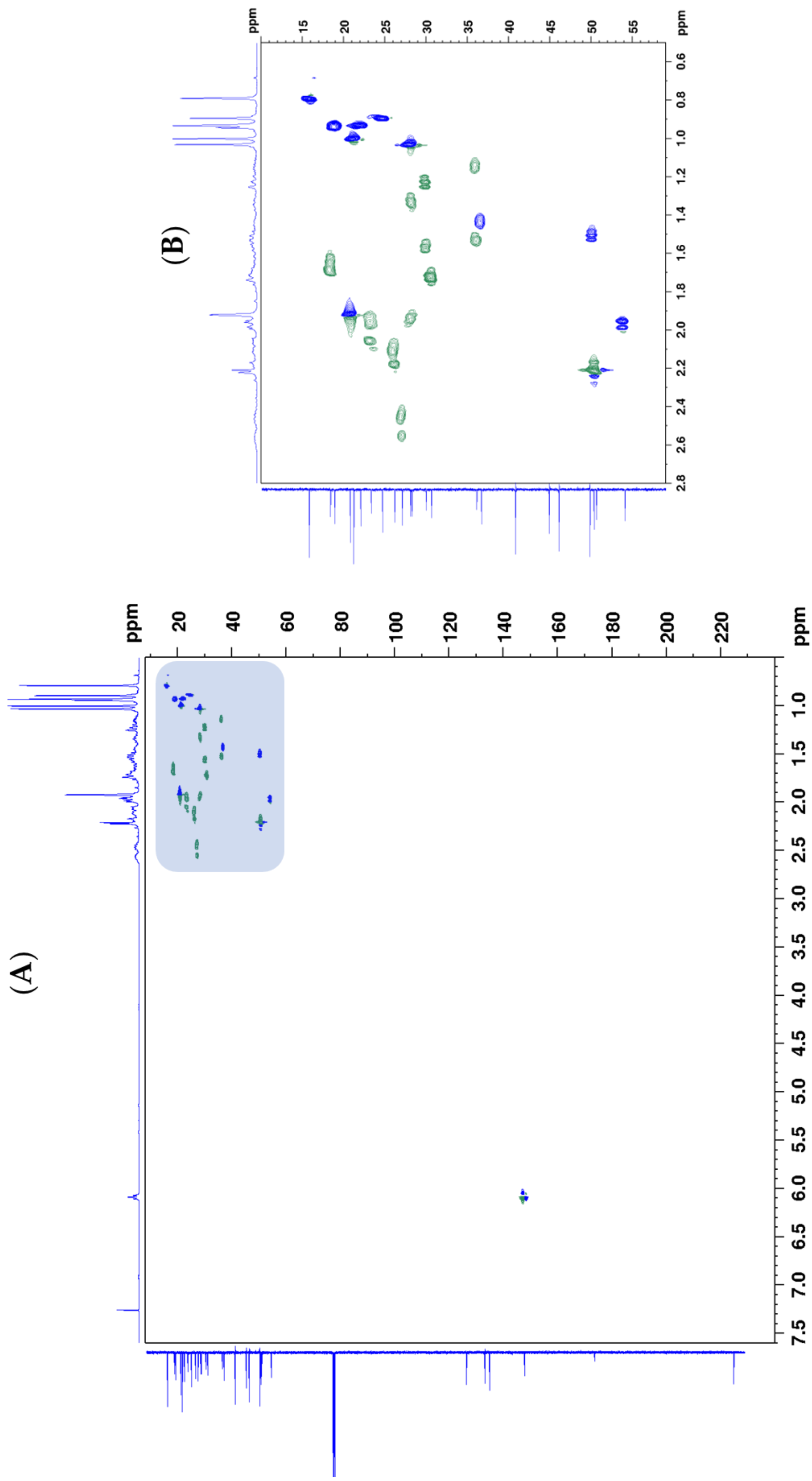

Figure S45: (A) HSQC (400 MHz, $CDCl_3$) spectrum of **11**. (B) Zoom region from 0.60 to 2.80 ppm.

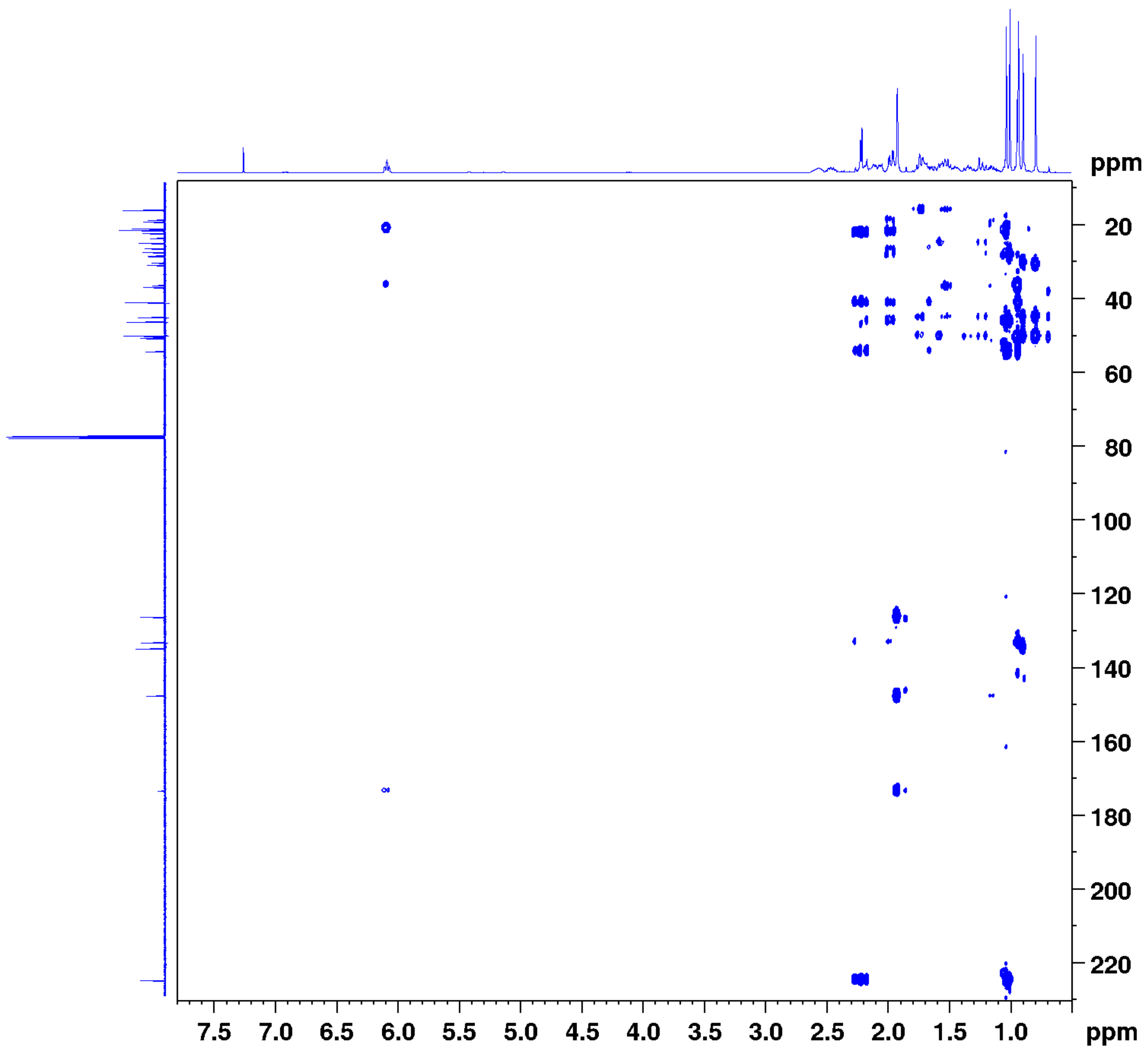

Figure S46: HMBC (400 MHz, $CDCl_3$) spectrum of **11**.

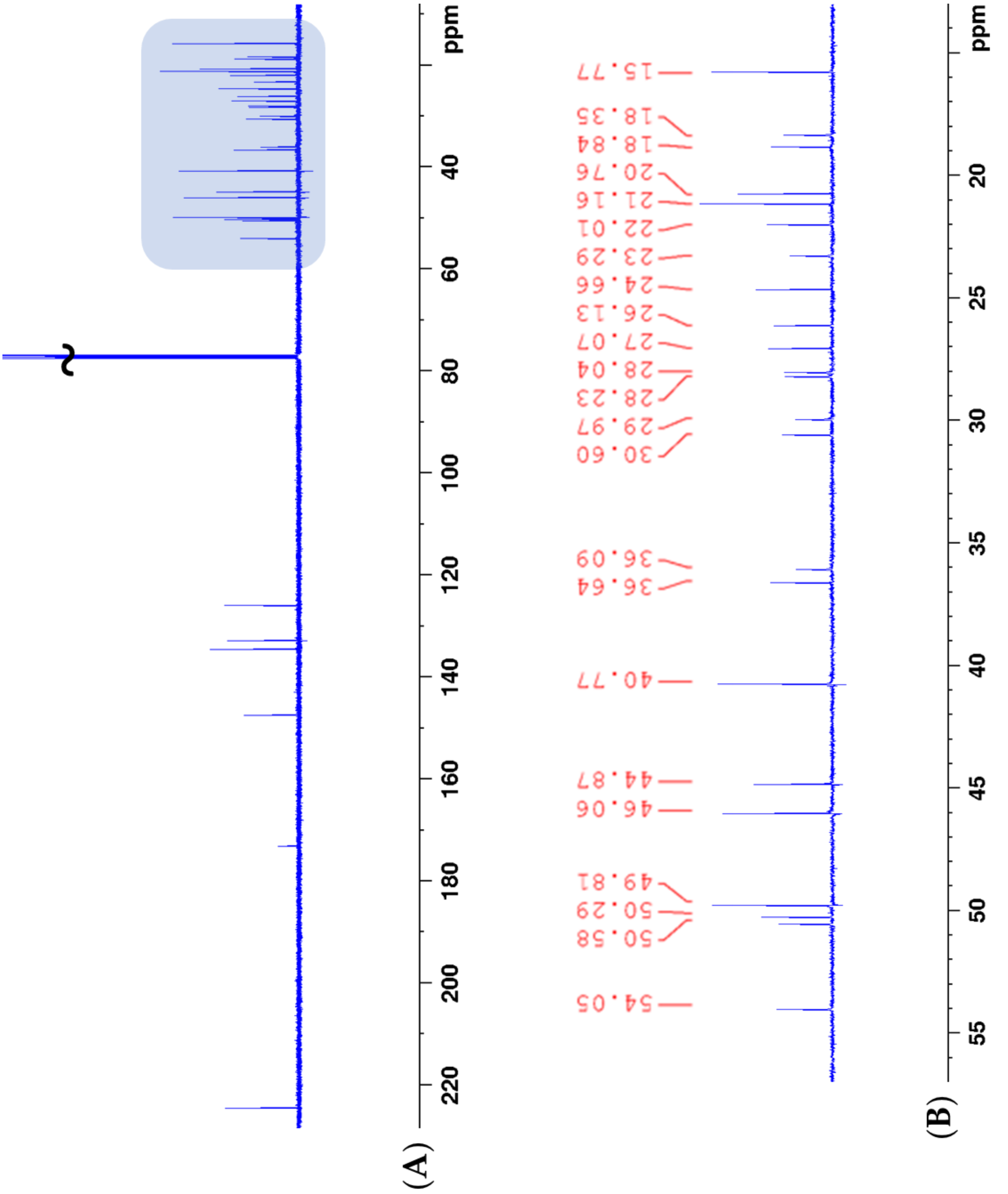

Figure S47: (A) ^{13}C (100 MHz, $CDCl_3$) spectrum of **11**. (B) Zoom region from 14.00 to 56.00 ppm.
